# End-to-End Bent Perylene Bisimide Cyclophanes by Double Sulfur Extrusion

**DOI:** 10.1021/jacs.4c05358

**Published:** 2024-05-30

**Authors:** Yuki Tanaka, Keita Tajima, Ryota Kusumoto, Yasuhiro Kobori, Norihito Fukui, Hiroshi Shinokubo

**Affiliations:** † Department of Molecular and Macromolecular Chemistry, Graduate School of Engineering, and Integrated Research Consortium on Chemical Science (IRCCS), Nagoya University, Furo-cho, Chikusa-ku, Nagoya, Aichi 464-8603, Japan; ‡ Department of Chemistry, Graduate School of Science, Kobe University, 1-1, Rokkodai-cho, Nada-ku, Kobe 657-8501, Japan; § Molecular Photoscience Research Center, Kobe University, 1-1, Rokkodai-cho, Nada-ku, Kobe 657-8501, Japan; ∥ CREST, JST, Honcho 4-1-8, Kawaguchi ,Saitama332-0012, Japan; ⊥ PRESTO, Japan Science and Technology Agency (JST), Kawaguchi ,Saitama332-0012, Japan

## Abstract

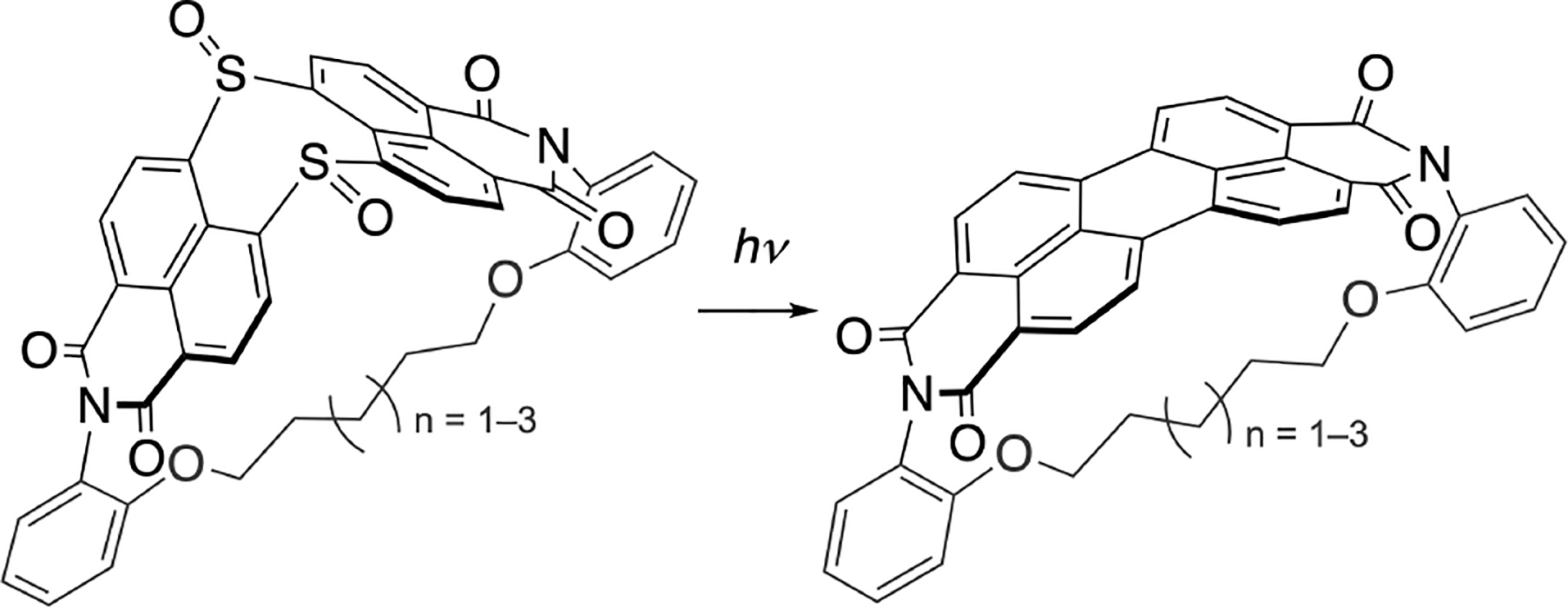

Bending inherently planar π-cores consisting of only six-membered rings has traditionally been challenging because a powerful transformation is required to compensate for the significant strain energy associated with bending. Herein, we demonstrate that sulfur extrusion can achieve substantial molecular bending of a perylene structure to form a substructure of a Vögtle belt, a proposed yet hitherto elusive carbon nanotube fragment. Bent perylene bisimide (PBI) derivatives were synthesized through a double-sulfur-extrusion reaction from the corresponding sulfur-containing V-shaped precursors with an internal alkyl tether. The effect of bending the inherently planar PBI core, which is a recent topic of interest for the design of advanced organic electronic and optoelectronic materials, was investigated systematically. Increasing the curvature leads to a red shift in the absorption and emission spectra, while the fluorescence quantum yields remain high. This stands in contrast with the nonemissive features of previously reported nonplanar PBI derivatives based on conjugative tethers. Detailed photophysical measurements indicated that the increasing curvature with shorter alkyl tethers (i) slightly facilitates intersystem crossing and (ii) significantly suppresses the internal conversion in the excited state of the present bent PBI derivatives. The latter characteristics originate from the restricted dynamic motion associated with the charge-transfer (CT) character between the core chromophores and the *N*-aryl units.

## Introduction

Recent progress in organic synthesis has led to the development of various nonplanar π-conjugated molecules, which had remained elusive targets until several decades ago due to the intrinsically planar nature of sp^2^-hybridized carbons.^1−13^ To date, there have been numerous reports on bowl-shaped and warped π-systems in which the incorporation of non-six-membered rings induces positive or negative Gaussian curvatures.^14−21^ However, examples of the coercive bending of inherently planar π-cores that consist of only six-membered rings, which have zero Gaussian curvature, remain limited because the synthesis of these contorted π-systems requires a powerful transformation to compensate for the significant strain energy associated with their curvature.

**Figure 1:**
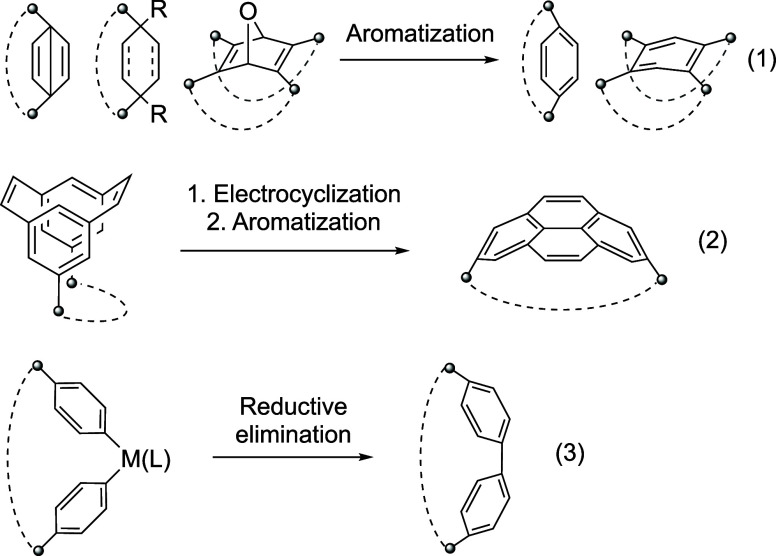
Strategies for the syntheses of bent π-systems.

Representative strategies for bending π-systems are summarized in Figure 1. [*n*]Paracyclophane, the simplest model of a curved π-system, has been synthesized from the Dewar benzene precursor via aromatization (eq 1).^22−24^ Bodwell and co-workers have reported the synthesis of π-extended cyclophanes such as pyrenophane and teropyrenophane via the electrocyclization of the corresponding cyclophanediene precursors and subsequent aromatization (eq 2).^25−29^ Cyclo[*n*]paraphenylenes have been prepared using two approaches: preconstruction of the main framework using sp^3^-hybridized carbon atoms (eq 1)^30,31^ and reductive elimination from less-strained organometallic intermediates (eq 3).^32,33^ The reductive-elimination strategy has also been employed to synthesize carbon nanobelts.^34^ Recently, zigzag carbon nanobelts have been synthesized via the reductive aromatization of an oxanorbornadiene segment as the key step (eq 1).^35,36^

**Figure 2:**
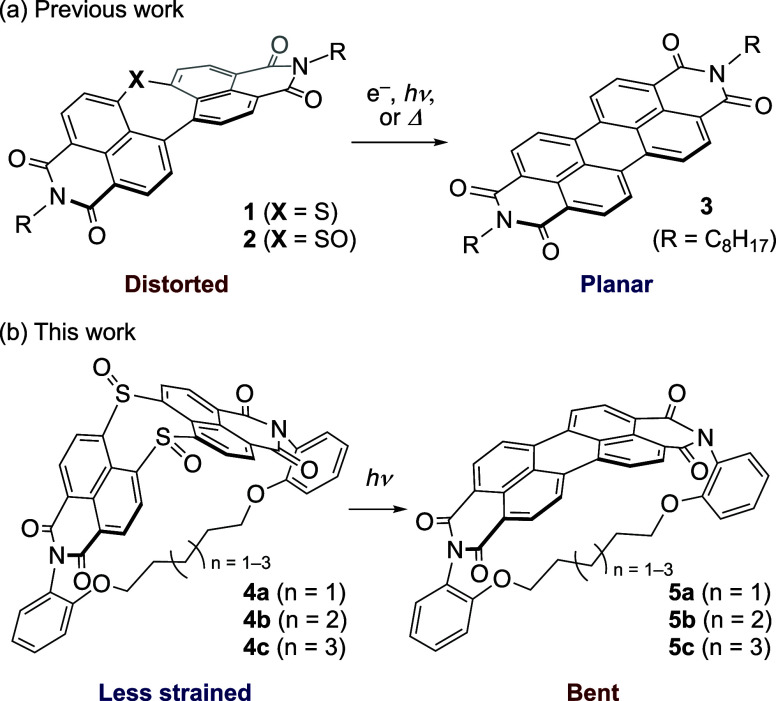
Synthesis of PBIs via sulfur-extrusion reactions from the corresponding precursors with inserted sulfur/sulfoxide units.

Our group has recently demonstrated that dinaphtho[1,8-*bc*:1′,8′-ef]thiepine bisimide (DNTBI) **1** and its sulfoxide (**2**) undergo sulfur extrusion upon electron injection, heating, or photoirradiation to yield PBI **3** almost quantitatively (Figure 2a).^37,38^ This transformation is accompanied by the release of strain from the distorted precursors. Here, we demonstrate that PBI derivatives with two inserted sulfoxide units undergo a double-sulfur-extrusion reaction (Figure 2b). This reaction was applied to bend an inherently planar PBI core; i.e., the photoirradiation of alkyl-tethered precursors **4a**–**4c** afforded the corresponding (perylene bisimide)phanes (PBIphanes) **5a**–**5c**. The curved perylene unit represents a fragment of a Vögtle belt, which is a proposed yet hitherto elusive carbon nanotube fragment.^39^ The molecular design of **5a**–**5c** was inspired by Bodwell’s pyrenophanes.^7,27−29^ While ring-contraction via sulfur extrusion from a C(sp^3^)–S–C(sp^3^) unit is a representative strategy to create cyclophanes,^22^ the synthesis of strained molecules by sulfur extrusion from a C(sp^2^)–S–C(sp^2^) unit remains so far unprecedented.^40^

**Figure 3:**
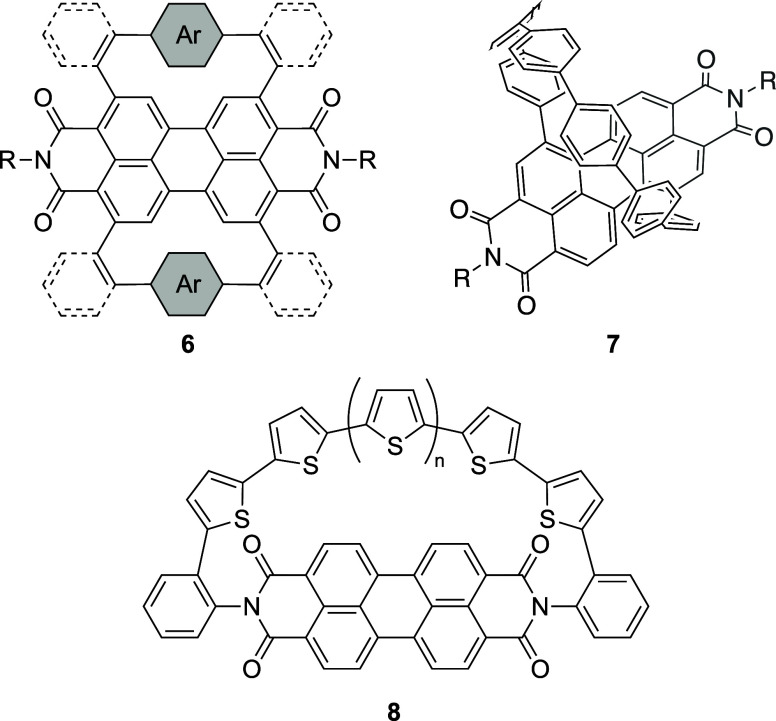
Structures of previously reported PBIphanes **6**, **7**, and **8**.

Figure 3 presents the previously reported PBIphanes **6**–**8**. Xiao and co-workers have reported **6**, which adopts contorted structures induced by π-conjugative linkers.^41^ Oligoparaphenylene-tethered PBIphane **7**(42) and oligothiophene-tethered PBIphanes **8**(43) adopt twisted and planar PBI cores, respectively. Our PBIphanes **5a**–**5c** contain nonconjugative alkyl tethers to induce the curvature of the π-system. Hence, **5a**–**5c** should be more suitable candidates to explore the effects of the bending of inherently planar PBIs^44−47^ on the chemical and physical properties of the π-conjugated cores, which has been a recent topic of high interest for the design of advanced organic electronic and optoelectronic materials.^48−50^ Twisted PBI derivatives with alkyl tethers have been studied by Würthner and co-workers.^51,52^

## Results and Discussion

### Synthesis and Sulfur-Extrusion Reactions of Model Compounds

**Scheme 1:**
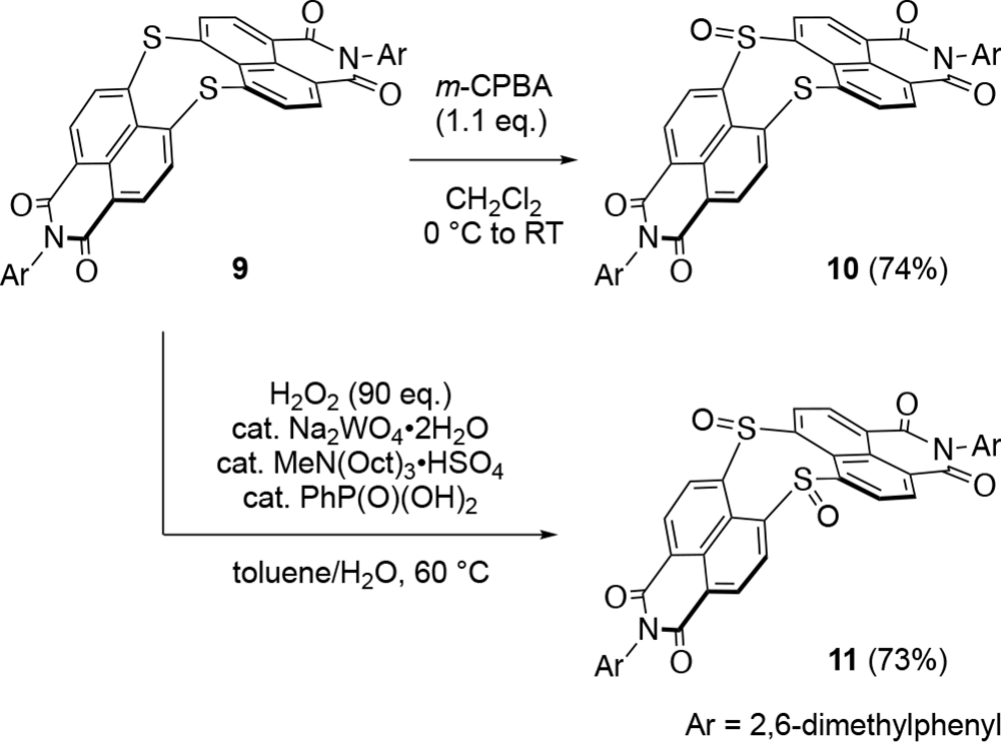
Synthesis of Sulfoxide Derivatives of DNDTBI

Dinaphtho[1,8-*bc*:1′,8′-*fg*][1,5]dithiocine bisimide (DNDTBI) **9**(53) was subjected to oxidation with *m*-chloroperbenzoic acid (*m*-CPBA, 1.1 equiv) to afford the corresponding sulfoxide **10** in 74% yield (Scheme 1). The tungsten-catalyzed oxidation^54^ of **9** with hydrogen peroxide provided disulfoxide **11** in 73% yield.

**Figure 4:**
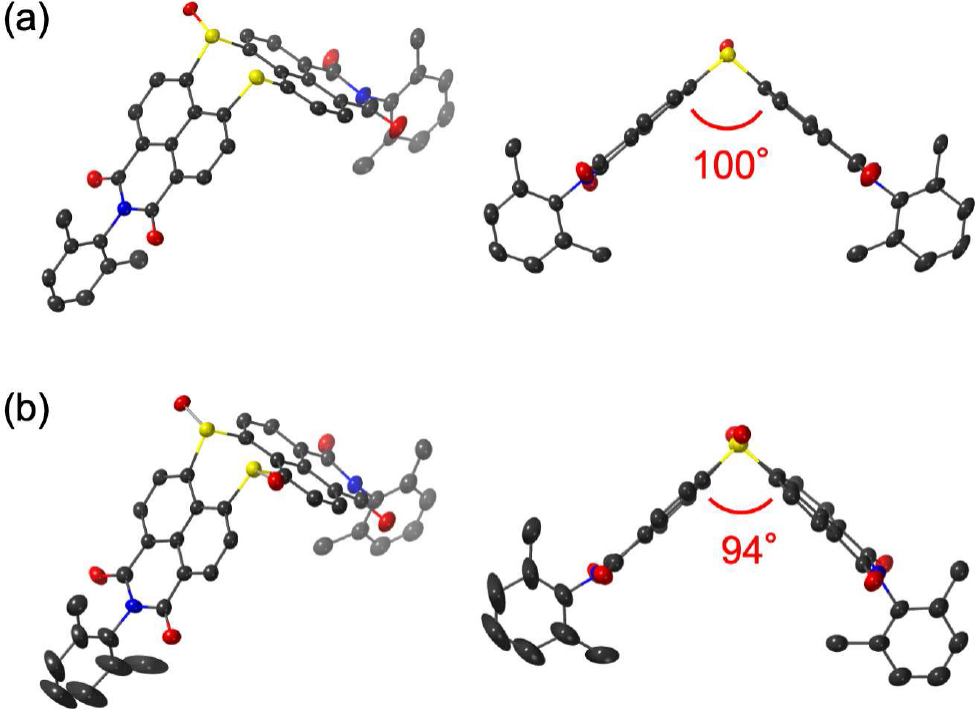
X-ray crystal structures of (a) **10** and (b) **11** with thermal ellipsoids at 50% probability; all hydrogen atoms have been omitted for the sake of clarity.

The solid-state structures of **10** and **11** were unambiguously determined by using single-crystal X-ray diffraction analysis (Figure 4). In the crystal, **10** and **11** adopt a V-shaped structure similar to that of **9**. The interplanar angles between the two naphthalene monoimide units gradually decrease in the order **9** (113°) > **10** (100°) > **11** (94°) due to the smaller bond angle of sulfoxide compared to that of sulfide. The distances between the *ortho*-substituents of the *N*-aryl groups of **9**, **10**, and **11** are 11.0, 9.2–9.8, and 8.2 Å, respectively. These values are comparable to the O–O distance of 1,6-hexanediol (8.7 Å) and longer than that of 1,5-pentanediol (7.3 Å).

**Figure 5:**
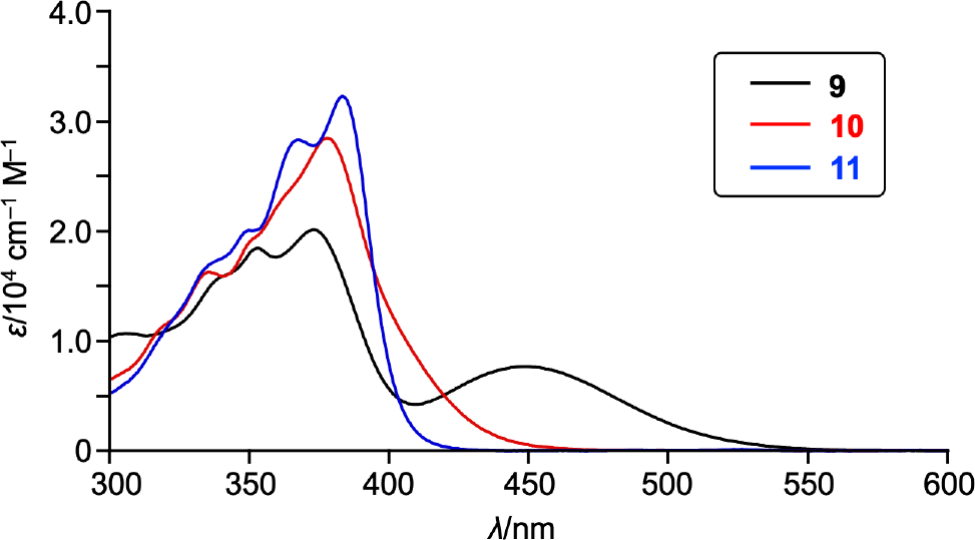
UV/vis absorption spectra of **9**–**11** in CH_2_Cl_2_; λ, wavelength; ε, extinction coefficient.

The UV/vis absorption spectra of **9**–**11** are shown in Figure 5. DNDTBI **9** exhibits a broad absorption band at 450 nm, which was assigned to the charge-transfer (CT) transition from the central sulfur atoms to the naphthalene monoimide units.^53^ Accordingly, monosulfoxide **10** shows hypsochromically shifted absorption with a shoulder peak tailing to 460 nm. Furthermore, the CT transition completely disappeared in the absorption spectrum of **11**, with the remaining sharp absorption bands tailing at 420 nm.

**Figure 6:**
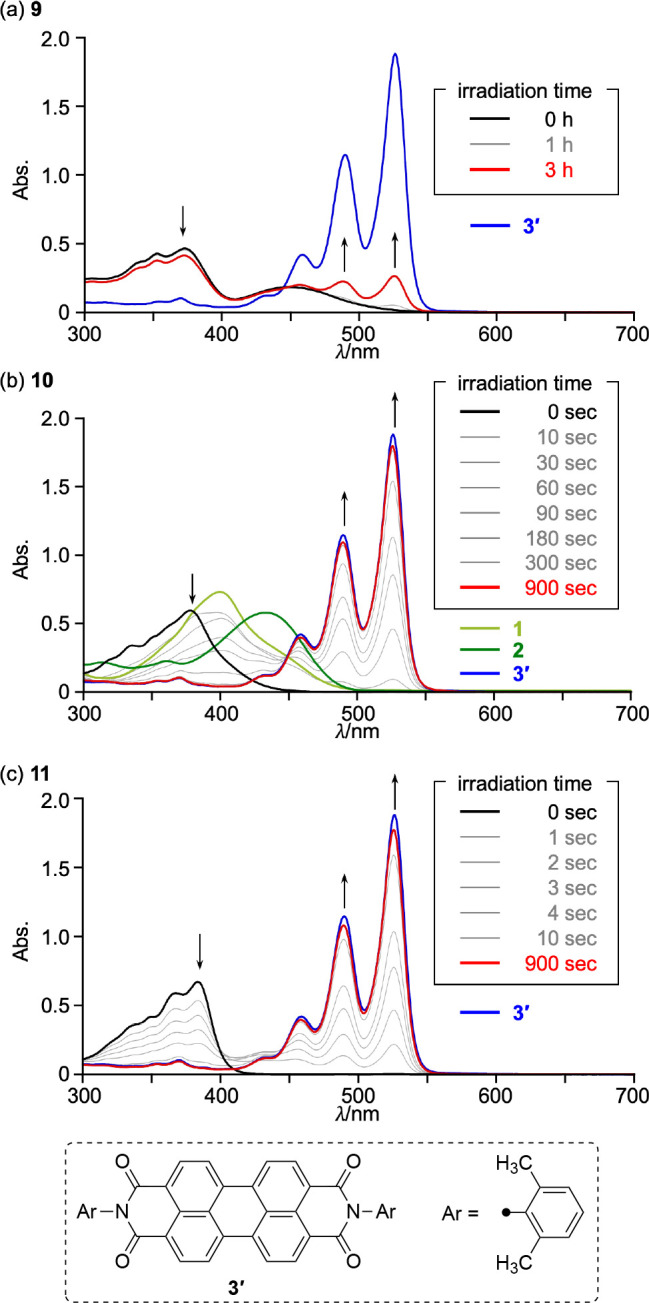
Changes in the UV/vis absorption spectra of (a) **9**, (b) **10**, and (c) **11** upon photoirradiation in CH_2_Cl_2_. A high-pressure mercury lamp equipped with a sharp cutoff filter (λ > 380 nm) was employed for the photoirradiation; [**1**] = [**2**] = [**3′**] = [**9**]_0_ = [**10**]_0_ = [**11**]_0_ = 2.1 × 10^–5^ M^–1^.

Subsequently, we examined the photoinduced sulfur-extrusion reactions of **9**–**11** in CH_2_Cl_2_ (Figure 6). Photoirradiation was performed using a high-pressure mercury lamp equipped with a sharp cutoff filter (λ > 380 nm). Compound **9** underwent the sulfur-extrusion reaction, albeit sluggishly, to afford PBI **3′** in only 19% yield even after an extended photoirradiation period of 3 h. In contrast, the sulfur-extrusion reaction of **10** afforded PBI **3′** almost quantitatively within 900 s. Notably, a broad absorption band appeared at 370–450 nm during the irradiation of **10**. This feature resembles the spectrum of DNTBI **1** rather than that of its sulfoxide **2**. These results suggest that the sulfoxide unit rather than the sulfide unit of **10** was removed preferentially, which is in agreement with the observed trend for **1** and **2**, i.e., the sulfur-extrusion reaction of **2** proceeded more rapidly than that of **1**.^38^ Furthermore, the sulfur-extrusion reaction of **11** was faster than that of **10**, reaching completion to give **3′** in approximately 30 s. After 15 min of photoirradiation, the absorption spectrum is almost identical to that of PBI **3′**, indicating nearly quantitative conversion. The lack of isosbestic points can be attributed to the formation of DNTBI sulfoxide **2** as an intermediate.

We also examined the sulfur-extrusion reaction of **11** via electron injection or heating. The cyclic voltammograms of **11** in CH_2_Cl_2_ (Figure S39) feature an irreversible reduction wave at −1.38 V in the forward direction. Upon backsweeping, two peaks were observed at −0.97 and −1.16 V, which are identical to those of PBI **3′**. Furthermore, spectroelectrochemical measurements of **11** revealed that the absorption spectrum of **11** after electrochemical reduction is identical to that of the radical anion of PBI **3′** (Figure S40). These results indicate that electron injection triggers the extrusion of sulfur from **11**, as was observed for the previously reported DNTBI sulfoxide **2**. On the other hand, the thermogravimetric analysis (TGA) profile of **11** exhibits no characteristic mass decrease due to sulfur extrusion while DNTBI sulfoxide **2** underwent sulfur extrusion upon heating (Figure S42).

### Synthesis of PBIphanes

**Scheme 2:**
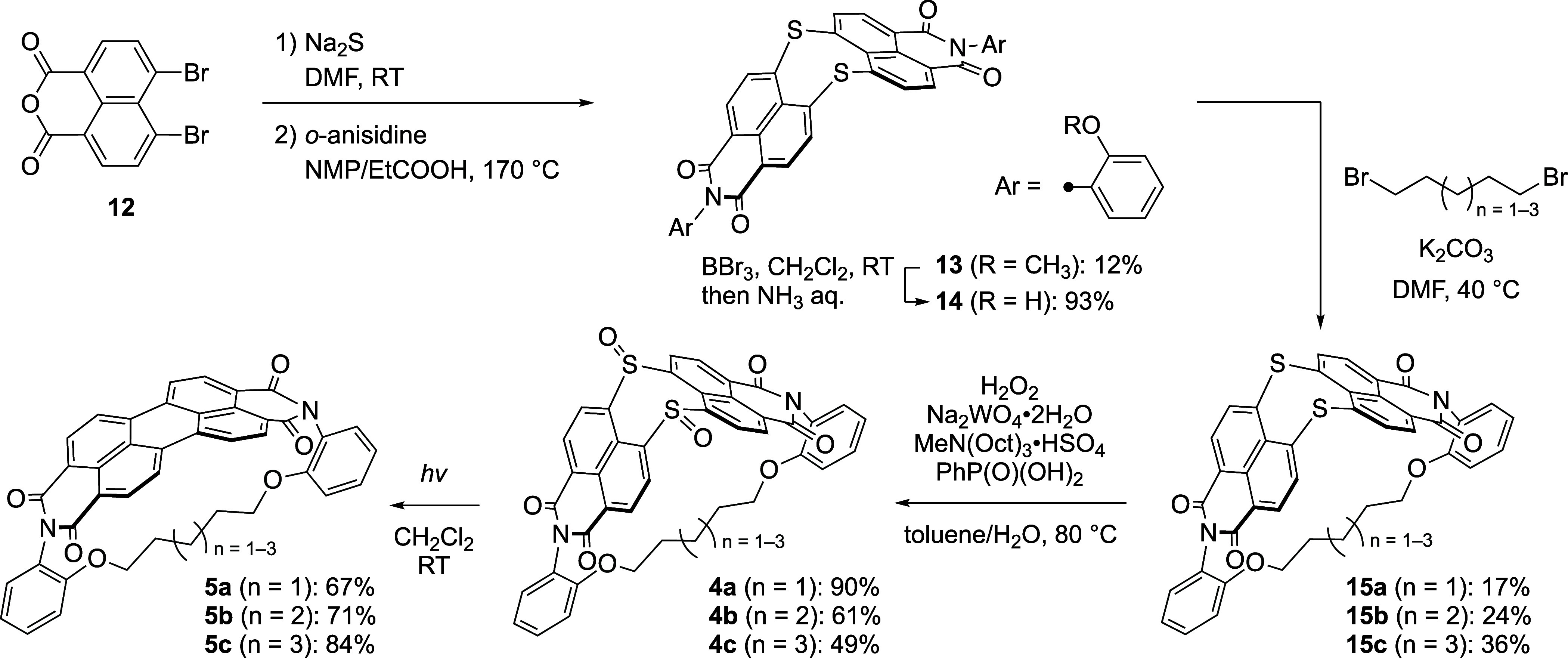
Synthesis of PBIphanes **5a–5c**

The synthesis of PBIphanes **5a**–**5c** is shown in Scheme 2. In our previous procedure, a 4,5-dibromonaphthalene monoimide derivative was subjected to a nucleophilic aromatic substitution (S_N_Ar) reaction with Na_2_S to afford DNTBI **9**.^53^ Here, we attempted to treat 4,5-dibromo-1,8-naphthalic anhydride **12** with *o*-anisidine; however, this resulted in an undesired competing S_N_Ar reaction at the bromo substituents. We thus conducted the initial S_N_Ar reaction of **12** with Na_2_S and subsequent treatment with *o*-anisidine afforded *o*-anisyl DNTBI **13** in 12% yield. The aryl oxygen groups of **13** were deprotected with BBr_3_ to furnish 2-hydroxyphenyl DNTBI **14** in 93% yield. Treatment of **14** with 1,5-dibromopentane, 1,6-dibromohexane, or 1,7-dibromoheptane provided the corresponding internally tethered products **15a**, **15b**, and **15c** in 17, 24, and 36% yield, respectively. The sulfide units of **15a**, **15b**, and **15c** were converted to sulfoxides through tungsten-catalyzed oxidation to give **4a**, **4b**, and **4c** in 90, 61, and 49% yield, respectively. Finally, photoirradiation of **4a**, **4b**, and **4c** with a high-pressure mercury lamp afforded the desired PBIphanes **5a**, **5b**, and **5c** in 67, 71, and 84% yield, respectively.

**Figure 7:**
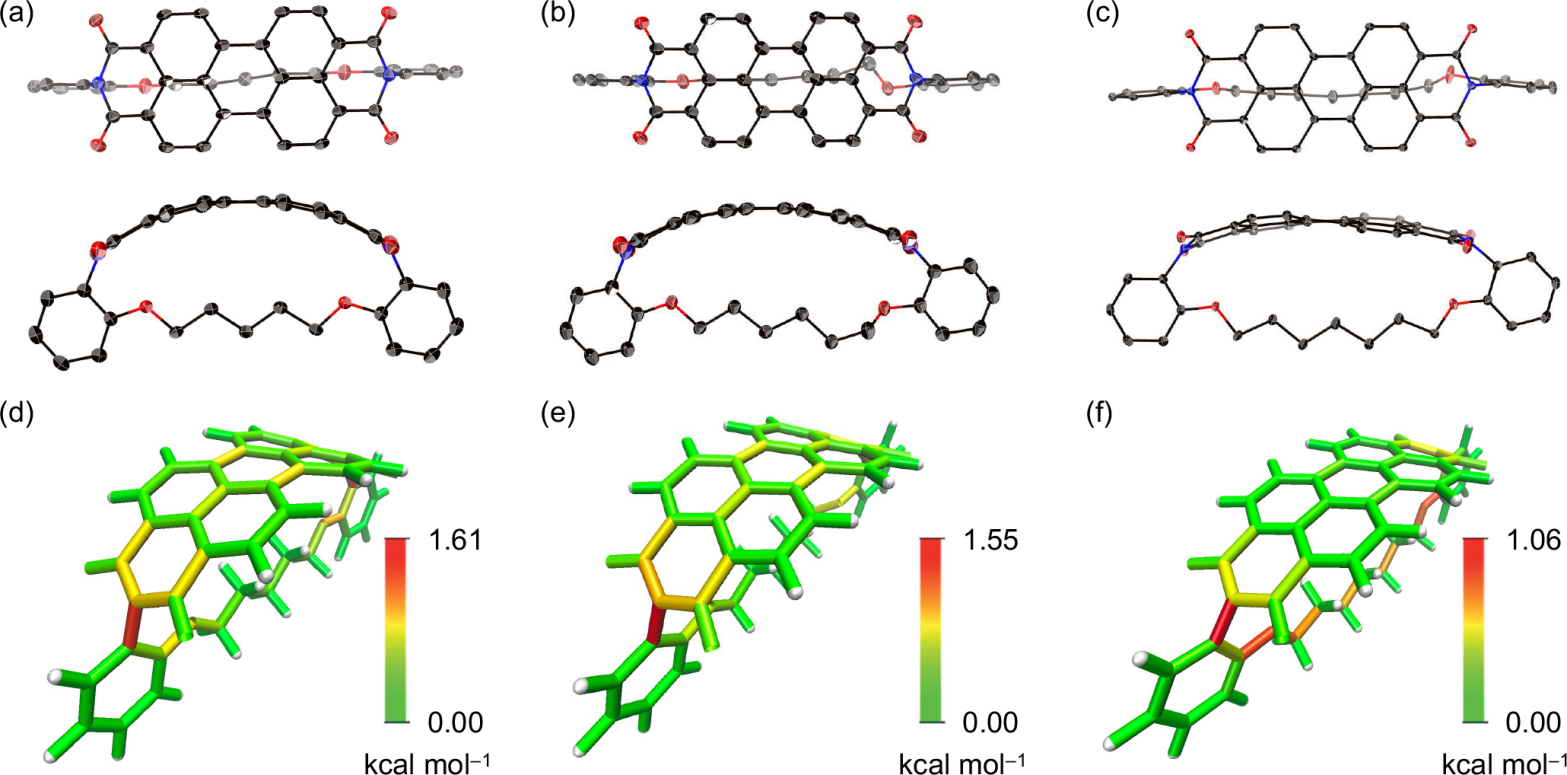
X-ray crystal structures (top: top view; bottom: side view) of (a) **5a**, (b) **5b**, and (c) **5c** with thermal ellipsoids at 50% probability; all hydrogen atoms are omitted for clarity. Visualized local strains in (d) **5a**, (e) **5b**, and (f) **5c** were calculated at the B3LYP/6-31G(d) level.

The structures of PBIphanes **5a**–**5c** were unambiguously determined by X-ray diffraction analysis (Figure 7a–c). Structural parameters are summarized in Table 1. In the single crystal, PBIphanes **5a**, **5b**, and **5c** adopt bent structures with nitrogen–nitrogen distances, *d*, of 10.55, 10.78, and 11.15 Å, respectively. These nitrogen–nitrogen distances are significantly shorter than that of planar PBI **3** (*d* = 11.3 Å)^55^ and comparable to those of previously reported contorted PBIphanes **6** (*d* = 10.25–11.36 Å). The end-to-end bend angles, θ, are 33.8° (**5a**), 29.4° (**5b**), and 19.4° (**5c**). In the X-ray crystal structure of **5b**, one methylene unit neighboring an oxygen atom adopts a gauche configuration, which can be attributed to the mismatch due to having an even-numbered alkyl chain as the tethering unit. The π-orbital-axis-vector (POAV) angles,^56^ which provide a measure of the degree of orbital hybridization based on structural aspects, were calculated based on the crystal structures of **5a**–**5c** (Table 1), in which the obtained POAV angles at the equivalent carbons were averaged. The POAV angles increase in the order **5c** < **5b** < **5a**, indicating that increased curvature decreases the s-character of the constituent carbon atoms. The deviation from planarity is most pronounced at the imide-substituted carbon atoms (position a).

**Table 1:**
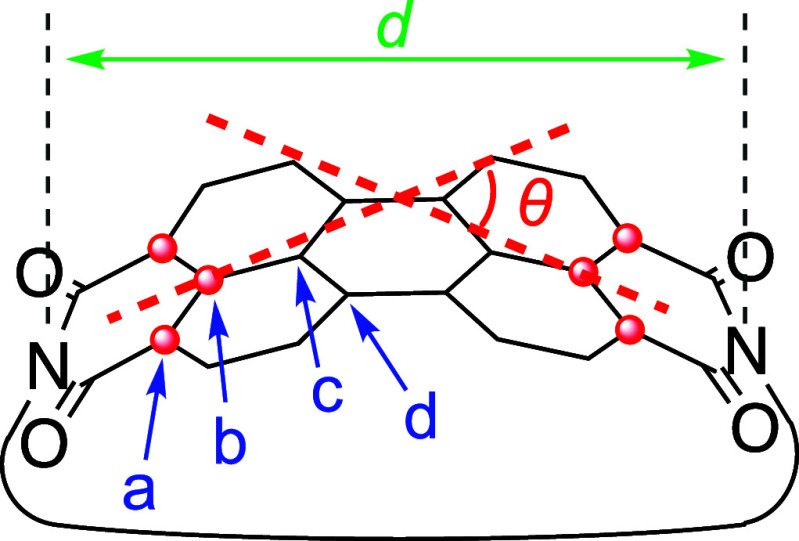
Structural Parameters and Calculated Strain Energies for **5a–5c**

	*d*^a^/Å	θ^b^/°	POAV angle/°				*E*^c^/kcal mol^–1^
			*a*	*b*	*c*	*d*	
**5a**	10.55	33.8	3.2	1.2	1.2	2.2	22.9
**5b**	10.78	29.4	2.6	1.2	1.5	2.0	19.7
**5c**	11.15	19.4	2.0	0.6	0.7	1.0	17.4

a Nitrogen–nitrogen distance.

b End-to-end bend angle.

c Strain energy.

The strain energies in **5a**, **5b**, and **5c** were investigated using hypothetical homodesmotic reactions at the B3LYP/6-31G(d) level and were calculated to be 22.9, 19.7, and 13.6 kcal mol^–1^, respectively (Figure S45). The local strain energies were also visualized using the StrainVis tool (Figure 7d–f).^57^ The PBIphanes **5a**, **5b**, and **5c** exhibit the greatest local strain at the C–N bond of the *N*-aryl substituents, which increases with decreasing length of the alkyl linker (**5a**: 1.61 kcal mol^–1^; **5b**: 1.55 kcal mol^–1^; **5c**: 1.06 kcal mol^–1^). In the perylene core, the strain is mainly localized at the peripheral bonds around the naphthalene segments, as was also observed in a computational simulation of a Vögtle belt.^57^ The C–O bonds in **5a**–**5c** also contribute substantially to the accommodation of the local strain.

The solubility value of **5a** in CHCl_3_ (0.11 mg/mL) is higher than those of **5b** (0.027 mg/mL) and **5c** (0.045 mg/mL), due to the large molecular curvature. However, these solubility values of PBIphanes **5a**–**5c** are smaller than that of planar *N*-(2-methoxyphenyl)-substituted PBI **3″** (0.38 mg/mL), which exhibits high solubility due to the existence of rotamers. The relatively low solubility of PBIphanes **5a**–**5c** is attributable to the restricted rotation of their *N*-substituents.

### Aromaticity of PBIphanes

In the ^1^H NMR spectra of **5a**–**5c** and untethered planar PBI **3″** in tetrachloroethane-*d*_2_, the signals of the aromatic protons showed an upfield shift with increasing curvature (**5a**: 8.64 and 8.62 ppm; **5b**: 8.67 and 8.65 ppm; **5c**: 8.71 and 8.69 ppm; **3″**: 8.74 and 8.72 ppm). DFT calculations reproduced this trend (Table S3). Nevertheless, the nucleus-independent chemical shift (NICS) values at the carbonyl-substituted six-membered rings of **5a**–**5c** and **3″**, which represent an averaged value among four rings, are almost comparable (**5a**: −6.44 ppm; **5b**: −6.44 ppm; **5c**: −6.52 ppm; **3″**: −6.61 ppm). Consequently, we conclude that (i) increasing the molecular curvature in PBI has a negligible effect on the aromaticity and (ii) the observed upfield shifts of the aromatic proton signals originate from the change in orientation toward the neighboring carbonyl group or aromatic ring.

### Electrochemical Properties of PBIphanes

The cyclic voltammograms and differential pulse voltammograms of **5a**–**5c** and **3″** were measured in CH_2_Cl_2_ by using 0.1 M Bu_4_NPF_6_ as the supporting electrolyte and Ag/AgNO_3_ as the reference electrode (Figure S41). The ferrocene/ferrocenium couple (Fc/Fc^+^) was used as an internal reference. For **5a**–**5c** and **3″**, two reversible reduction waves (**5a**: −1.03 and −1.27 V; **5b**: −1.01 and −1.24 V; **5c**: −1.00 and −1.23 V; and **3″**: −1.03 and −1.22 V) were observed. These results indicate that the sensitivity of the CV measurements is insufficient to distinguish the subtle effect of molecular bending on the electron-accepting abilities.

### Photophysical Properties of PBIphanes

**Figure 8:**
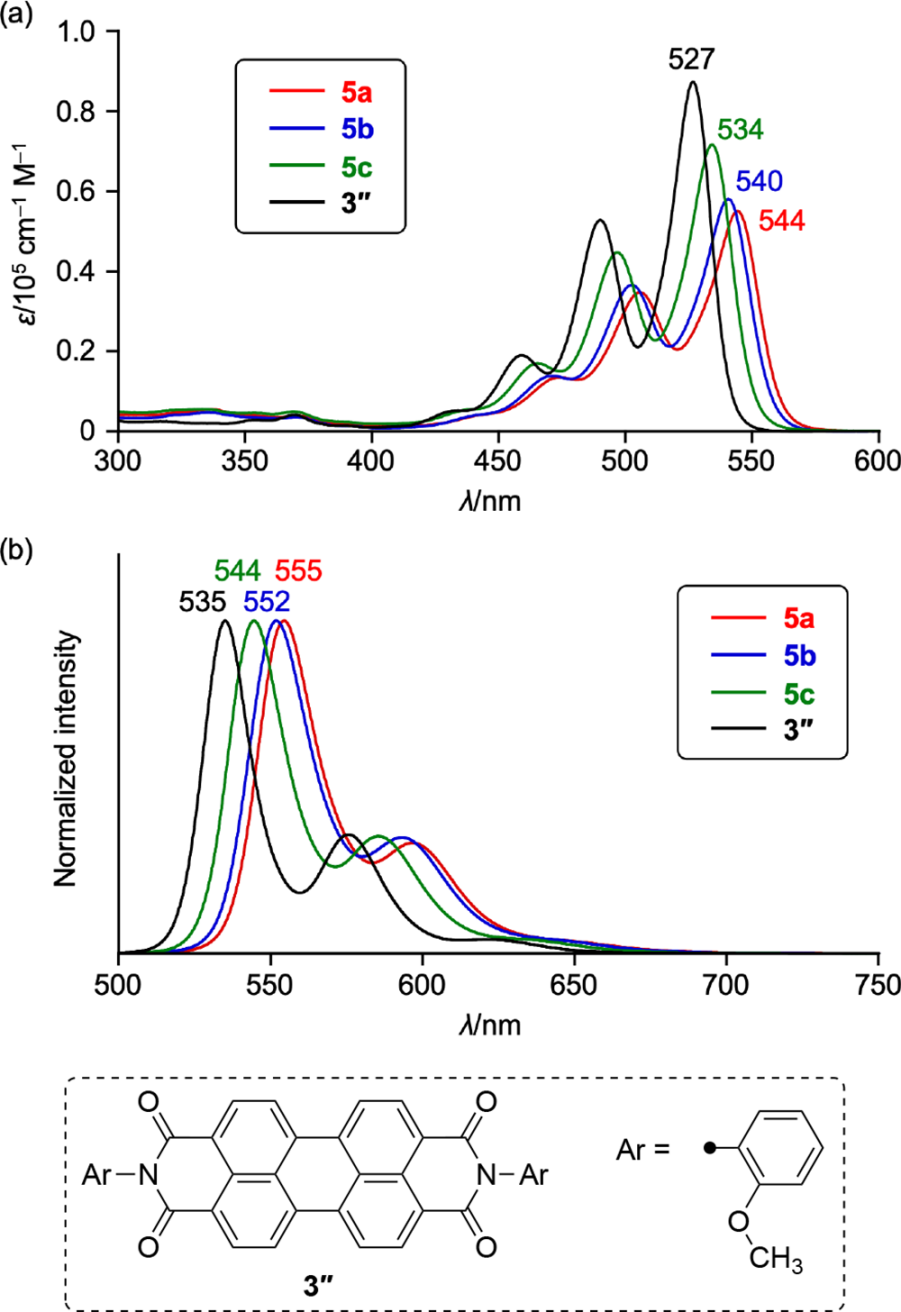
UV/vis (a) absorption and (b) emission spectra of **3″**, **5a**, **5b**, and **5c** in CHCl_3_; λ, wavelength; and ε, extinction coefficient.

The UV/vis absorption and emission spectra of PBI **3″** and PBIphanes **5a**–**5c** are shown in Figure 8, and their photophysical parameters are summarized in Table 2. Increasing the structural curvature of the PBI cores results in a red shift of the longest-wavelength absorption peaks from 527 nm (**3″**) to 544 nm (**5a**), together with decreased extinction coefficients. This trend contrasts with those of pyrenophane and teropyrenophane, in which increased structural curvature leads to blue-shifted absorption spectra.^26,28^ These results firmly corroborate the recent argument that the symmetry of the local orbitals in the K-region (bonding or antibonding) governs the (de)stabilization of the HOMO and LUMO.^50^ The shortest-wavelength emission peaks also exhibited a bathochromic shift from 535 nm (**3″**) to 555 nm (**5a**) with increasing curvature. The PBIphanes exhibited intense emission with quantum yields, Φ_F_, of 0.84–0.88, which are larger than that of planar PBI **3″** (Φ_F_ = 0.76). It is worth noting here that the previously reported π-tethered contorted PBI derivatives **6** exhibit behavior that is different from that of the present PBIphanes **5a**–**5c**: (i) negligible correlation between the absorption wavelength and molecular bending and (ii) weak emission (Φ_F_ = 0.01–0.32)^41^; these differences highlight the fact that the electronic properties of **6** are substantially altered by the π-delocalization onto the tethering π-linkers.

**Table 2:** Photophysical Parameters of **3″** and **5a–5c**

	Δν^a^/cm^–1^	Φ_F_^b^	Φ_ISC_^c^	τ_F_^d^/ns	*k*_r_^e^/10^8^ s^–1^	*k*_nr_^f^/10^8^ s^–1^
**5a**	364	0.88		4.9	1.8	0.24
**5b**	403	0.84	0.001	4.6	1.8	0.35
**5c**	344	0.84	0.002	4.1	2.0	0.39
**3″**	284	0.76		3.6	2.1	0.67

a Stokes shift.

b Fluorescence quantum yield.

c ISC quantum yield.

d Fluorescence lifetime.

e Radiative decay constant.

f Nonradiative decay constant.

The full widths at half-maximum (fwhm’s) of the 0–0 absorption bands of **5a**, **5b**, **5c**, and **3″** calculated from the Gauss fitting are 659, 686, 661, and 601 cm^–1^, respectively. The Stokes shifts of **5a**, **5b**, **5c**, and **3″** are 364, 403, 344, and 284 cm^–1^, respectively. The larger fwhm and Stokes shift values of PBIphanes **5a**–**5c** compared to those of planar PBI **3″** were attributed to their nonplanar structures. Interestingly, the fwhm and Stokes shift values of **5b** are larger than those of **5a** and **5c**, suggesting that the mismatch of its even-numbered alkyl chain increases the structural flexibility.

The photophysical parameters of **5a**–**5c** and **3** are summarized in Table 2. Their radiative decay rates, *k*_r_, decrease slightly from 2.1 × 10^8^ to 1.8 × 10^8^ s^–1^ with increasing structural curvature. TD-DFT calculations at the CAM-B3LYP/6-31G(d) level indicated that the oscillator strength of the S_0_–S_1_ transitions also decreases in the same order (**3**: 0.96 > **5c**: 0.78 > **5b**: 0.70 > **5a**: 0.67) (Figure S46), which is in agreement with their extinction coefficients. These results can be explained by the decreased HOMO–LUMO overlap with increasing curvature. The increase in structural curvature also decreases the nonradiative decay rates, *k*_nr_, from 0.67 × 10^8^ to 0.24 × 10^8^ s^–1^. Transition-absorption-spectroscopy measurements indicated that the photoexcitation of **5b** and **5c** affords long-lived species with lifetimes of 86 and 125 μs, which were assigned to the triplet excited states (Figure S47). The intersystem crossing (ISC) quantum yield values, Φ_ISC_, of **5b** and **5c** are 0.1 and 0.2%, respectively (Figure S48). These Φ_ISC_ values are greater than the reported triplet yield of *N,N′*-bis(2,5-di-*tert*-butylphenyl)-substituted PBI **3‴** (Φ_ISC_ < 10^–4^),^58^ indicating that the molecular bending promotes ISC.^59^ However, the Φ_ISC_ values are in general very low, indicating that the nonradiative deactivation of the bent PBIs **5a**–**5c** mainly proceeds through internal conversion (IC). Using the reported fluorescence yield and decay of PBI **3‴**, its IC rate constant was estimated to be *k*_nr_ = 3 × 10^6^ s^–1^, which is approximately one-tenth of those of **5a**–**5c**. TD-DFT calculations of **5a**–**5c** also indicated the charge-transfer (CT) character between the core chromophores and the aryl units as the second excited state (S_2_) in the S_0_-optimized geometries (Figure S46). Thus, the disordered motion in the alkyl linker is anticipated to cause a vibronic admixture between S_1_ and S_2_ to promote the IC processes to the S_0_ states via CT character. Indeed, the Φ_F_ values in toluene (**3″**: 87%; **5a**: 90%; **5b**: 86%; **5c**:87%) are larger than those in CHCl_3_. Furthermore, the fact that the *k*_nr_ value of **5c** is greater than those of **5a** and **5b** strongly supports this hypothesis, because the flexibility of the alkyl chain linker increases with increasing length, allowing the orbital hybridization between HOMO and HOMO–1 as shown in Figure S46. In contrast, shorter linkages restrict the aforementioned orbital overlap due to the more rigid geometry. However, the *k*_nr_ value in **5a** is greater than that in **3‴**, indicating that the alkyl linkages essentially induce vibronic coupling for the nonradiative processes.

## Conclusions

We have described the synthesis and properties of bent PBI derivatives **5a**–**5c**, which feature an internal alkyl chain tethering the two *N*-aryl substituents. These PBIphanes were synthesized via sulfur-extrusion reactions from corresponding disulfoxide precursors **4a**–**4c**. X-ray diffraction analysis of **5a**–**5c** demonstrated that their degree of curvature increases with decreasing length of the alkyl tether. The strain energy was evaluated to be as high as 22.9 kcal mol^–1^ (**5a**) based on homodesmotic reactions. The ^1^H NMR analysis of **5a**–**5c** indicated that the aromatic proton signals were shifted upfield by approximately 0.1 ppm with increasing molecular curvature. This shift is due to the change in orientation toward the neighboring carbonyl group or aromatic ring rather than a change in aromaticity. Electrochemical measurements of **5a**–**5c** indicated that increasing the molecular curvature in PBI has a negligible effect on electron affinity. In sharp contrast, the increased curvature leads to a red shift of the absorption and emission spectra (by ∼20 nm), while the high fluorescence quantum yields (up to 88%) are maintained. This behavior contrasts with the nonemissive features of previously reported contorted π-extended PBI derivatives **6**. Detailed photophysical measurements of **5a**–**5c** indicated that the nonradiative deactivation of the S_1_ state via IC is suppressed by the restricted dynamic motion associated with the CT character between the core chromophores and the *N*-aryl units. Our results demonstrate that the double-sulfur-extrusion reactions enable the construction of curved perylene structures and the effect that the structural curvature exerts on the structural, electrochemical, and photophysical properties of these PBIs. These findings can be expected to find applications in the design of advanced organic materials.

## Supporting Information Available

Experimental details and spectral data for all new compounds (PDF)
