# Supplementary material for: End-to-End Bent Perylene Bisimide Cyclophanes by Double Sulfur Extrusion
Source: J Am Chem Soc. 2024 May 30;146(23):16332–9. doi: 10.1021/jacs.4c05358 (PMC11177258; doi:10.1021/jacs.4c05358)
Supplement: Supplementary file 1 — ja4c05358_si_001.pdf [file ja4c05358_si_001.pdf]

Supporting Information

End-to-End Bent Perylene Bisimide Cyclophanes by Double Sulfur Extrusion

Yuki Tanaka,[1] Keita Tajima,[1] Ryota Kusumoto,[2] Yasuhiro Kobori,[2,3,4]* Norihito Fukui,[1,4]* and Hiroshi Shinokubo[1]*

[1]Department of Molecular and Macromolecular Chemistry, Graduate School of Engineering, Nagoya University, Furo-cho, Chikusa-ku, Nagoya, Aichi 464-8603, Japan

[2] Department of Chemistry, Graduate School of Science, Kobe University, 1-1, Rokkodai-cho, Nada-ku, Kobe 657-8501, Japan.

[3] Molecular Photoscience Research Center, Kobe University, 1-1, Rokkodai-cho, Nada-ku, Kobe 657-8501, Japan.

[4] CREST, JST, Honcho 4-1-8, Kawaguchi, Saitama 332-0012, Japan.

[5] PRESTO, Japan Science and Technology Agency (JST), Kawaguchi, Saitama 332-0012, Japan

E-mail: ykobori@kitty.kobe-u.ac.jp, fukui@chembio.nagoya-u.ac.jp, hshino@chembio.nagoya-u.ac.jp

Table of Contents

1. Instrumentation and materials 3

2. Experimental procedures and compound data 4

3. NMR spectra 13

4. Mass spectra 38

5. Crystal data 51

6. Electrochemical properties 53

7. Thermal analysis 55

8. DFT Calculations 56

9. Photophysical properties 74

10. References 77

1. Instrumentation and materials

^{1}H NMR (500 MHz) and ^{13}C NMR (126 MHz) spectra were recorded on a Bruker AVANCE III HD spectrometer. ^{1}H NMR (600 MHz) and ^{13}C NMR (151 MHz) spectra were recorded on a JEOL JNM-ECA600II spectrometer. Chemical shifts were reported as the delta scale in ppm relative to solvent residual signals of $CDCl_3$ (δ = 7.26 ppm), DMSO-d_6 (δ = 2.50 ppm), and 1,1,2,2-tetrachroloethane-d_2 (δ = 6.0 ppm) for ^{1}H NMR and $CDCl_3$ (δ = 77.16 ppm), DMSO-d_6 (δ = 39.52 ppm), and 1,1,2,2-tetrachroloethane-d_2 (δ = 74.00 ppm) for ^{13}C NMR. UV/vis/NIR absorption spectra were recorded on a Shimadzu UV-2550 or JASCP V 770 spectrometer. Emission spectra were recorded on a Jasco FP-8550 spectrometer and absolute fluorescence quantum yields were measured by the photon-counting method using an integration sphere. Thermogravimetric analyses were recorded on a NETZSCH STA Regulus. Preparative separations were performed by silica gel column chromatography (Wako gel® C-300 or C-400). High-resolution atmospheric pressure chemical ionization time-of-flight (APCI-TOF) mass spectra were taken on a Bruker micrOTOF instrument. X-ray data were obtained using a Rigaku CCD diffractometer (Saturn 724 with MicroMax-007) with Varimax Mo optics. Cyclic voltammograms were obtained under the following conditions: solvent: CH_2Cl_2, electrolyte: 0.1 M Bu_4NPF_6, working electrode: glassy carbon, counter electrode: Pt, reference electrode: $Ag/AgNO_3$, scan rate: 0.05 V/s. Photo-irradiation was conducted by the USHIO OPM2-252HQ lamp house equipped with a super-high pressure mercury lamp (250 W) and an ASAHI SPECTRA LU0350 cut filter (cut range: λ< 350 nm) or L38 cut filter (cut range: λ< 380 nm).

All calculations were carried out using the Gaussian 16 software package.[1] Initial geometries of **5a**, **5b**, and **5c** were obtained from the X-ray crystal structure. Density functional theory (DFT) calculations were conducted with the restricted B3LYP[2] level, employing basis sets 6-31G(d).

Dry DMF was purchased from Wako Pure Chemical Industries, Ltd. as a dehydrated grade. Compounds **9** and **12** were prepared according to the literature.[3,4] Unless otherwise noted, materials obtained from commercial suppliers were used without further purification.

2. Experimental procedures and compound data

2,6-Dimethylphenyl DNDTBI *S*-oxide 10

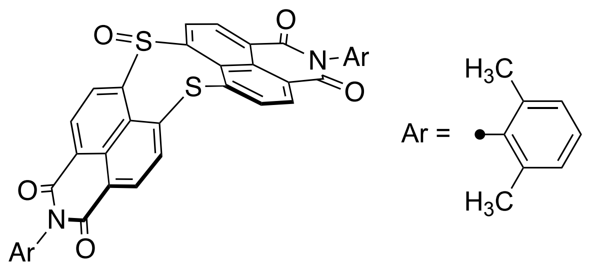

Compound **9** (66 mg, 0.10 mmol) and CH_2Cl_2 (5.0 mL) were placed in a round-bottom flask and cooled to 0 °C. To the flask, *m*-CPBA (containing ca. 30 wt% water, 29 mg, 0.12 mmol) was added, and the mixture was stirred for 1 h at 0 °C. The mixture was allowed to warm to room temperature and stirred for 1 h. After the reaction, the solution was separated by silica gel column chromatography (eluent: CH_2Cl_2/ethyl acetate = 30/1). After removal of the solvent in vacuo, recrystallization from CH_2Cl_2/CH_3OH afforded **10** (50 mg, 74 μmol, 74%) as a yellow solid.

^{1}H NMR (500 MHz, DMSO-d_6, 298 K): δ = 8.79 (d, J = 8.0 Hz, 2H), 8.76 (d, J = 8.0 Hz, 2H), 8.62 (d, J = 7.7 Hz, 2H), 8.58 (d, J = 7.7 Hz, 2H), 7.27 (t, J = 7.5 Hz, 2H), 7.22 (d, J = 7.2 Hz, 2H), 7.17 (d, J = 7.3 Hz, 2H), 2.02 (s, 6H), 1.88 (s, 6H) ppm; ^{13}C NMR (126 MHz, DMSO-d_6, 298 K): δ = 161.9, 161.8, 153.4, 138.0, 137.2, 135.42, 135.37, 133.5, 131.9, 130.2, 128.6, 128.2, 124.25, 124.19, 123.6, 17.3, 17.2 ppm (Three signals due to sp^2 carbon are missing due to overlapping.); HRMS (APCI): $[M+H]^+$ Calcd for $C_{40}H_{27}N_2O_5S_2$ 679.1356; Found 679.1332.

2,6-Dimethylphenyl DNDTBI *S*,*S*'-dioxide 11

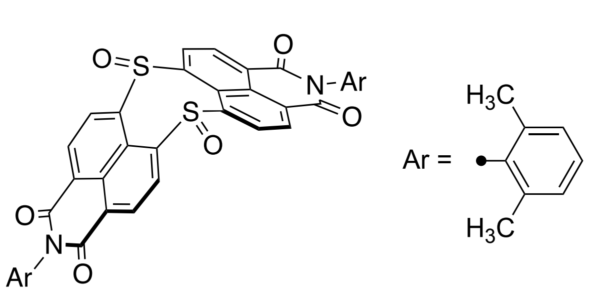

Compound **9** (66 mg, 0.10 mmol), $Na_2WO_4 \cdot 2H_2O$ (6.6 mg, 20 μmol), phenylphosphonic acid (3.2 mg, 20 μmol), $MeN(Oct)_3 \cdot HSO_4$ (9.3 mg, 20 μmol), toluene (2.5 mL), and H_2O_2 aq. (ca. 30

wt%, 1.0 mL, ca. 9 mmol) were placed in a round-bottom flask. The mixture was stirred under light-shielded conditions for 16 h at 80 °C. After the reaction, the mixture was cooled to room temperature and extracted with water and CH_2Cl_2. The organic layer was dried over Na_2SO_4. After removal of the solvent in vacuo, recrystallization from CH_2Cl_2/CH_3OH afforded **11** (50 mg, 73 μmol, 73%) as a yellow solid.

^{1}H NMR (500 MHz, DMSO-d_6, 298 K): δ = 8.78 (d, J = 8.0 Hz, 4H), 8.65 (d, J = 8.1 Hz, 4H), 7.27 (t, J = 7.5 Hz, 2H), 7.21 (d, J = 7.2 Hz, 2H), 7.17 (d, J = 7.3 Hz, 2H), 2.02 (s, 6H), 1.88 (s, 6H) ppm; ^{13}C NMR (126 MHz, DMSO-d_6, 298 K): δ = 161.7, 151.2, 135.4 (overlap), 133.4, 132.0, 129.8, 128.6 (overlap), 128.2, 124.8, 122.6, 122.1, 17.4, 17.3 ppm; HRMS (APCI): $[M+H]^+$ Calcd for $C_{40}H_{27}N_2O_6S_2$ 695.1305; Found 695.1294.

***o*-Anisyl DNDTBI 13**

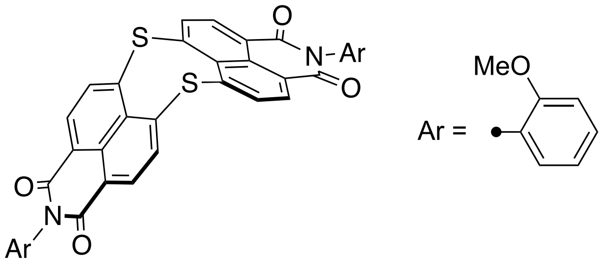

Compound **12** (1.8 g, 4.9 mmol) and DMF (0.40 L) were placed in a round-bottom flask. To the flask, Na_2S (0.70 g, 9.0 mmol) was added. The mixture was stirred for 3 h at room temperature. After the reaction was completed, 1 M HCl aq. was added. After removal of the solvent in vacuo, the residue was collected by filtration. The solid was washed with water, methanol, and Et_2O, affording a brown solid (1.2 g). The obtained brown solid (1.2 g), propionic acid (14 mL), *N*-methylpyrrolidone (NMP, 14 mL), and *o*-anisidine (2.8 mL, 25 mmol) were placed in a round-bottom flask, and the mixture was refluxed for 3 h at 170 °C. After the reaction mixture was cooled to room temperature, water was added to the mixture. The precipitate was filtered and washed with CH_3OH and Et_2O. The solid was dissolved in CH_2Cl_2, and insoluble yellow solids were removed by filtration. The filtrate was purified by silica gel column chromatography (eluent: CH_2Cl_2) to afford **13** (0.20 g, 0.30 mmol, 12%) as a yellow solid. Compound **13** was obtained as the mixture of the rotational isomers due to the *o*-anisyl groups.

^{1}H NMR (500 MHz, DMSO-d_6, 298 K): δ = 8.55–8.45 (m, 8H), 7.49–7.42 (m, 2H), 7.38–7.32 (m, 1H), 7.26–7.13 (m, 3H), 7.13–7.02 (m, 2H), 3.74–3.57 (m, 6H) ppm; HRMS (APCI): $[M+H]^+$

Calcd for $C_{38}H_{23}N_2O_6S_2$ 667.0992; Found 667.0975.

o-Hydroxyphenyl DNDTBI 14

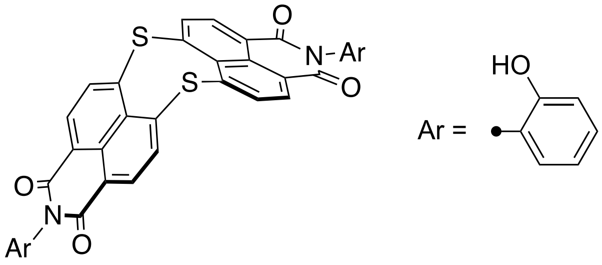

Compound **8** (0.83 g, 1.2 mmol) was placed in a round-bottom flask under argon. To the flask, CH_2Cl_2 (0.11 L, dried) was added, and the solution was cooled to 0 °C. BBr_3 (ca.1 M CH_2Cl_2 solution, 15 mL, 15 mmol) was added to the flask under argon. The solution was allowed to warm to room temperature and stirred for 1 h. After the reaction, excess NH_3 aqueous was added. The precipitate was filtered and washed with water and CH_3OH to afford **14** (0.74 g, 1.2 mmol, 93%) as an orange solid. Compound **14** was obtained as the mixture of the rotational isomers due to the *o*-hydroxyphenyl groups.

^{1}H NMR (500 MHz, DMSO-d_6, 298 K): δ = 8.55–8.45 (m, 8H), 7.30–6.80 (m, 8H) ppm; HRMS (APCI): $[M+H]^+$ Calcd for $C_{36}H_{19}N_2O_6S_2$ 639.0679; Found 639.0671.

C5-tethered DNDTIBI 15a

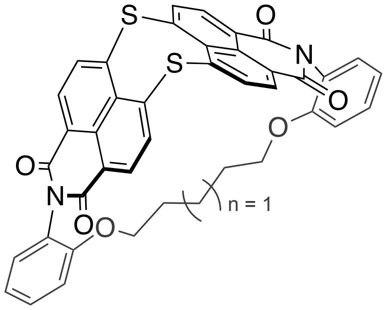

Compound **14** (0.59 g, 0.93 mmol) and K_2CO_3 (0.32 g, 2.3 mmol) were placed in a round-bottom flask under argon. To the flask, DMF (0.75 L, dried) and 1,5-dibromopentane (0.15 mL, 1.1 mmol) were added under argon, and the solution was stirred for 3 days at 40 °C. After removal of the solvent in vacuo, the residue was extracted with CH_2Cl_2 and washed with water. The

organic layer was dried over Na_2SO_4. After removal of the solvent in vacuo, the residue was separated by silica gel column chromatography (eluent: CH_2Cl_2/ethyl acetate = 50/1). After removal of the solvent in vacuo, recrystallization from CH_2Cl_2/MeOH afforded **15a** (0.11 g, 0.15 mmol, 17%) as an orange solid.

^{1}H NMR (500 MHz, 1,1,2,2-tetrachroloethane-*d*$_2$, 298 K): δ = 8.48 (d, *J* = 7.7 Hz, 4H), 8.35 (d, *J* = 7.7 Hz, 4H), 7.45–7.41 (m, 4H), 7.14 (t, *J* = 7.5 Hz, 2H), 6.87 (d, *J* = 7.5 Hz, 2H), 3.54 (br, 4H), 0.64 (br, 6H) ppm; ^{13}C NMR (126 MHz, 1,1,2,2-tetrachroloethane-*d*$_2$, 298 K): δ = 163.8, 152.7, 142.3, 137.6, 132.4, 131.5, 131.0, 130.5, 130.3, 125.1, 124.0, 120.9, 112.5, 67.0, 29.2, 19.8 ppm; HRMS (APCI): $[M+H]^+$ Calcd for $C_{41}H_{27}N_2O_6S_2$ 707.1305; Found 707.1287.

C6-tethered DNDTIBI 15b

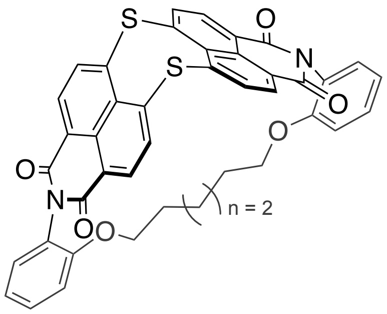

Compound **14** (0.48 g, 0.76 mmol) and K_2CO_3 (0.26 g, 1.9 mmol) were placed in a round-bottom flask under argon. To the flask, DMF (250 mL, dried) and 1,6-dibromohexane (0.14 mL, 0.91 mmol) were added. The solution was stirred for 3 days at 40 °C. After removal of the solvent in vacuo, the residue was extracted with CH_2Cl_2 and washed with water. The organic layer was dried over Na_2SO_4. After removal of the solvent in vacuo, the residue was separated by silica gel column chromatography (eluent: CH_2Cl_2/ethyl acetate = 50/1). After removal of the solvent in vacuo, recrystallization from CH_2Cl_2/MeOH afforded **15b** (0.13 g, 0.18 mmol, 24%) as an orange solid.

^{1}H NMR (500 MHz, 1,1,2,2-tetrachroloethane-*d*$_2$, 298 K): δ = 8.49 (d, *J* = 7.7 Hz, 4H), 8.35 (d, *J* = 7.7 Hz, 4H), 7.46 (t, *J* = 8.0 Hz, 2H), 7.35 (dd, *J* = 7.8 Hz, 1.3 Hz, 2H), 7.14 (t, *J* = 8.0 Hz, 2H), 7.01 (d, *J* = 8.0 Hz, 2H), 3.69 (t, *J* = 6.3 Hz, 4H), 1.06 (br, 4H), 0.70 (br, 4H) ppm; ^{13}C NMR (126 MHz, 1,1,2,2-tetrachroloethane-*d*$_2$, 298 K): δ = 163.2, 153.8, 142.1, 137.6, 132.4, 131.6, 131.0, 130.5, 130.2, 124.3, 124.0, 121.2, 113.8, 69.3, 28.7, 25.6 ppm; HRMS (APCI): $[M+H]^+$ Calcd for $C_{42}H_{29}N_2O_6S_2$ 721.1462; Found 721.1445.

C7-tethered DNDTIBI 15c

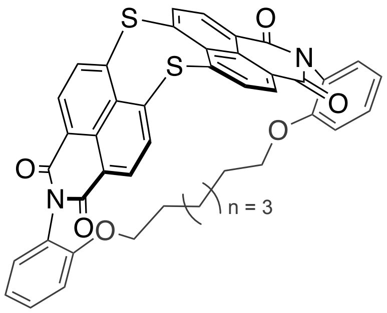

Compound **14** (0.16 g, 0.25 mmol) and K_2CO_3 (84 mg, 0.61 mmol) were placed in a round-bottom flask under argon. To the flask, DMF (200 mL, dried) and 1,7-dibromoheptane (47 µL, 0.28 mmol) were added. The solution was stirred for 4 days at 40 °C. After removal of the solvent in vacuo, the residue was extracted with water and CH_2Cl_2. The organic layer was dried over Na_2SO_4. After removal of the solvent in vacuo, the residue was separated by silica gel column chromatography (eluent: CH_2Cl_2/ethyl acetate = 50/1). After removal of the solvent in vacuo, recrystallization from CH_2Cl_2/hexane afforded **15c** (65 mg, 89 µmol, 36%) as an orange solid.

^{1}H NMR (500 MHz, 1,1,2,2-tetrachroloethane-d_2, 298 K): δ = 8.51 (d, J = 7.7 Hz, 4H), 8.40 (d, J = 7.7 Hz, 4H), 7.46 (t, J = 7.2 Hz, 2H), 7.41 (dd, J = 7.2 Hz, 1.2 Hz, 2H), 7.15 (t, J = 7.2 Hz, 2H), 6.95 (d, J = 7.2 Hz, 2H), 3.68 (t, J = 4.8 Hz, 4H), 1.01 (q, J = 4.8 Hz, 4H), 0.34 (q, J = 4.8 Hz, 4H), –0.39 (q, J = 4.8 Hz, 4H) ppm; ^{13}C NMR (126 MHz, 1,1,2,2-tetrachroloethane-d_2, 298 K): δ = 163.6, 153.2, 141.6, 137.6, 132.5, 131.5, 130.8, 130.6, 130.1, 124.8, 124.2, 121.0, 113.1, 67.6, 28.6, 27.6, 22.7 ppm; HRMS (APCI): $[M+H]^+$ Calcd for $C_{43}H_{31}N_2O_6S_2$ 735.1618; Found 735.1587.

C5-tethered DNDTIBI *S,S'*-dioxide 4a

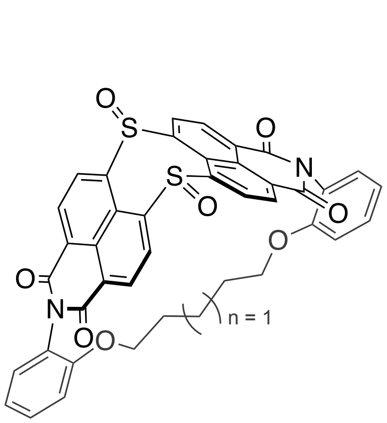

Compound **15a** (83 mg, 0.12 mmol), $Na_2WO_4 \cdot 2H_2O$ (39 mg, 0.12 mmol), phenylphosphonic acid (19 mg, 0.12 mmol), $MeN(Oct)_3 \cdot HSO_4$ (55 mg, 0.12 mmol), toluene (80 mL), and H_2O_2 aq. (ca. 30wt%, 10 mL, 8.8 mmol) were placed in a round-bottom flask. The mixture was stirred under light-shielded conditions for 23 h at 80 °C. The mixture was cooled to room temperature. After removal of the solvent in vacuo, the residue was extracted with CH_2Cl_2 and washed with water. The organic layer was dried over Na_2SO_4. After removal of the solvent in vacuo, the residue was separated by silica gel column chromatography (eluent: CH_2Cl_2/ethyl acetate = 10/1). After removal of the solvent in vacuo, the solid material was washed with CH_2Cl_2 (ca. 3 mL), affording **4a** (78 mg, 0.11 mmol, 90%) as a pink solid.

^{1}H NMR (600 MHz, 1,1,2,2-tetrachroloethane-d_2, 298 K): δ = 8.73 (d, J = 8.2 Hz, 4H), 8.65 (d, J = 8.2 Hz, 4H), 7.46 (td, J_1 = 7.7 Hz, J_2 = 1.2 Hz, 2H), 7.39 (dd, J_1 = 7.6 Hz, J_2 = 1.4 Hz, 2H), 7.15 (t, J = 7.8 Hz, 2H), 6.90 (d, J = 7.8 Hz, 2H), 3.59 (t, J = 6.9 Hz, 4H), 0.77 (quin, J = 6.9 Hz, 4H), 0.60 (quin, J = 6.9 Hz, 2H) ppm; ^{13}C NMR (150 MHz, 1,1,2,2-tetrachroloethane-d_2, 298 K): δ = 162.8, 152.8, 151.3, 132.3, 130.8, 130.1, 130.0, 125.4, 124.2, 122.0, 121.5, 121.0, 112.5, 66.4, 28.4, 19.3 ppm; HRMS (APCI): $[M+H]^+$ Calcd for $C_{41}H_{27}N_2O_8S_2$ 739.1203; Found 739.1196.

C6-tethered DNDTIBI *S*,*S*'-dioxide 4b

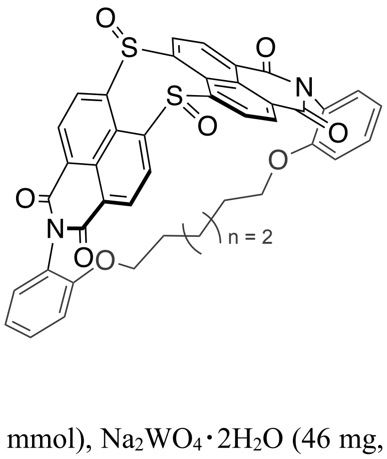

Compound **15b** (0.10 g, 0.14 mmol), $Na_2WO_4 \cdot 2H_2O$ (46 mg, 0.14 mmol), phenylphosphonic acid (22 mg, 0.14 mmol), $MeN(Oct)_3 \cdot HSO_4$ (65 mg, 0.14 mmol), toluene (95 mL), and H_2O_2 aq. (ca. 30wt%, 12 mL, 11 mmol) were placed in a round-bottom flask. The mixture was stirred under light-shielded conditions for 22 h at 80 °C. The mixture was cooled to room temperature. After removal of the solvent in vacuo, the residue was extracted with CH_2Cl_2 and washed with water. The organic layer was dried over Na_2SO_4. After removal of the solvent in vacuo, the residue was separated by silica gel column chromatography (eluent: CH_2Cl_2/ethyl acetate = 20/1). After

removal of the solvent in vacuo, recrystallization from CH_2Cl_2/MeOH afforded **4b** (64 mg, 85 μmol, 61%) as a pink solid.

^{1}H NMR (500 MHz, 1,1,2,2-tetrachroloethane-d_2, 298 K): δ = 8.75 (d, J = 7.9 Hz, 4H), 8.67 (d, J = 7.9 Hz, 4H), 7.48 (td, J_1 = 7.8 Hz, J_2 = 1.6 Hz, 2H), 7.31 (dd, J_1 = 7.8 Hz, J_2 = 1.6 Hz, 2H), 7.15 (td, J_1 = 7.8 Hz, J_2 = 1.6 Hz, 2H), 7.03 (d, J = 7.8 Hz, 2H), 3.72 (t, J = 6.4 Hz, 4H), 1.13 (br, 4H), 0.78 (br, 4H) ppm; ^{13}C NMR (126 MHz, 1,1,2,2-tetrachroloethane-d_2, 298 K): δ = 162.3, 153.8, 151.0, 132.4, 130.8, 130.0, 129.9, 125.4, 123.7, 122.2, 121.7, 121.2, 113.6, 69.1, 28.6, 25.4 ppm; HRMS (APCI): $[M+H]^+$ Calcd for $C_{42}H_{29}N_2O_8S_2$ 753.1360; Found 753.1356.

C7-tethered DNDTIBI *S,S'*-dioxide 4c

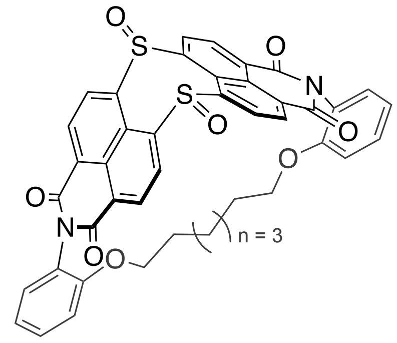

Compound **15c** (25 mg, 34 μmol), $Na_2WO_4\cdot 2H_2O$ (13 mg, 40 μmol), phenylphosphonic acid (5.0 mg, 32 μmol), $MeN(Oct)_3\cdot HSO_4$ (15 mg, 32 μmol), toluene (23 mL), and H_2O_2 aq. (ca. 30wt%, 4 mL, 2.8 mmol) were placed in a round-bottom flask. The mixture was stirred under light-shielded conditions for 23 h at 80 °C. After the reaction, the mixture was cooled to room temperature and extracted with water and CH_2Cl_2. The organic layer was dried over Na_2SO_4. After removal of the solvent in vacuo, the residue was separated by silica gel column chromatography (eluent: from CH_2Cl_2 to CH_2Cl_2/ethyl acetate = 25/1). After removal of the solvent in vacuo, recrystallization from CH_2Cl_2/hexane afforded **4c** (13 mg, 17 μmol, 49%) as a pink solid.

^{1}H NMR (500 MHz, $CDCl_3$, 298 K): δ = 8.85 (d, J = 8.0 Hz, 4H), 8.71 (d, J = 8.0 Hz, 4H), 7.42 (td, J_1 = 7.5 Hz, J_2 = 1.8 Hz, 2H), 7.31 (dd, J_1 = 7.5 Hz, J_2 = 1.8 Hz, 2H), 7.10 (t, J = 7.5 Hz, 2H), 6.95 (d, J = 7.5 Hz, 2H), 3.77 (t, J = 5.5 Hz, 4H), 1.30–1.10 (m, 4H), 0.64 (quin, J = 7.5 Hz, 4H), 0.29 (quin, J = 7.5 Hz, 2H) ppm; ^{13}C NMR (126 MHz, $CDCl_3$, 298 K): δ = 162.5, 153.9, 150.7, 132.2, 130.8, 130.3, 129.9, 126.0, 124.0, 122.6, 122.4, 121.2, 113.6, 68.6, 29.4, 29.0, 24.2 ppm; HRMS (APCI): $[M+H]^+$ Calcd for $C_{43}H_{31}N_2O_8S_2$ 767.1516; Found 767.1480.

C5-tethered PBIphane 5a

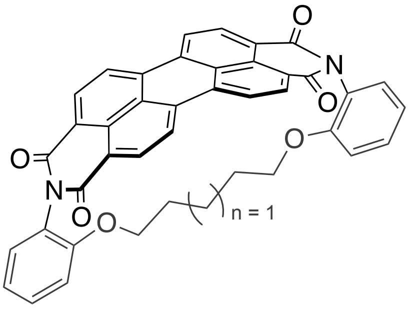

Compound **4a** (7.2 mg, 9.8 μmol) and CH_2Cl_2 (0.10 L) were placed in a round-bottom flask. The mixture was stirred under photo irradiation (λ > 350 nm) for 2 h at room temperature. The crude mixture was directly loaded on silica gel column pre-eluted with CH_2Cl_2. The mixture was purified by the subsequent elution with CH_2Cl_2/ethyl acetate (33/1 to 20/1). After removal of the solvent in vacuo, recrystallization from CH_2Cl_2/MeOH afforded **5a** (4.2 mg, 6.5 μmol, 67%) as a red solid.

^{1}H NMR (500 MHz, 1,1,2,2-tetrachroloethane-*d*$_2$, 298 K): δ = 8.64 (d, J = 8.5 Hz, 4H), 8.62 (d, J = 8.5 Hz, 4H), 7.72 (dd, J_1 = 7.5 Hz, J_2 = 1.5 Hz, 2H), 7.35 (td, J_1 = 7.5 Hz, J_2 = 1.5 Hz, 2H), 7.13 (t, J = 7.5 Hz, 2H), 6.93 (d, J = 7.5 Hz, 2H), 3.16 (t, J = 8.0 Hz, 4H), 0.37 (br, 2H), –0.53 (br, 4H) ppm; ^{13}C NMR (126 MHz, 1,1,2,2-tetrachroloethane-*d*$_2$, 298 K): δ = 166.7, 152.3, 135.3, 132.2, 132.0, 131.9, 130.4, 129.3, 127.5, 124.1 (overlap), 120.8, 111.7, 66.8, 29.6, 19.0 ppm; HRMS (APCI): [M+H]$^+$ Calcd for $C_{41}H_{27}N_2O_6$ 643.1864; Found 643.1881.

C6-tethered PBIphane 5b

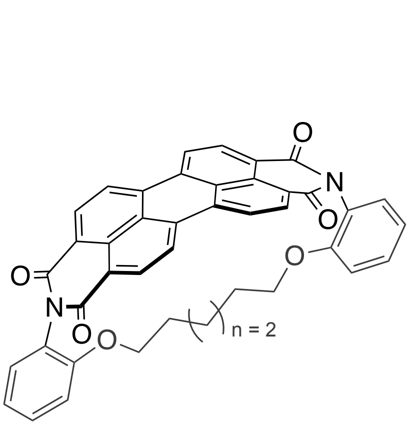

Compound **4b** (7.9 mg, 10 μmol) and CH_2Cl_2 (0.10 L) were placed in a round-bottom flask. The mixture was stirred under photo irradiation (λ > 350 nm) for 2 h at room temperature. The

crude mixture was directly loaded on silica gel column pre-eluted with CH_2Cl_2. The mixture was purified by the subsequent elution with CH_2Cl_2/ethyl acetate (50/1 to 30/1). After removal of the solvent in vacuo, recrystallization from CH_2Cl_2/MeOH afforded **5b** (4.9 mg, 7.5 μmol, 71%) as a reddish orange solid.

^{1}H NMR (500 MHz, 1,1,2,2-tetrachroloethane-d_2, 298 K): δ = 8.67 (d, J = 8.5 Hz, 4H), 8.65 (d, J = 8.5 Hz, 4H), 7.72 (d, J = 7.5 Hz, 2H), 7.39 (t, J = 7.5 Hz, 2H), 7.20 (t, J = 7.5 Hz, 2H), 6.85 (d, J = 7.5 Hz, 2H), 3.25 (t, J = 6.5 Hz, 4H), 0.26 (br, 4H), –0.08 (br, 4H) ppm; ^{13}C NMR (126 MHz, 1,1,2,2-tetrachroloethane-d_2, 298 K): δ = 165.9, 152.9, 135.0, 131.9, 131.5, 131.3, 130.3, 129.2, 127.0, 123.8, 123.6, 121.7, 115.1, 70.2, 29.6, 26.0 ppm; HRMS (APCI): $[M+H]^+$ Calcd for $C_{42}H_{29}N_2O_6$ 657.2020; Found 657.1994.

C7-tethered PBIphane 5c

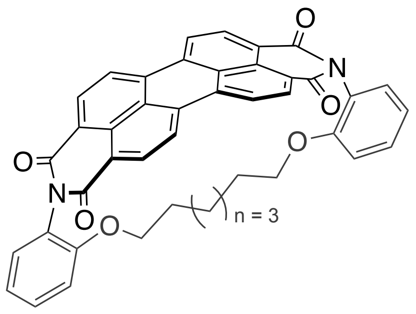

Compound **4c** (7.1 mg, 9.3 μmol) and CH_2Cl_2 (0.10 L) were placed in a round-bottom flask. The mixture was stirred under photo irradiation (λ > 350 nm) for 2 h at room temperature. The crude mixture was directly loaded on silica gel column pre-eluted with CH_2Cl_2. The mixture was purified by the subsequent elution with CH_2Cl_2/ethyl acetate (30/1 to 20/1). After removal of the solvent in vacuo, recrystallization from CH_2Cl_2/MeOH afforded **5c** (5.2 mg, 7.8 μmol, 84%) as an orange solid.

^{1}H NMR (600 MHz, 1,1,2,2-tetrachroloethane-d_2, 298 K): δ = 8.71 (d, J = 7.8 Hz, 4H), 8.69 (d, J = 7.8 Hz, 4H), 7.70 (dd, J_1 = 7.8 Hz, J_2 = 1.2 Hz, 2H), 7.42 (td, J_1 = 7.8 Hz, J_2 = 1.2 Hz, 2H), 7.21 (t, J = 7.8 Hz, 2H), 6.86 (d, J = 7.8 Hz, 2H), 3.35 (t, J = 7.2 Hz, 4H), 0.30–0.13 (m, 8H), –0.40 (quin, J = 7.2 Hz, 2H) ppm; ^{13}C NMR (150 MHz, 1,1,2,2-tetrachroloethane-d_2, 298 K): δ = 165.8, 152.6, 135.2, 132.1, 131.4, 131.1, 130.5, 128.9, 127.1, 124.0, 123.9, 121.7, 114.8, 69.3, 32.6, 31.6, 24.3 ppm; HRMS (APCI): $[M+H]^+$ Calcd for $C_{43}H_{31}N_2O_6$ 671.2177; Found 671.2152.

3. NMR spectra

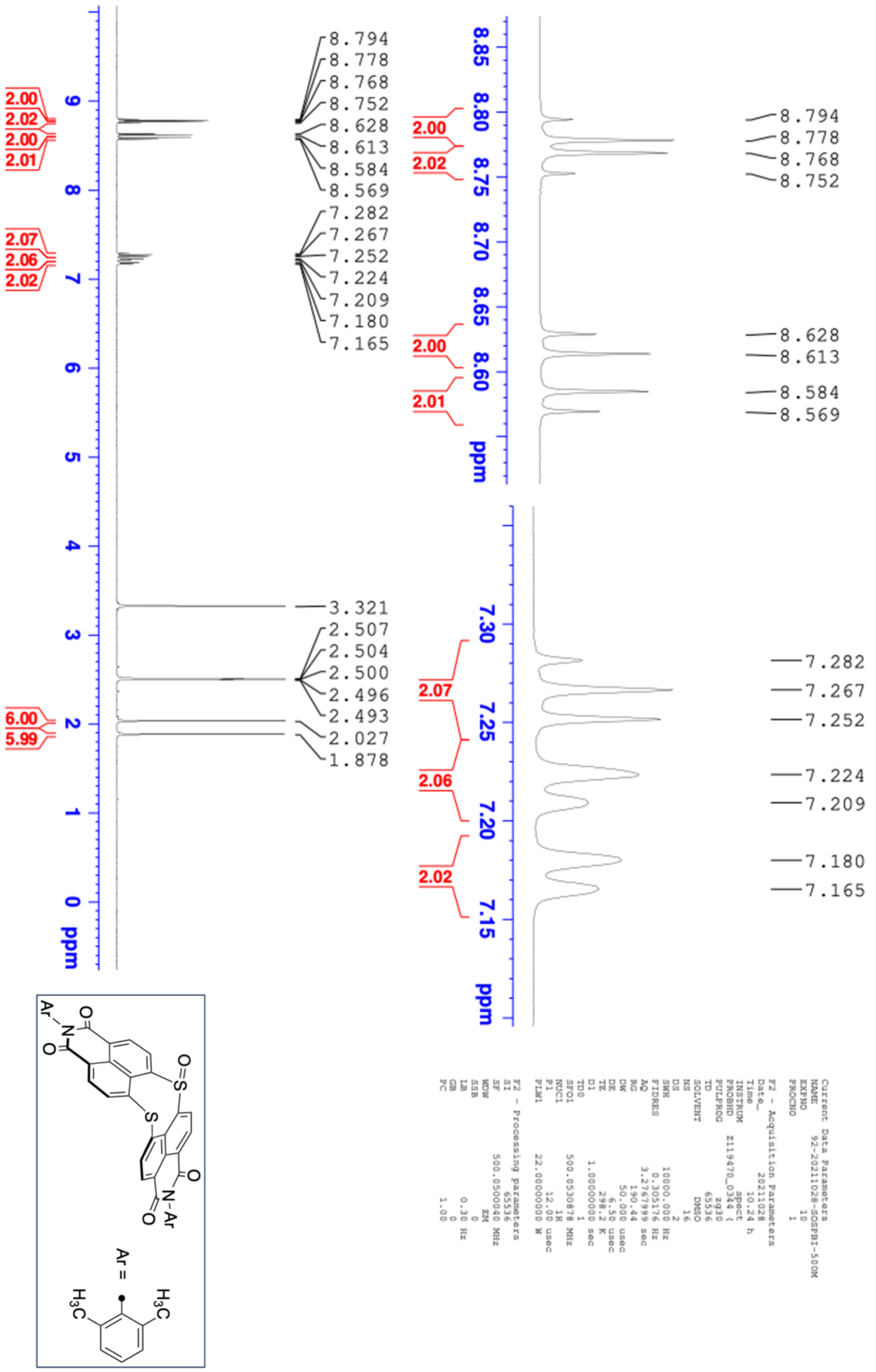

Figure S1. ^{1}H NMR spectrum of **10** in DMSO-d_6 at 25 °C.

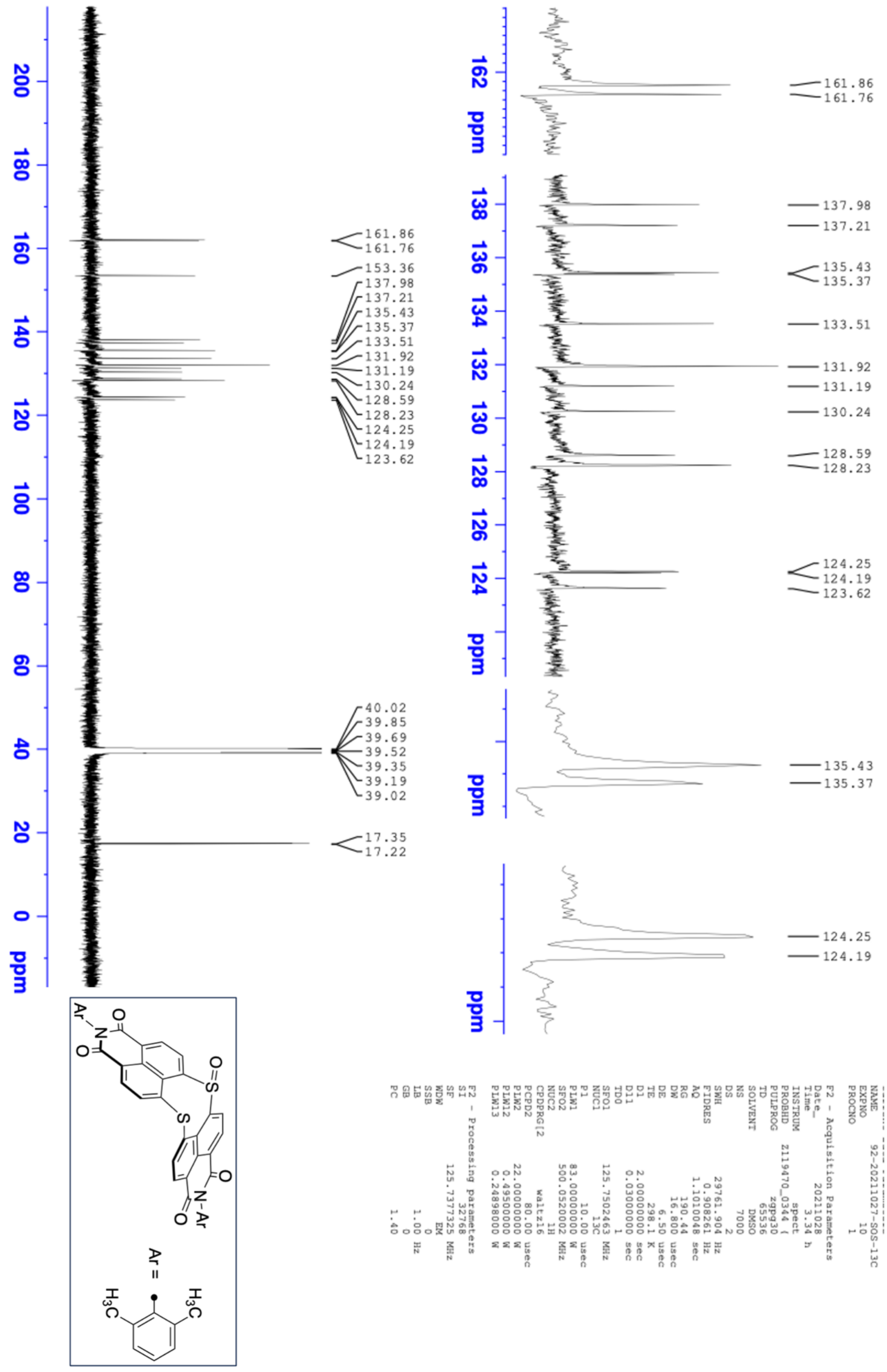

Figure S2. ^{13}C NMR spectrum of **10** in DMSO-d_6 at 25 °C.

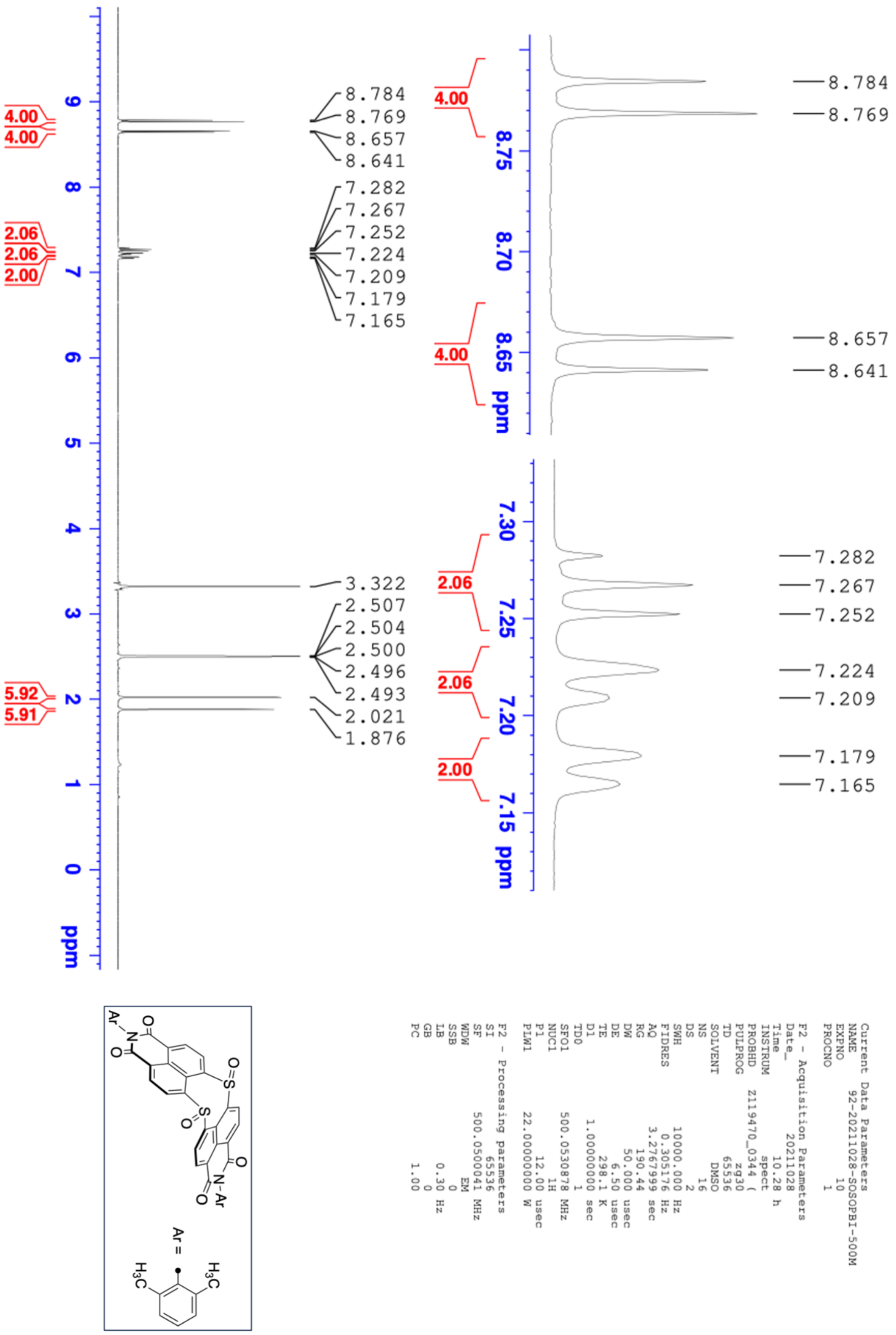

Figure S3. ^{1}H NMR spectrum of **11** in DMSO-d_6 at 25 °C.

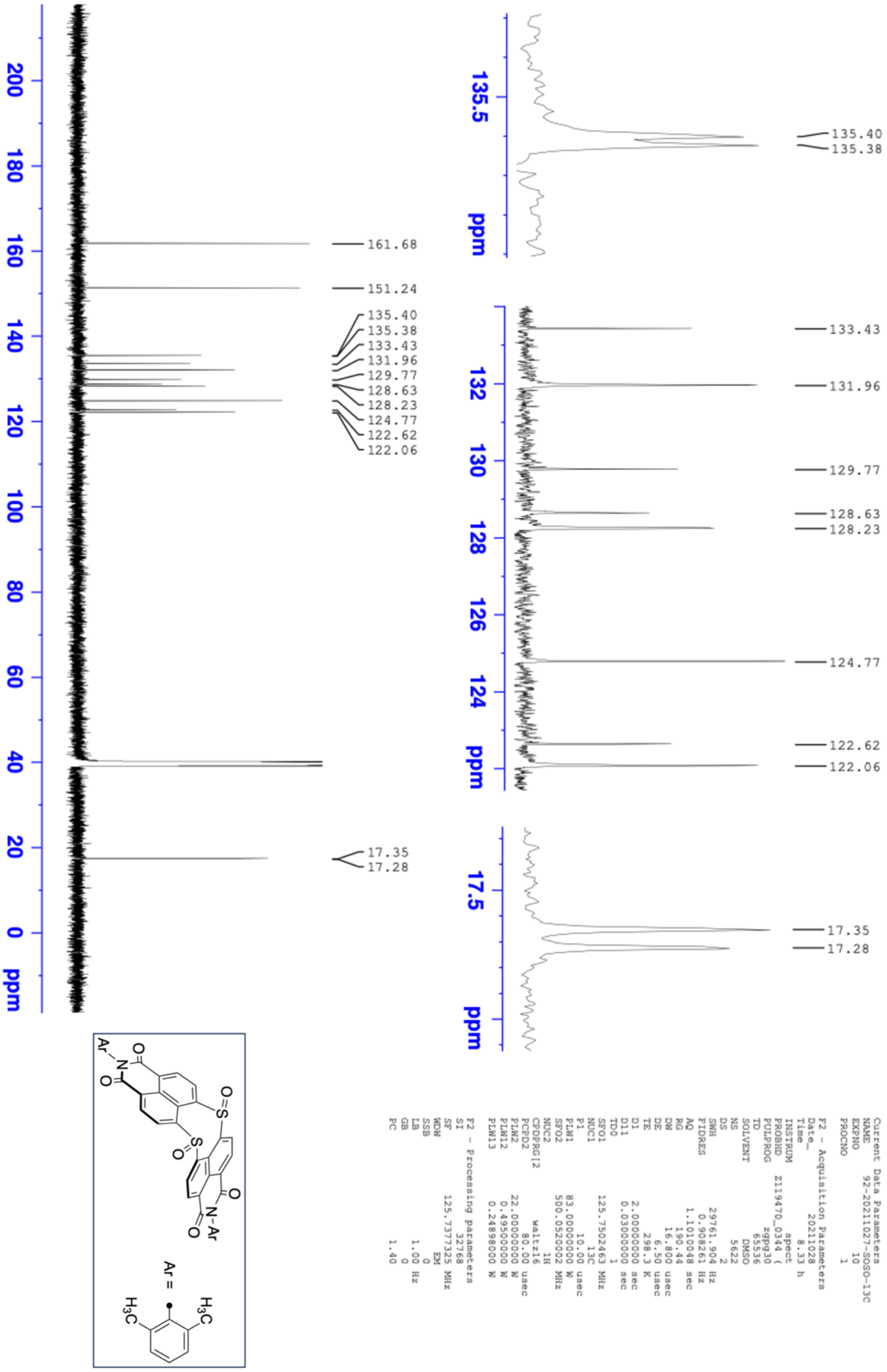

Figure S4. ^{13}C NMR spectrum of **11** in DMSO-d_6 at 25 °C.

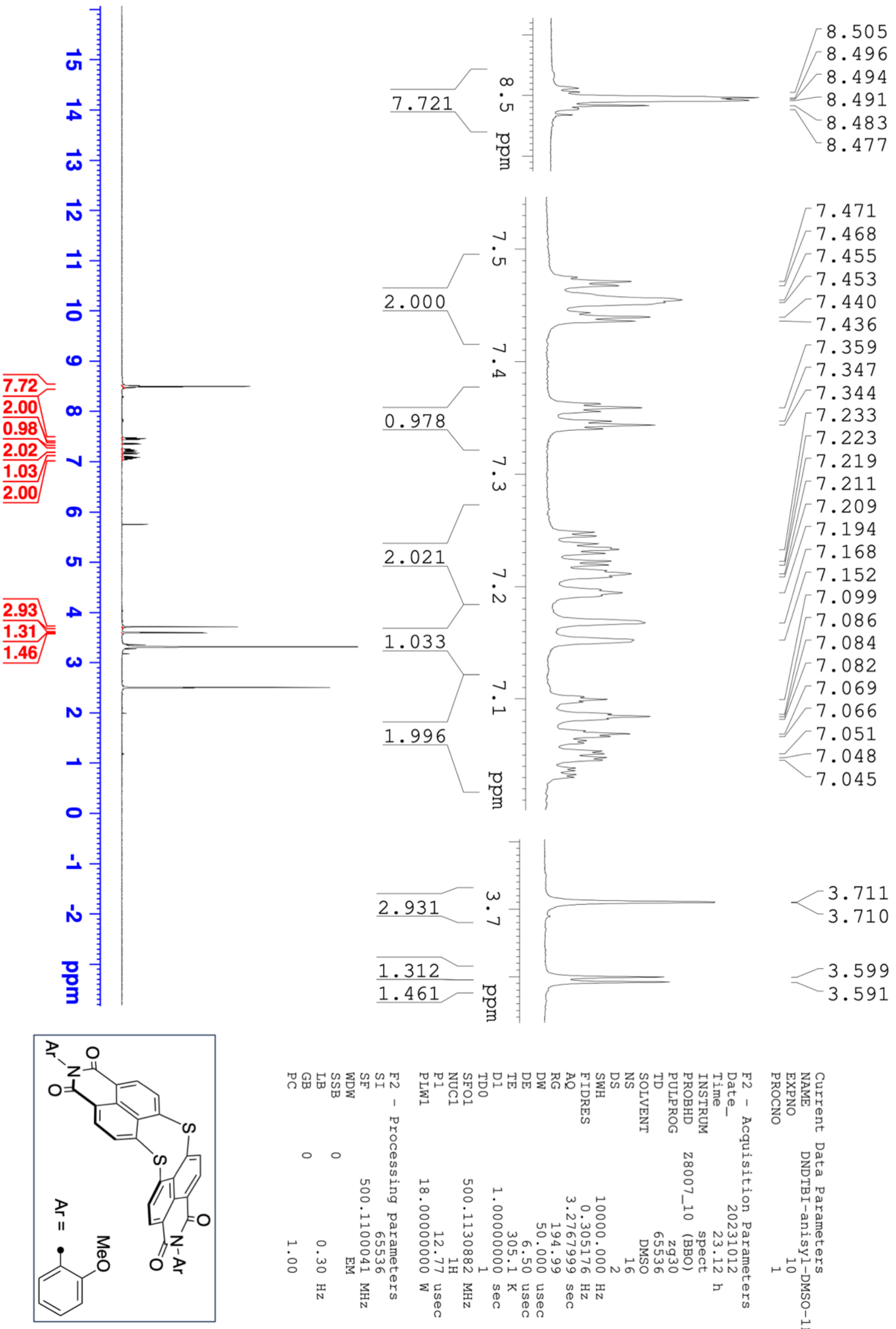

Figure S5. ^{1}H NMR spectrum of **13** in DMSO-d_6 at 25 °C.

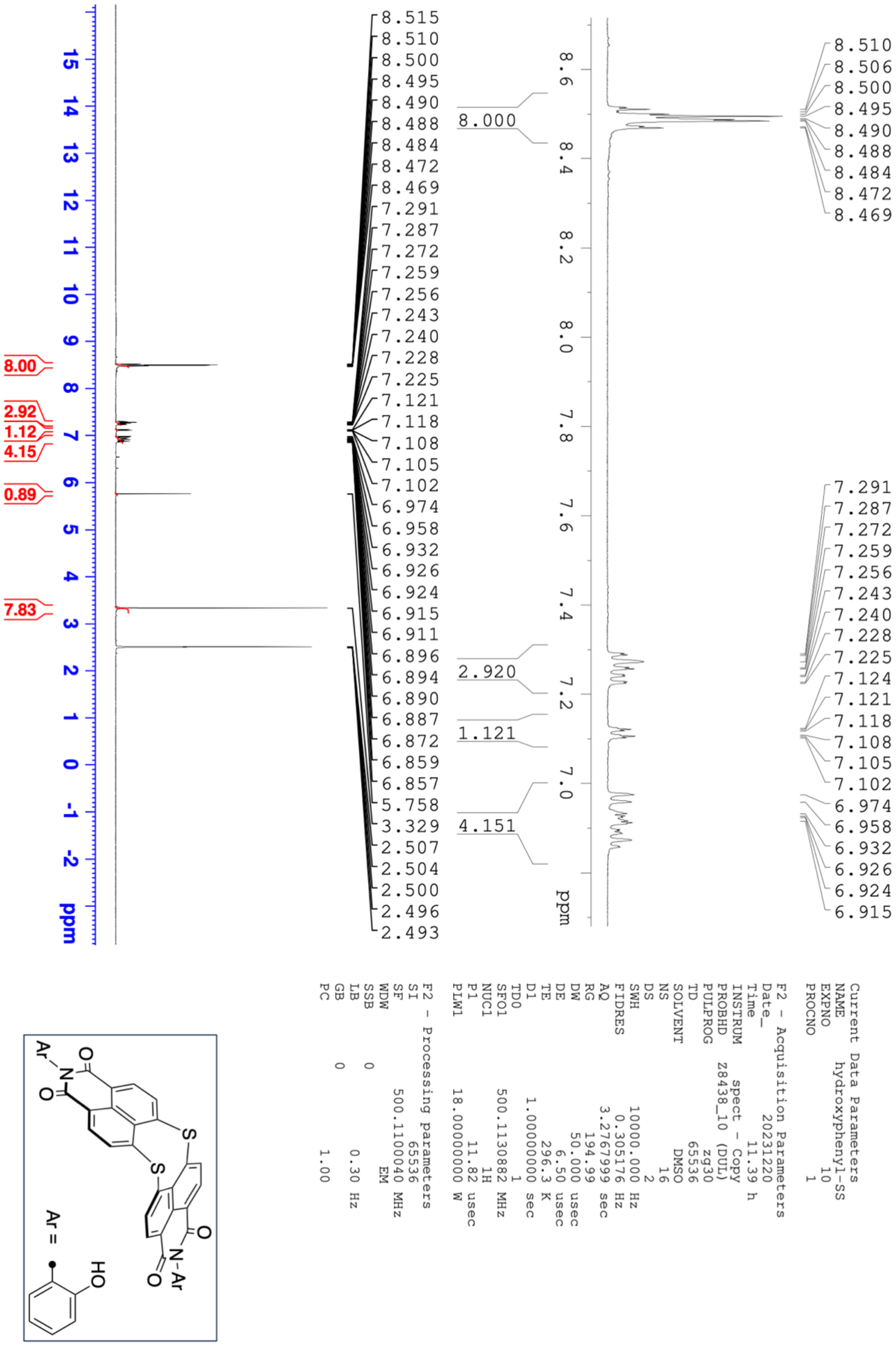

Figure S6. ^{1}H NMR spectrum of **14** in DMSO-d_6 at 25 °C.

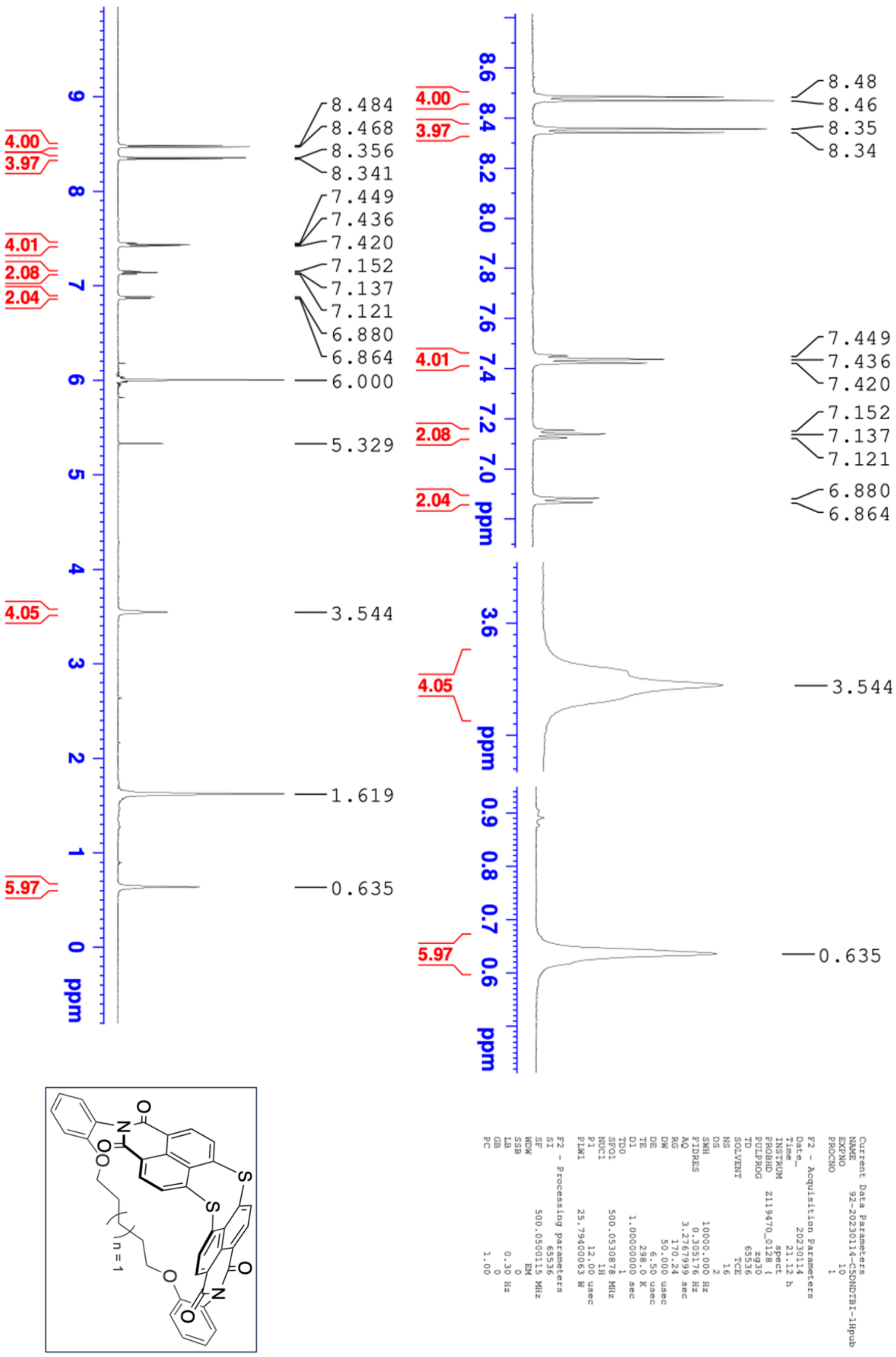

Figure S7. ^{1}H NMR spectrum of **15a** in 1,1,2,2-tetrachroloethane-d_2 at 25 °C.

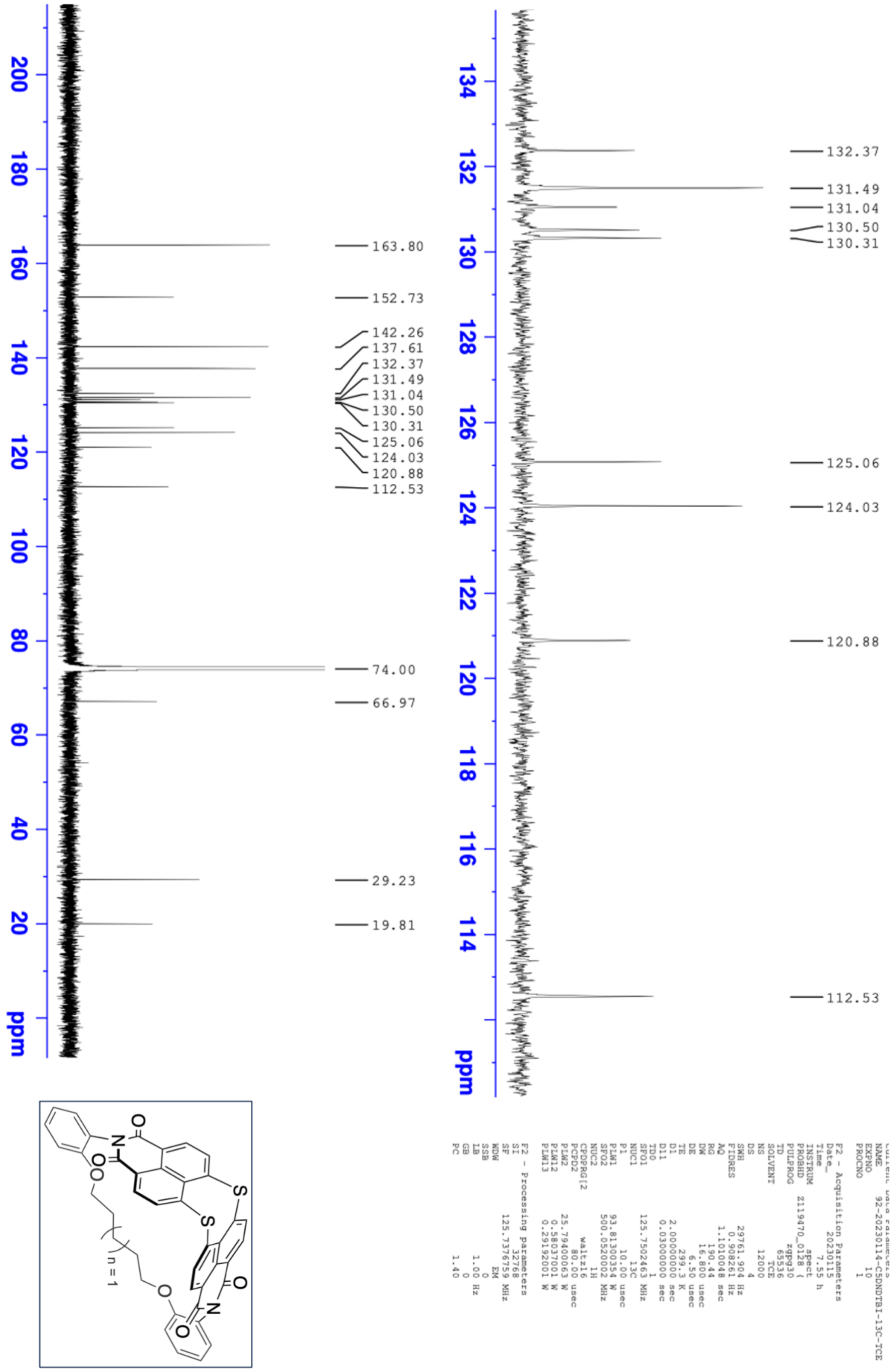

Figure S8. ^{13}C NMR spectrum of **15a** in 1,1,2,2-tetrachroloethane-d_2 at 25 °C.

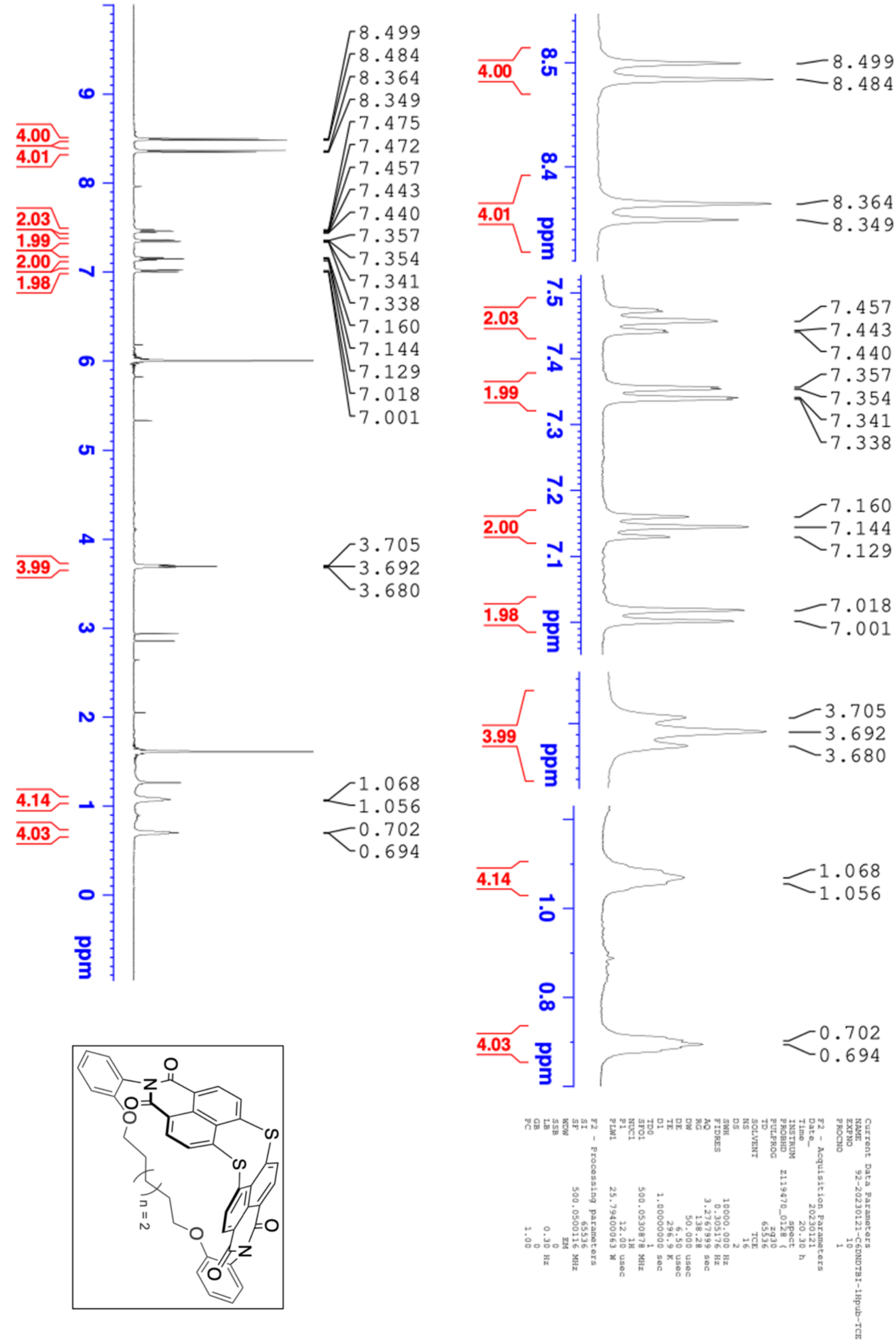

Figure S9. ^{1}H NMR spectrum of **15b** in 1,1,2,2-tetrachroloethane-d_2 at 25 °C.

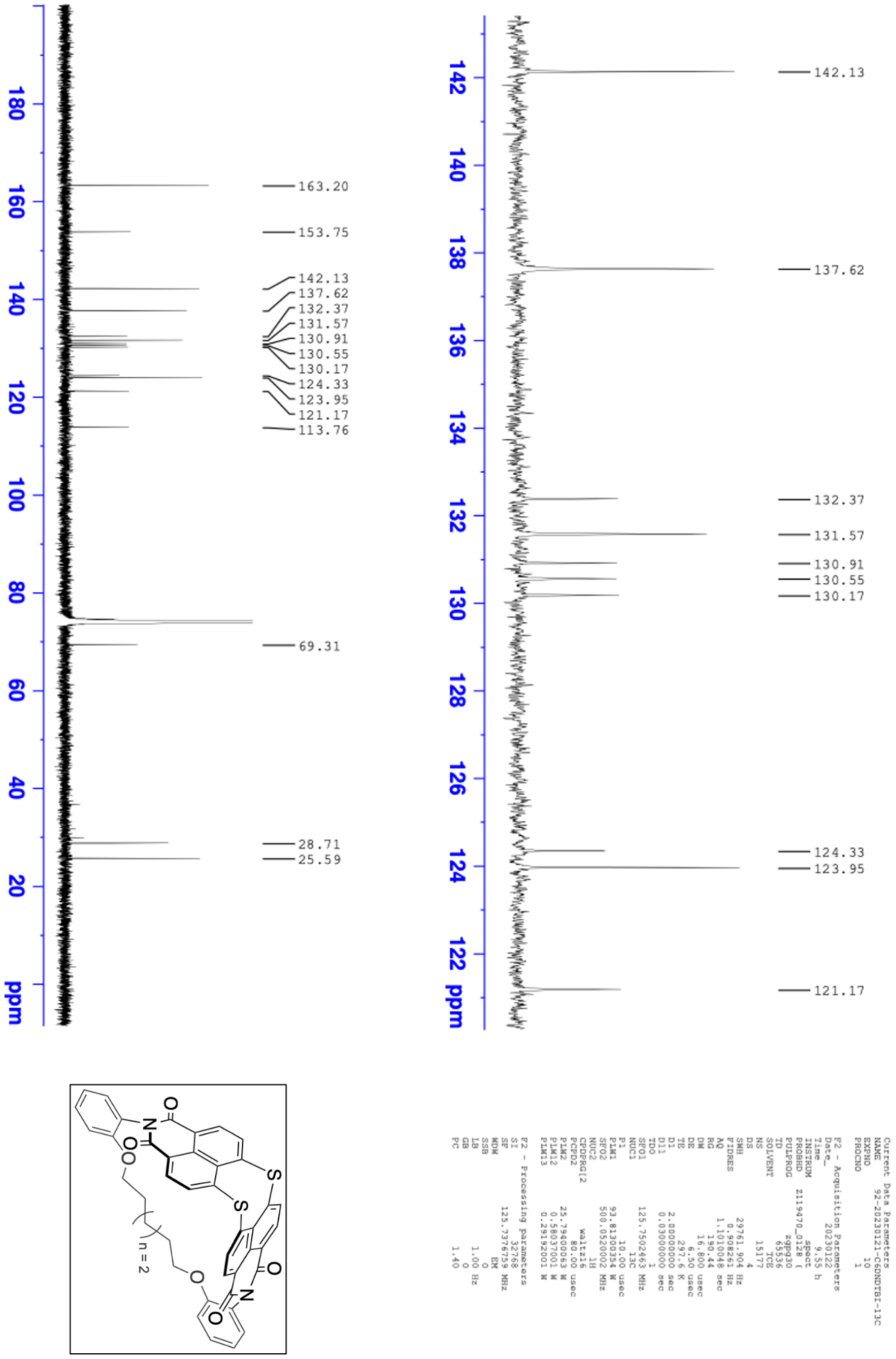

Figure S10. ^{13}C NMR spectrum of **15b** in 1,1,2,2-tetrachroloethane-d_2 at 25 °C.

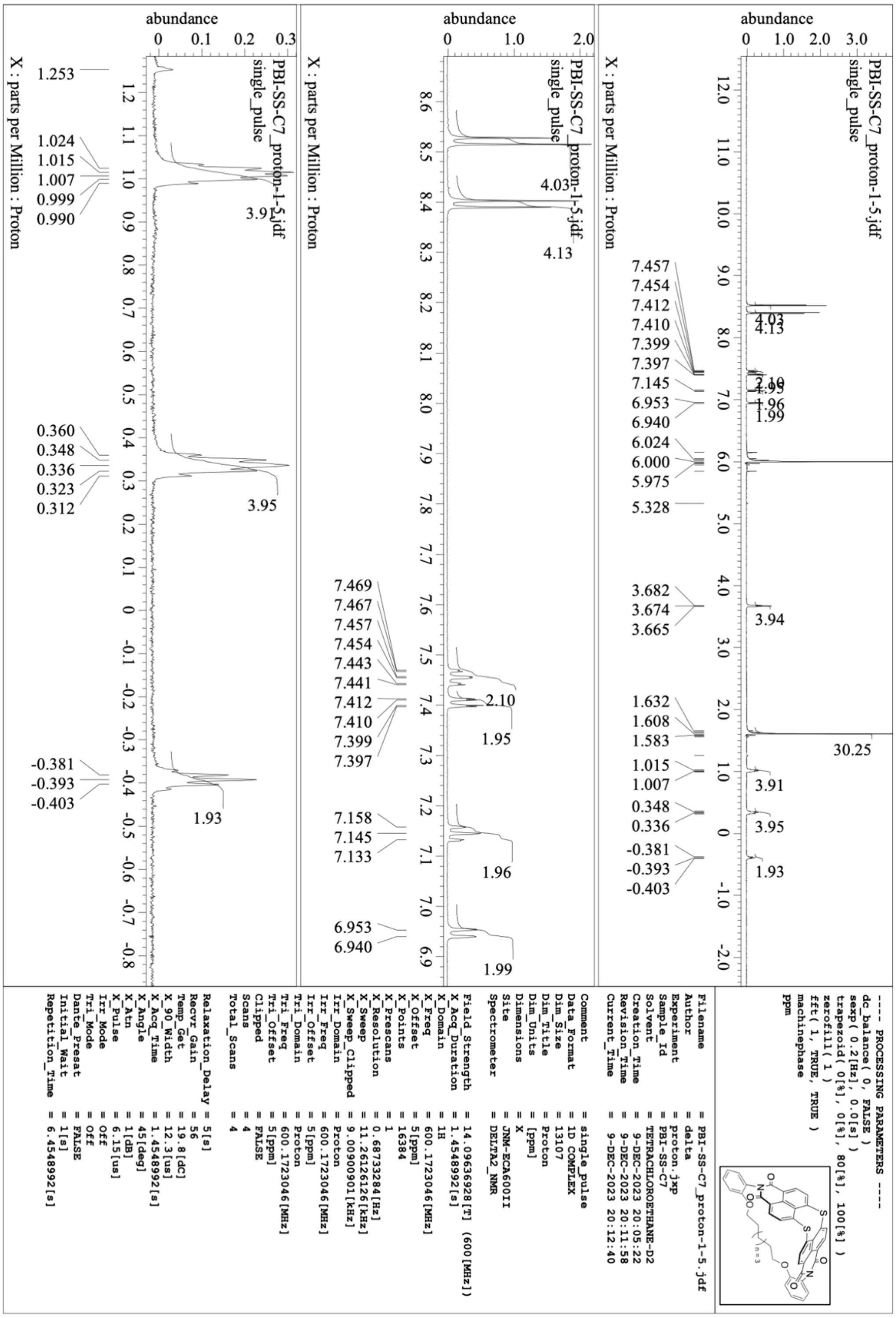

Figure S11. ^{1}H NMR spectrum of **15c** in 1,1,2,2-tetrachroloethane-d_2 at 25 °C.

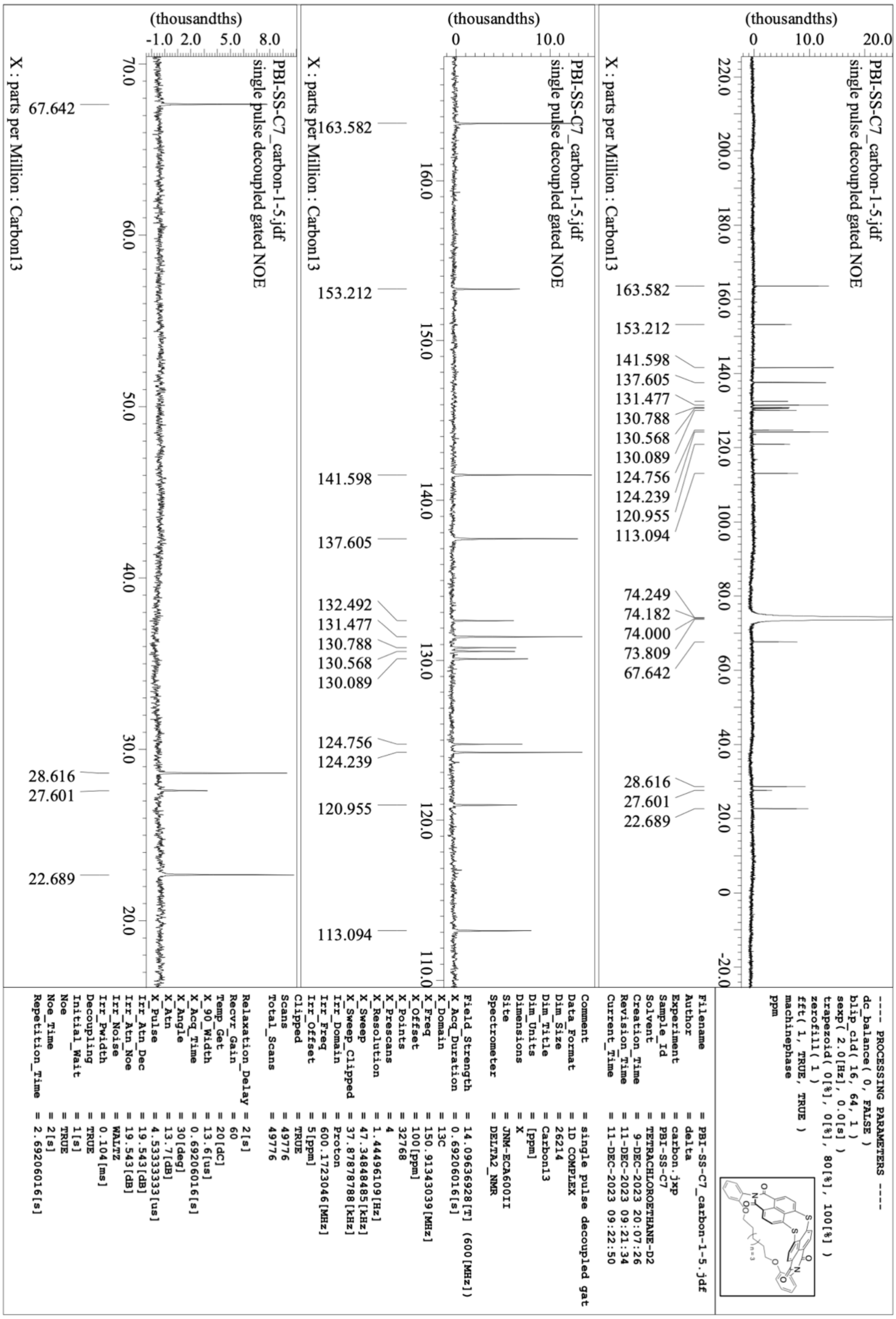

Figure S12. ^{13}C NMR spectrum of **15c** in 1,1,2,2-tetrachroloethane-d_2 at 25 °C.

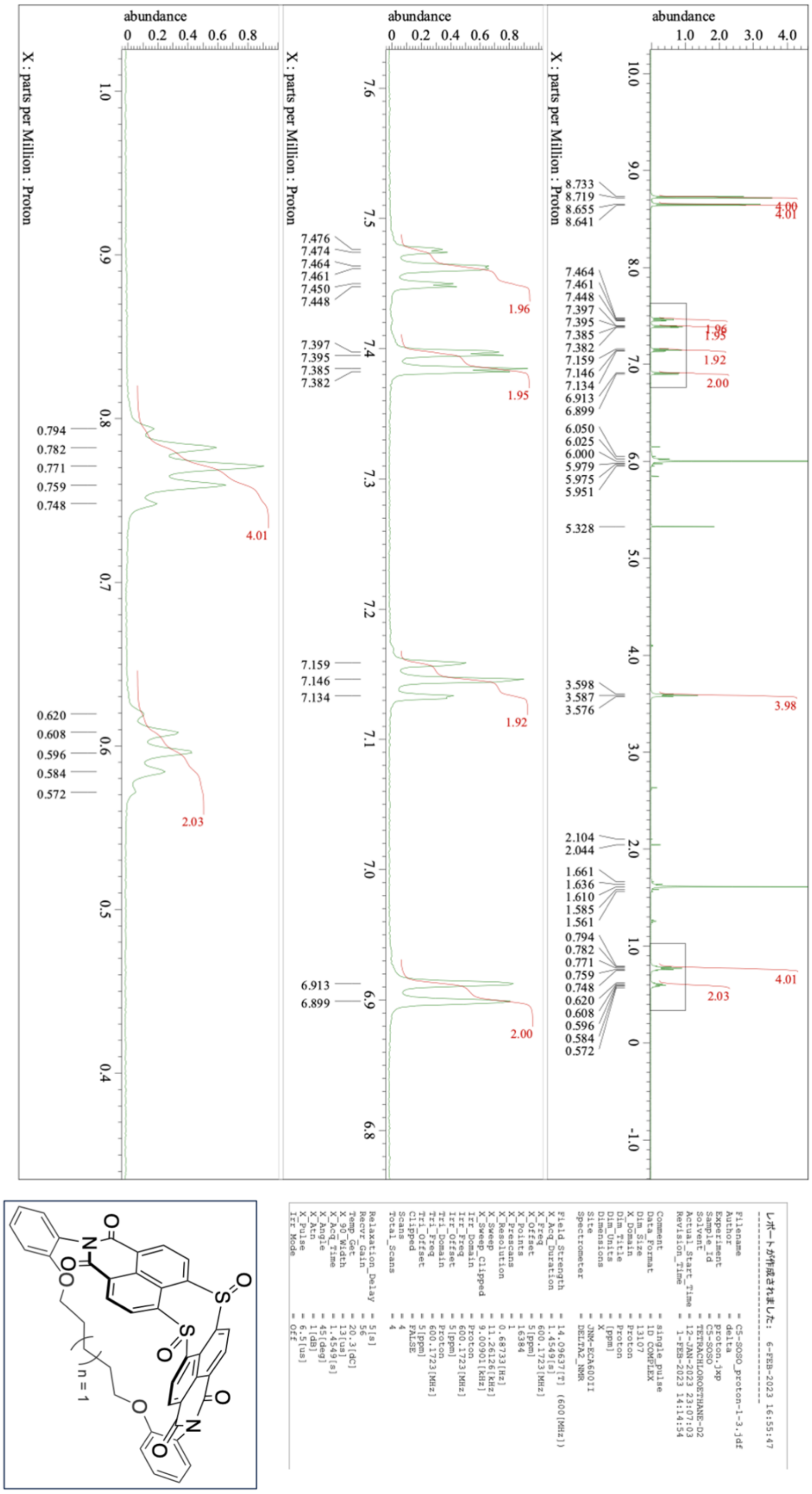

Figure S13. ^{1}H NMR spectrum of **4a** in 1,1,2,2-tetrachroloethane-d_2 at 25 °C.

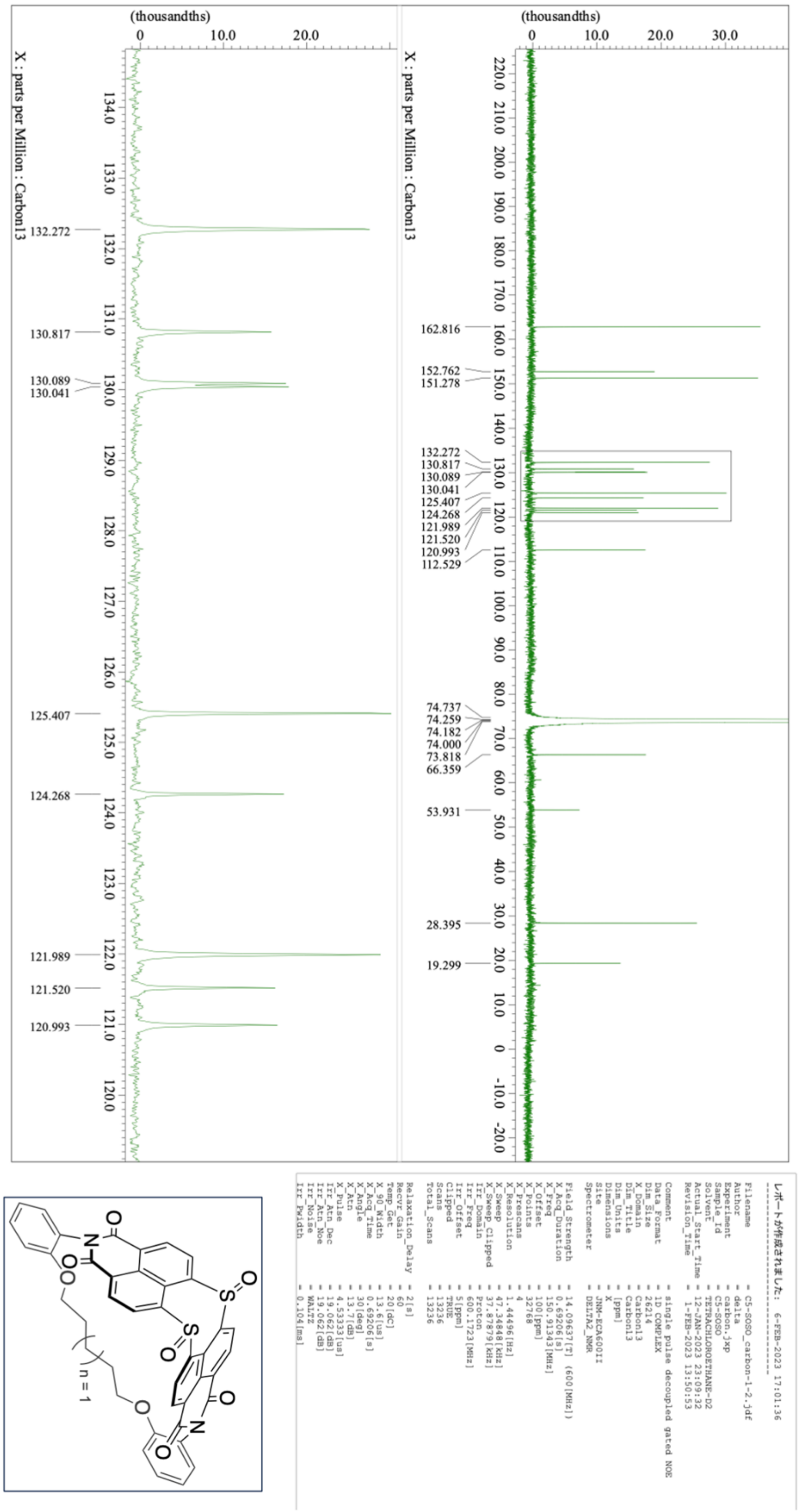

Figure S14. ^{13}C NMR spectrum of **4a** in 1,1,2,2-tetrachroloethane-d_2 at 25 °C.

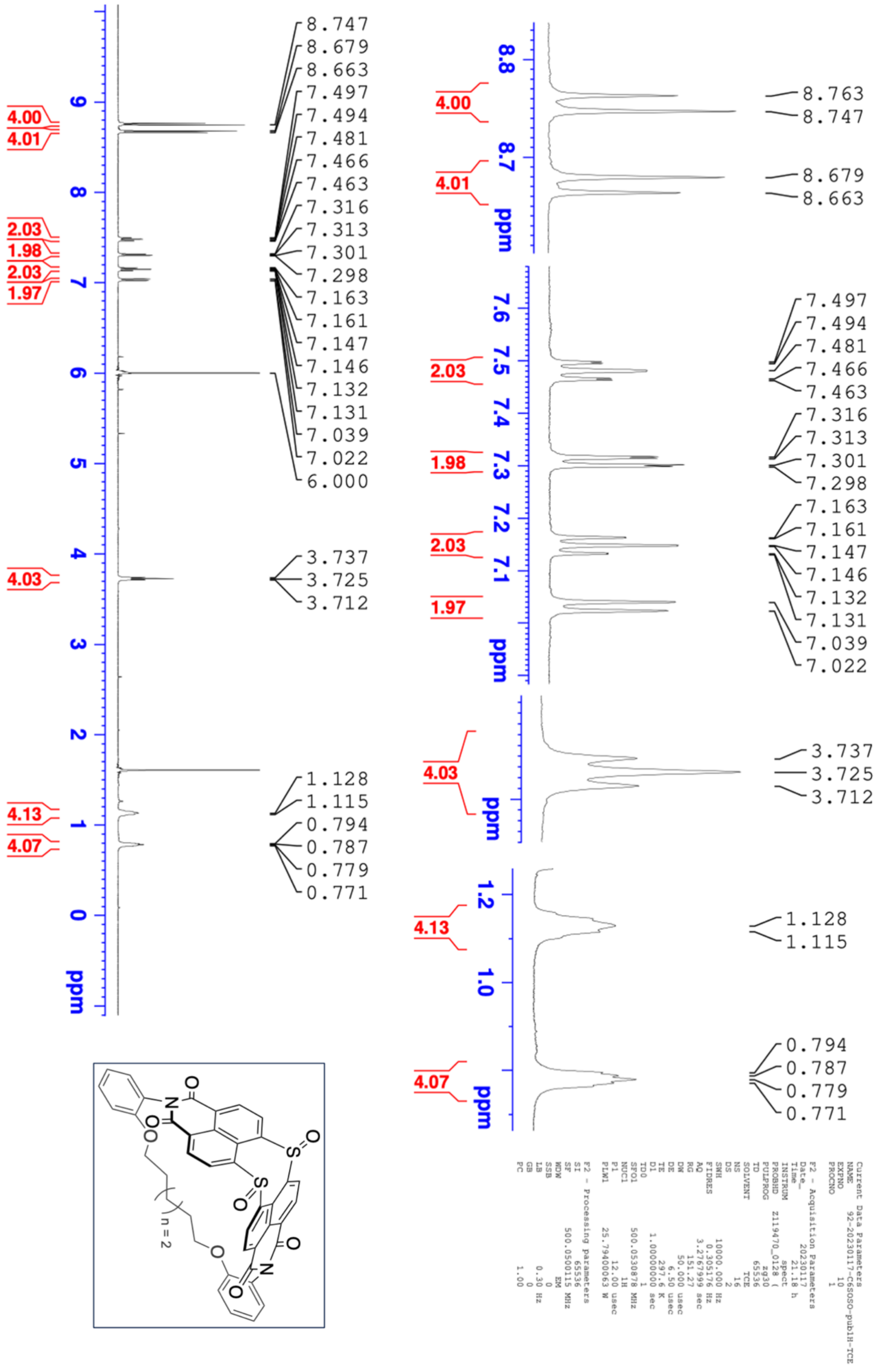

Figure S15. ^{1}H NMR spectrum of **4b** in 1,1,2,2-tetrachroloethane-d_2 at 25 °C.

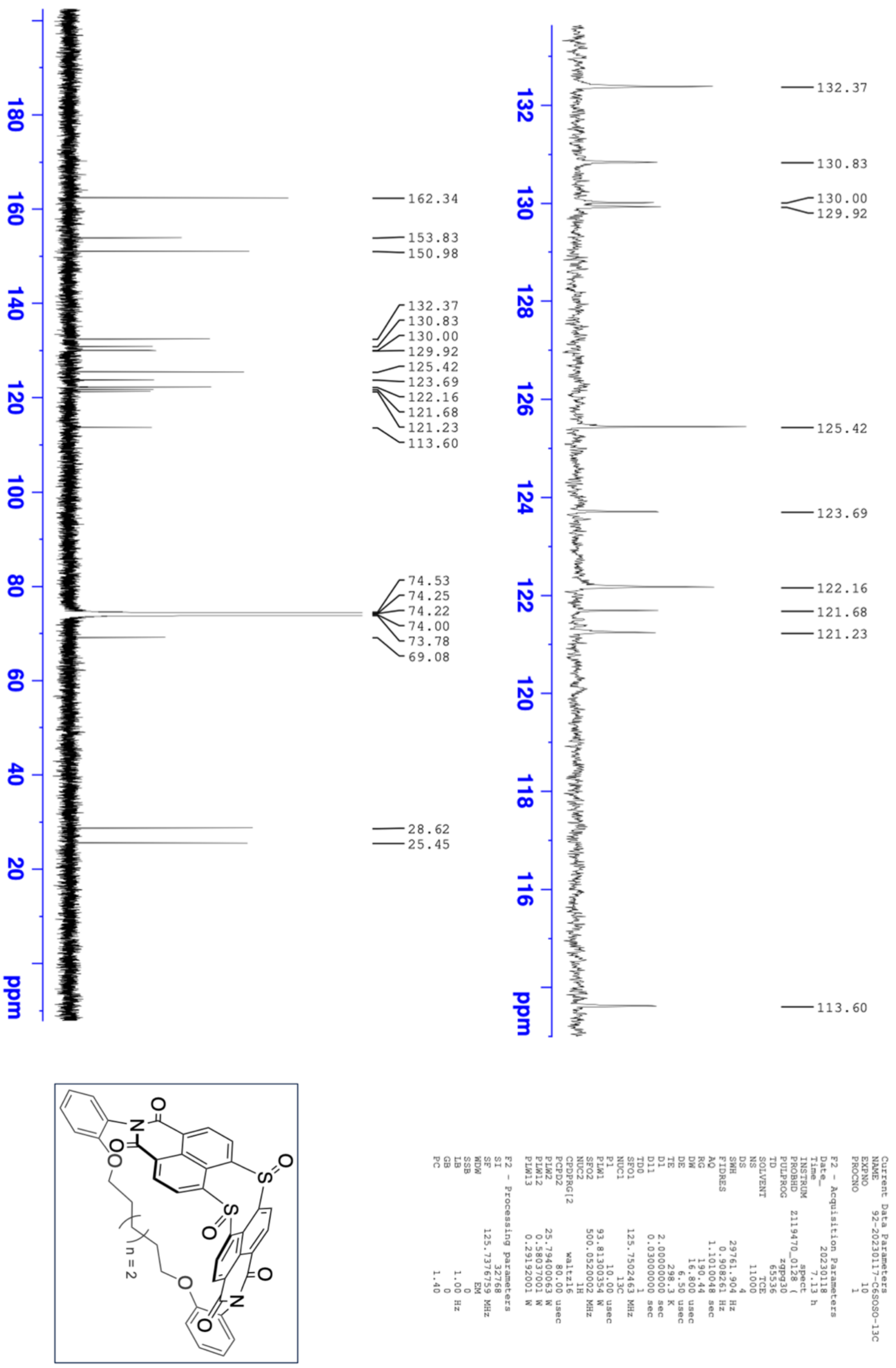

Figure S16. ^{13}C NMR spectrum of **4b** in 1,1,2,2-tetrachroloethane-d_2 at 25 °C.

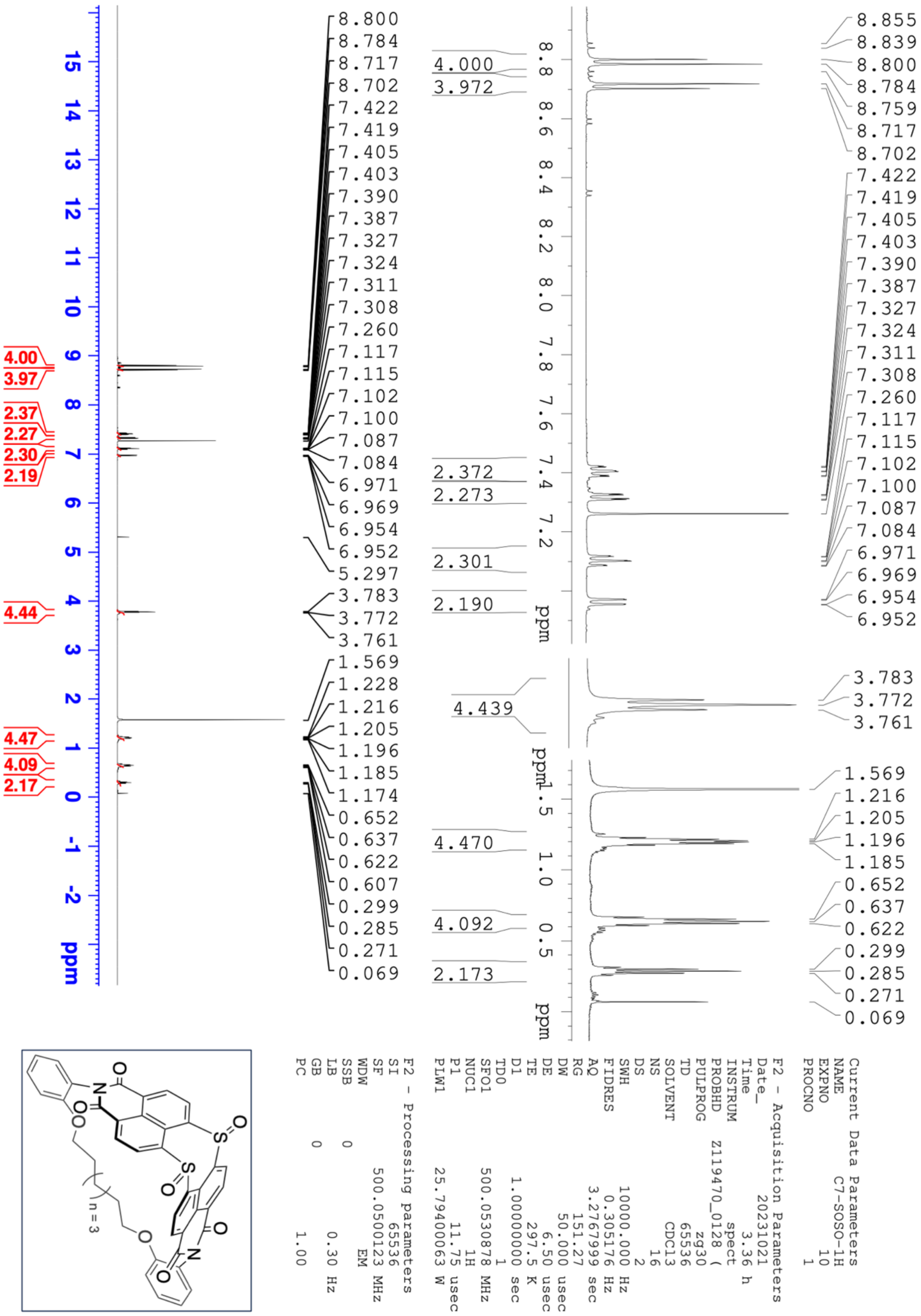

Figure S17. ^{1}H NMR spectrum of **4c** in $CDCl_3$ at 25 °C.

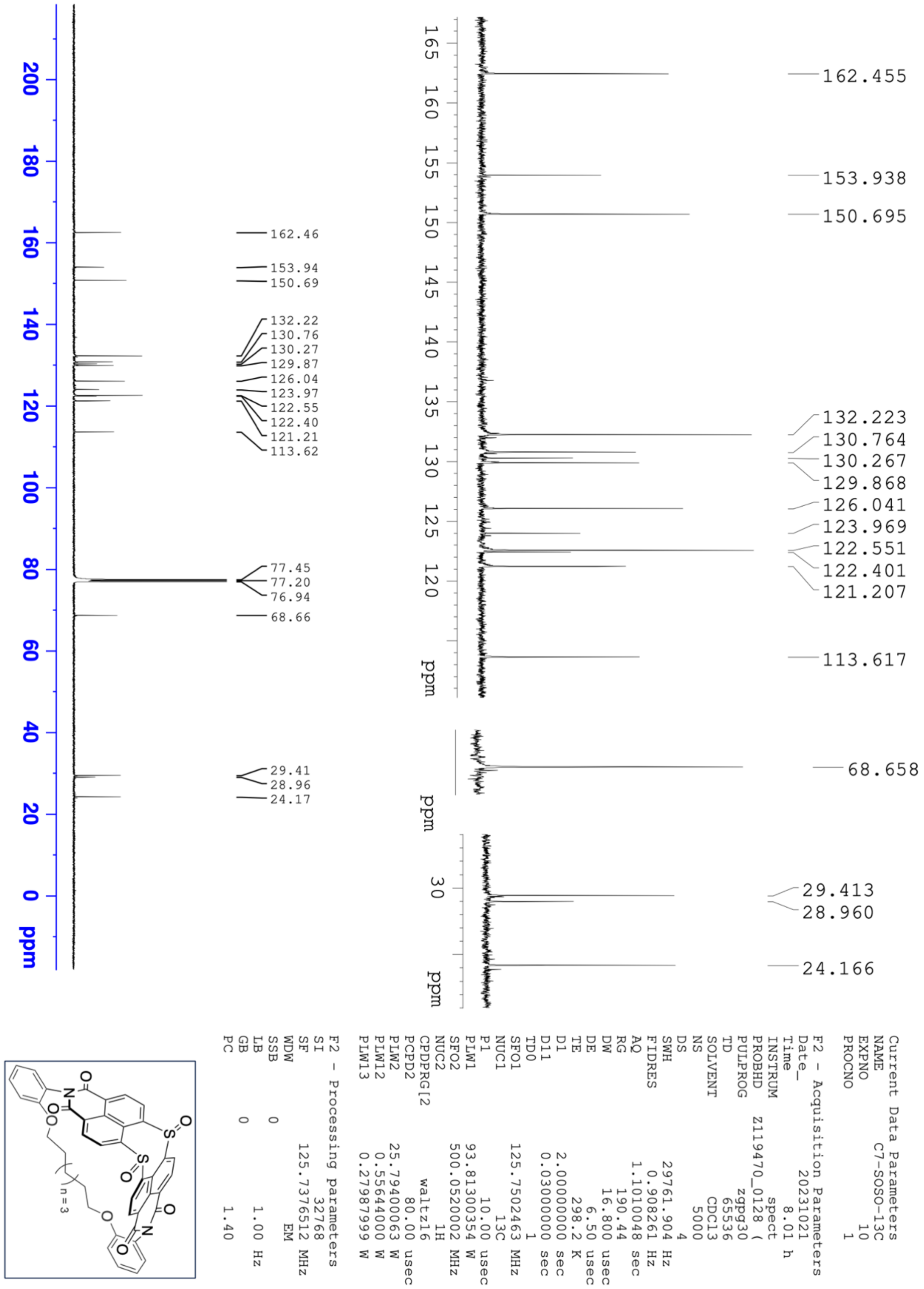

Figure S18. ^{13}C NMR spectrum of **4c** in $CDCl_3$ at 25 °C.

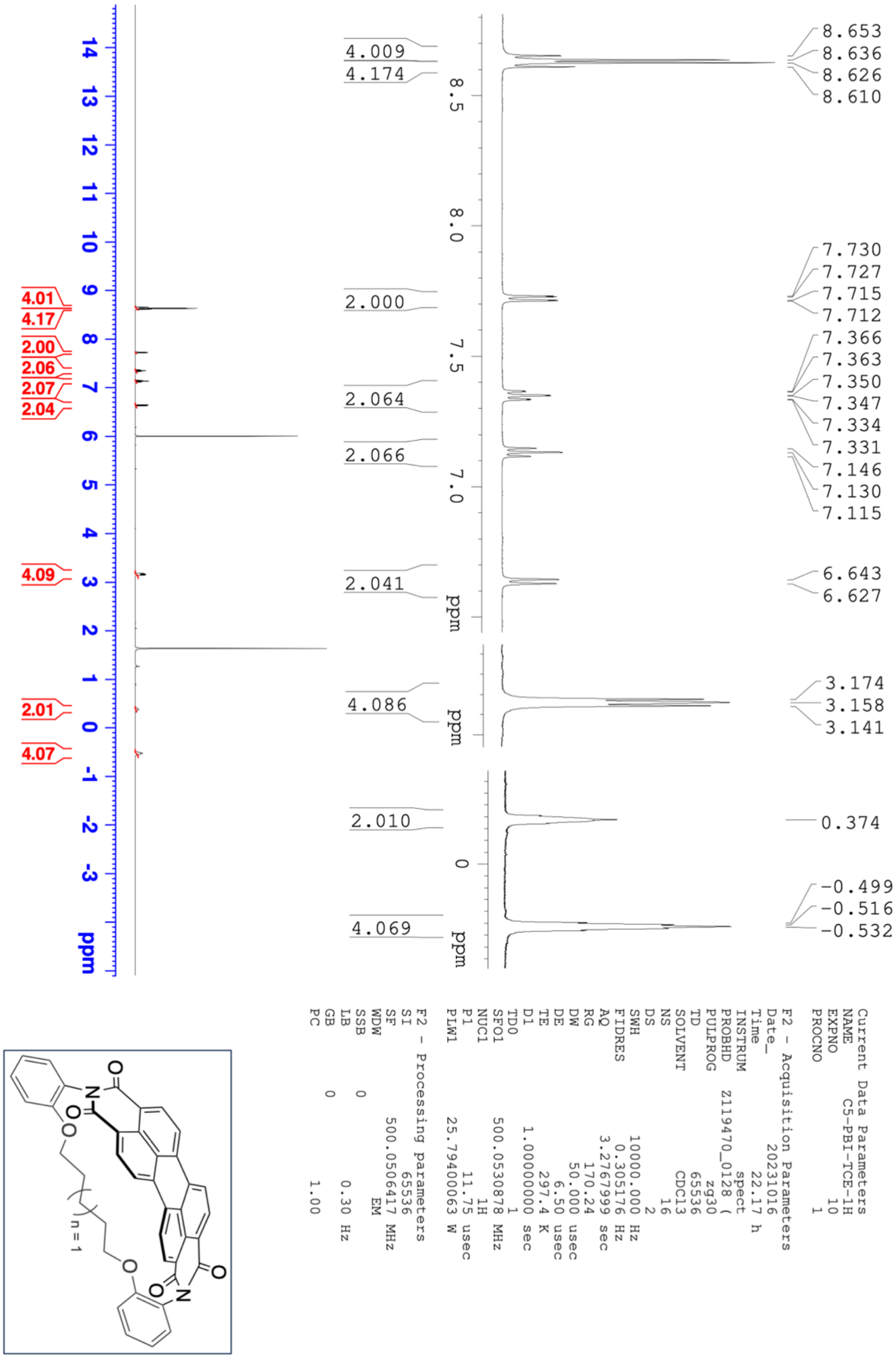

Figure S19. ^{1}H NMR spectrum of **5a** in 1,1,2,2-tetrachroloethane-d_2 at 25 °C.

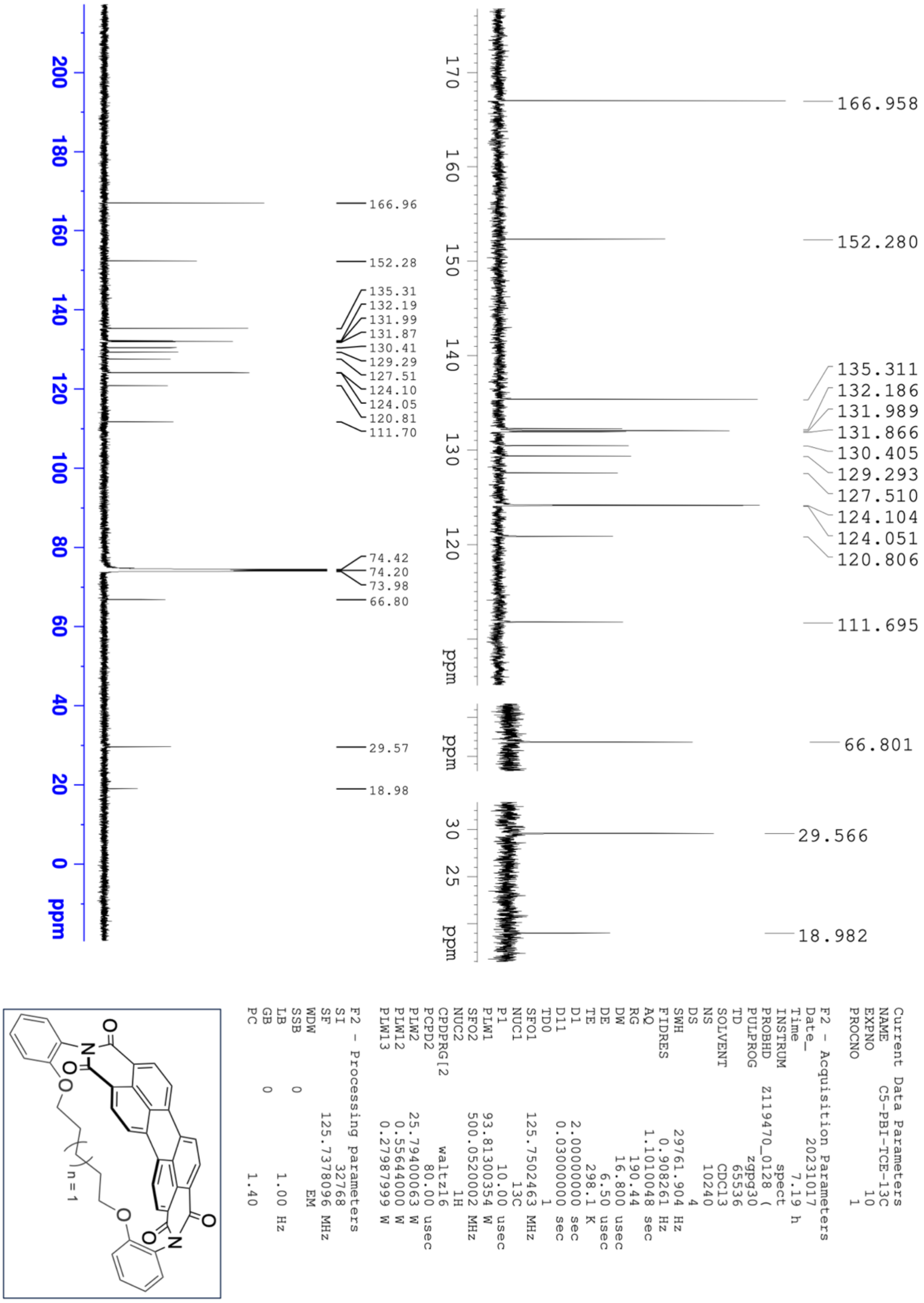

Figure S20. ^{13}C NMR spectrum of **5a** in 1,1,2,2-tetrachroloethane-d_2 at 25 °C.

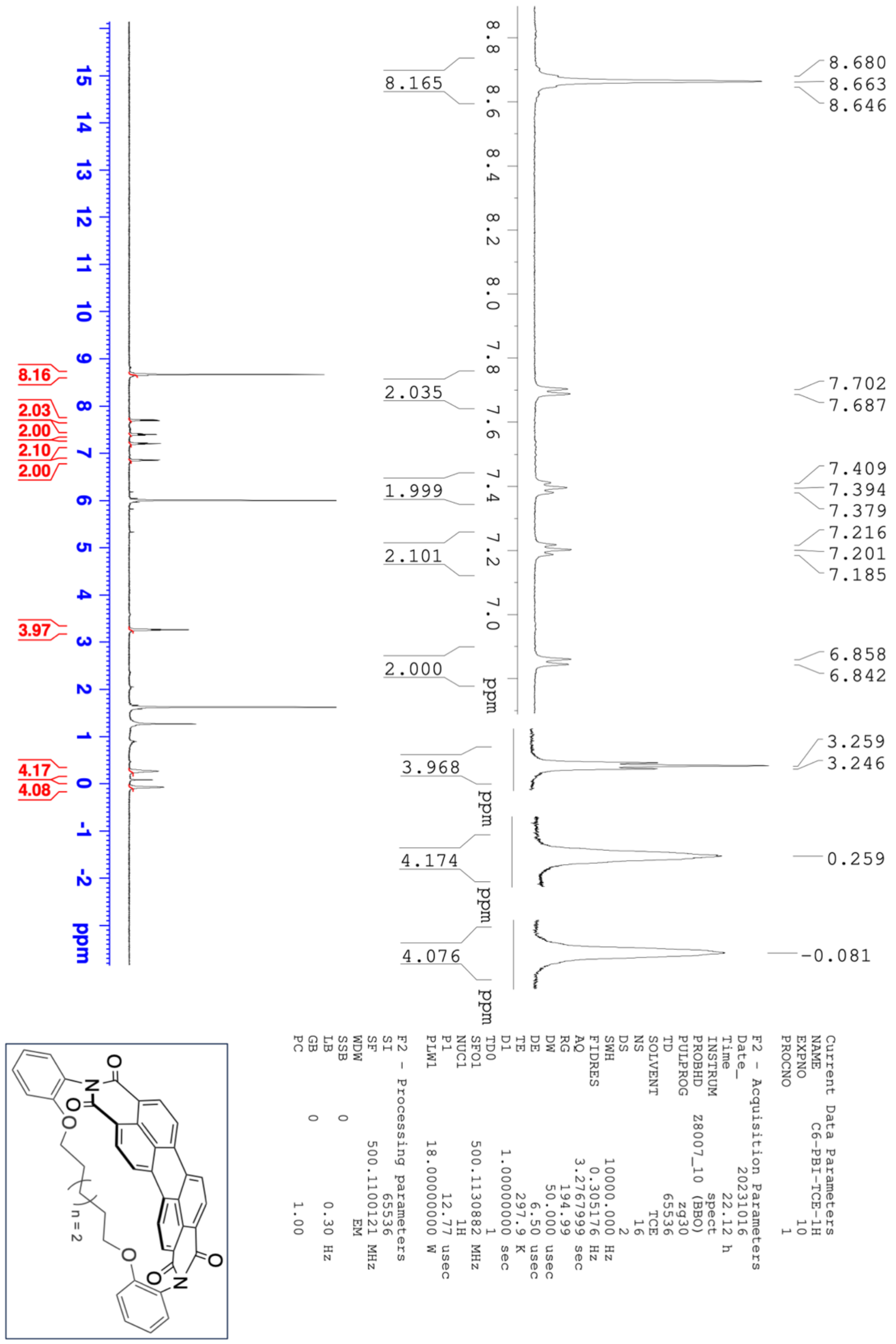

Figure S21. ^{1}H NMR spectrum of **5b** in 1,1,2,2-tetrachroloethane-d_2 at 25 °C.

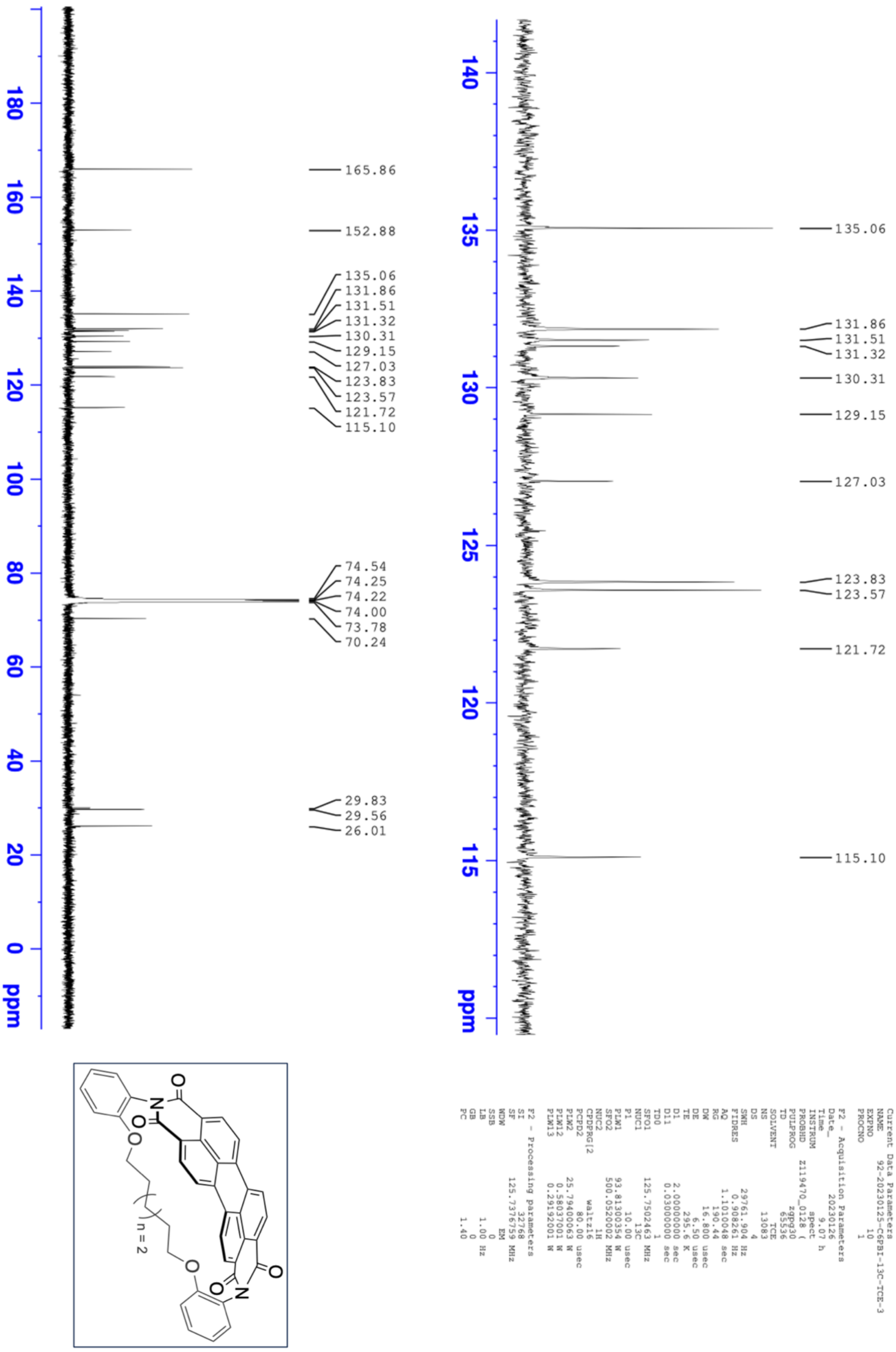

Figure S22. ^{13}C NMR spectrum of **5b** in 1,1,2,2-tetrachroloethane-d_2 at 25 °C.

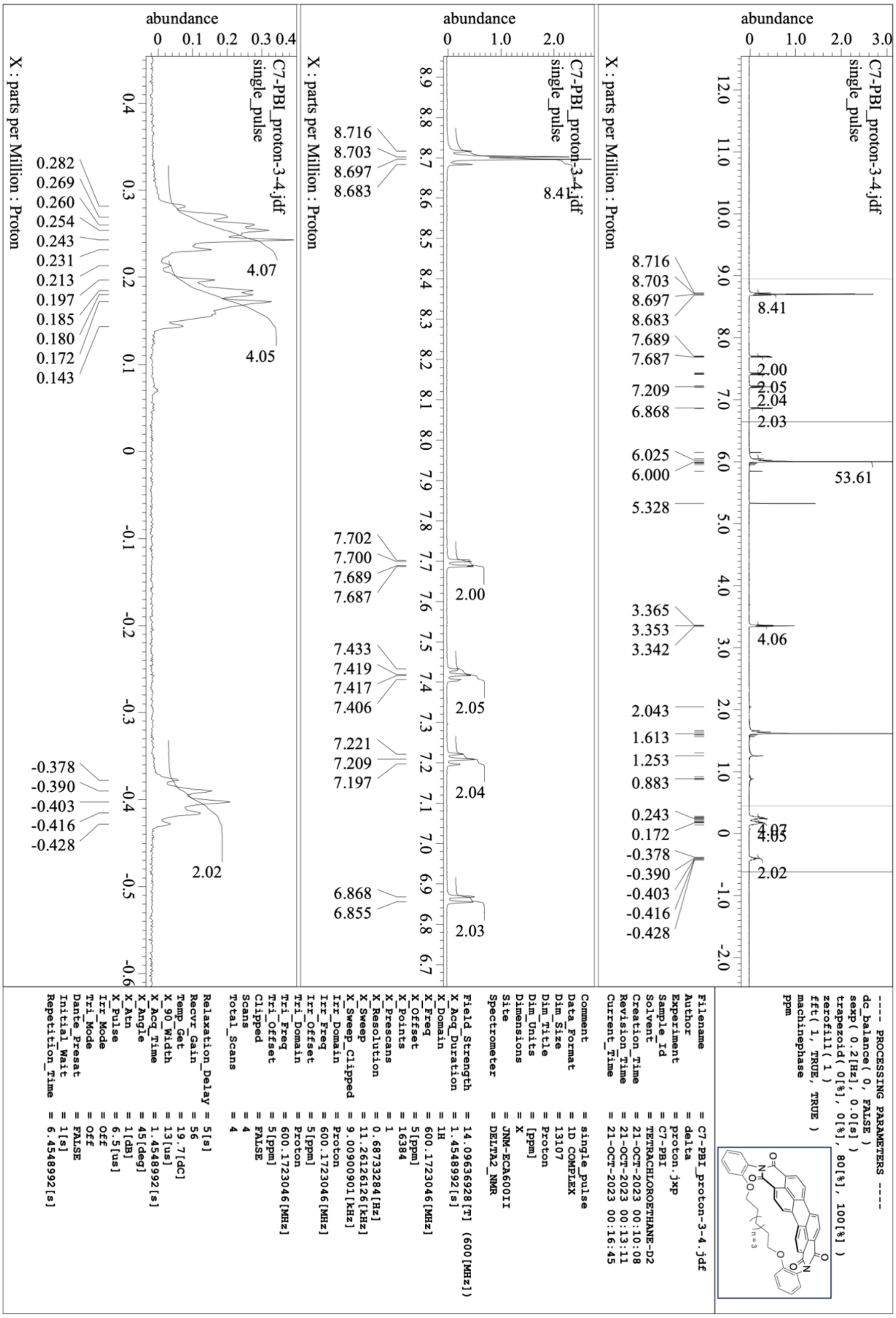

Figure S23. ^{1}H NMR spectrum of **5c** in 1,1,2,2-tetrachroloethane-d_2 at 25 °C.

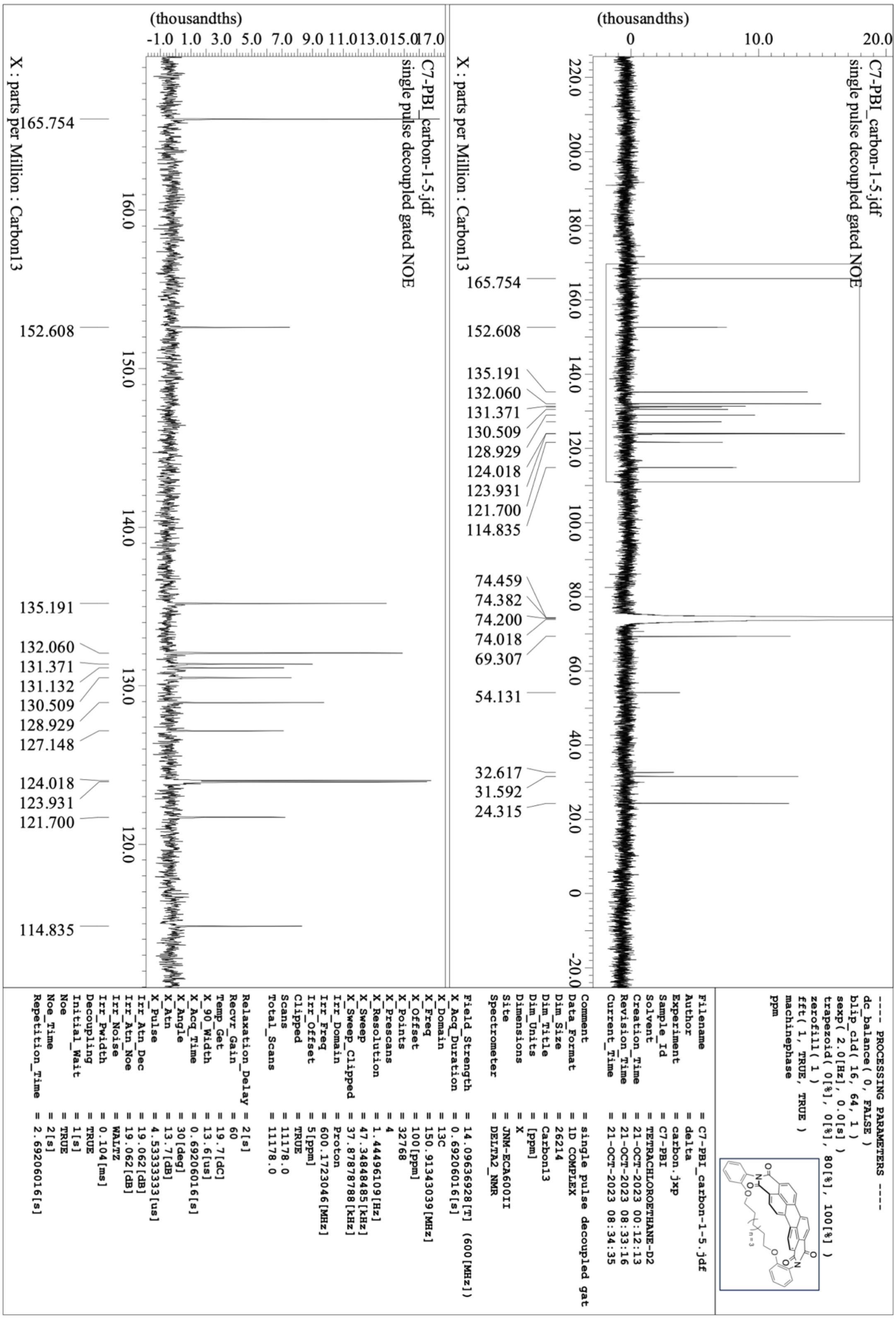

Figure S24. ^{13}C NMR spectrum of **5c** in 1,1,2,2-tetrachroloethane-d_2 at 25 °C.

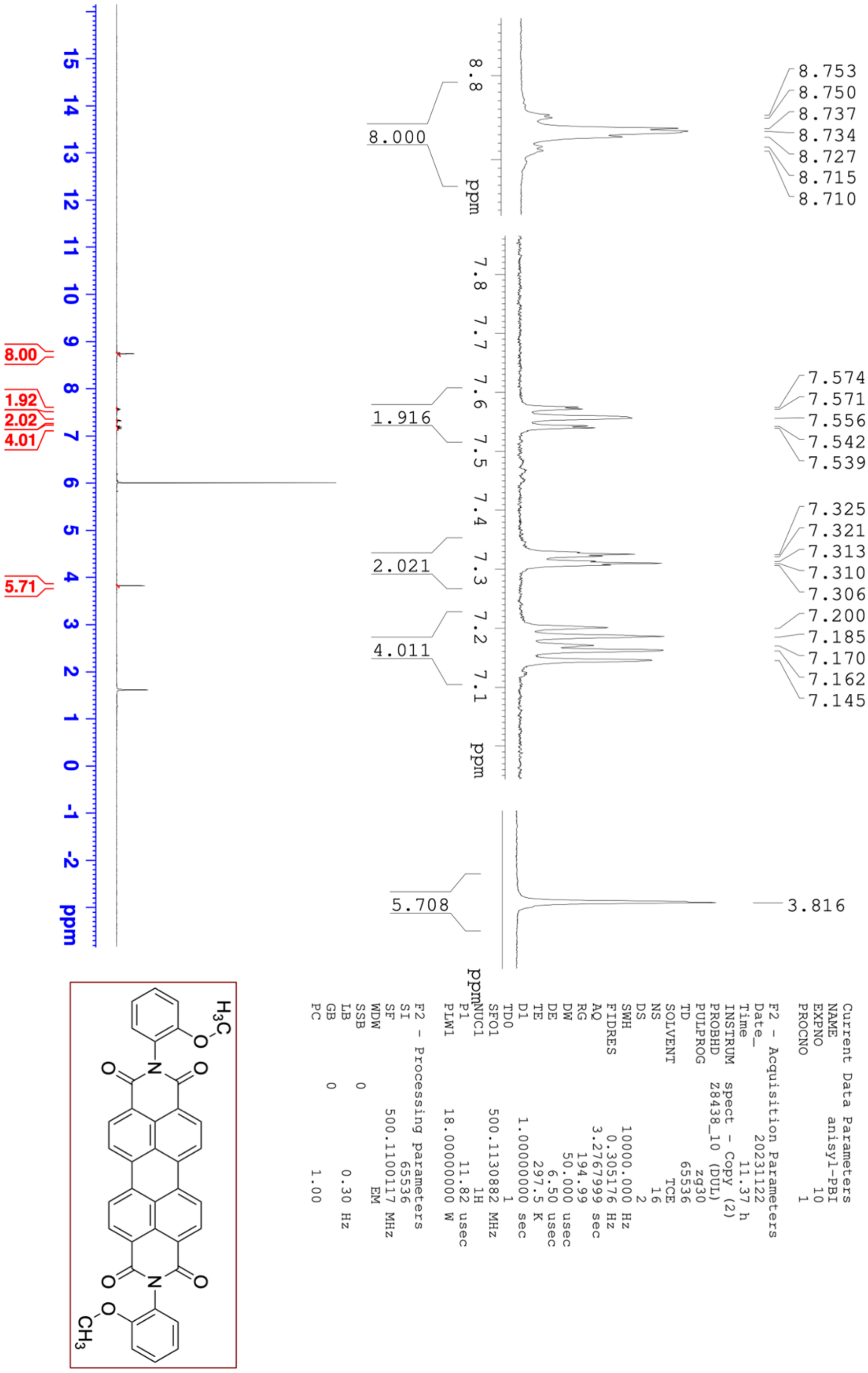

Figure S25. ^{1}H NMR spectrum of **3″** in 1,1,2,2-tetrachroloethane-d_2 at 25 °C.

4. Mass spectra

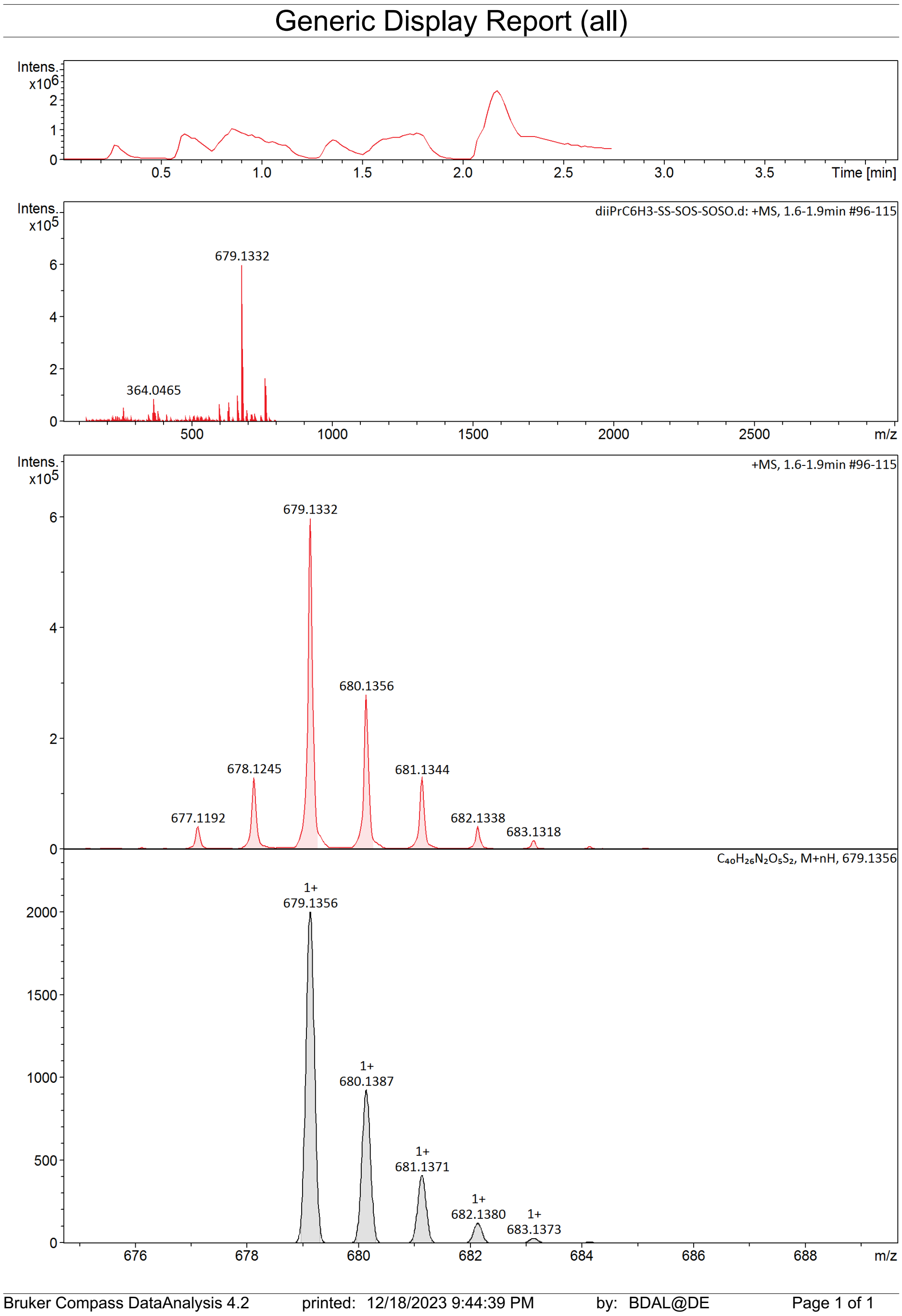

Figure S26. APCI-TOF mass spectrum of **10**.

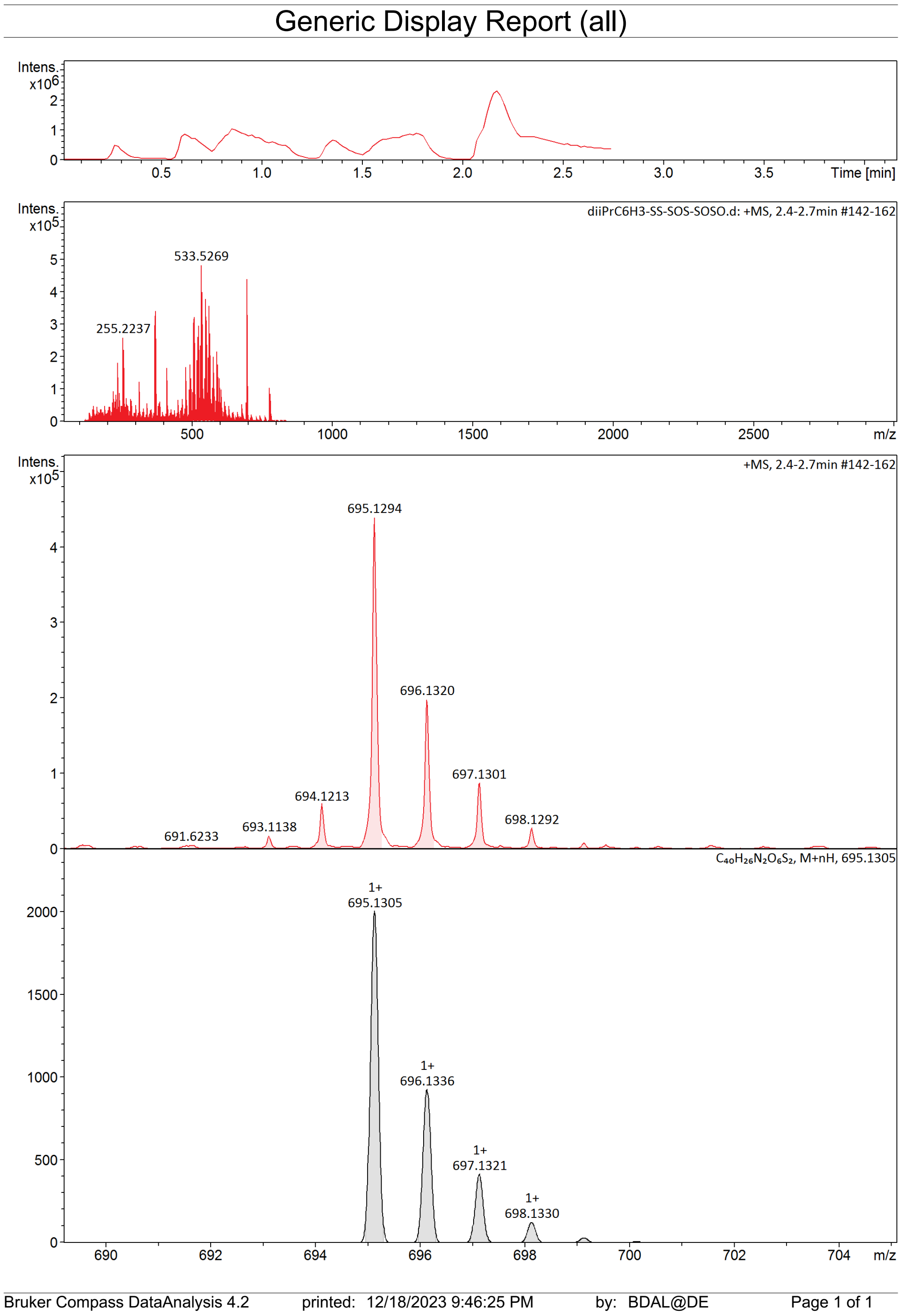

Figure S27. APCI-TOF mass spectrum of **11**.

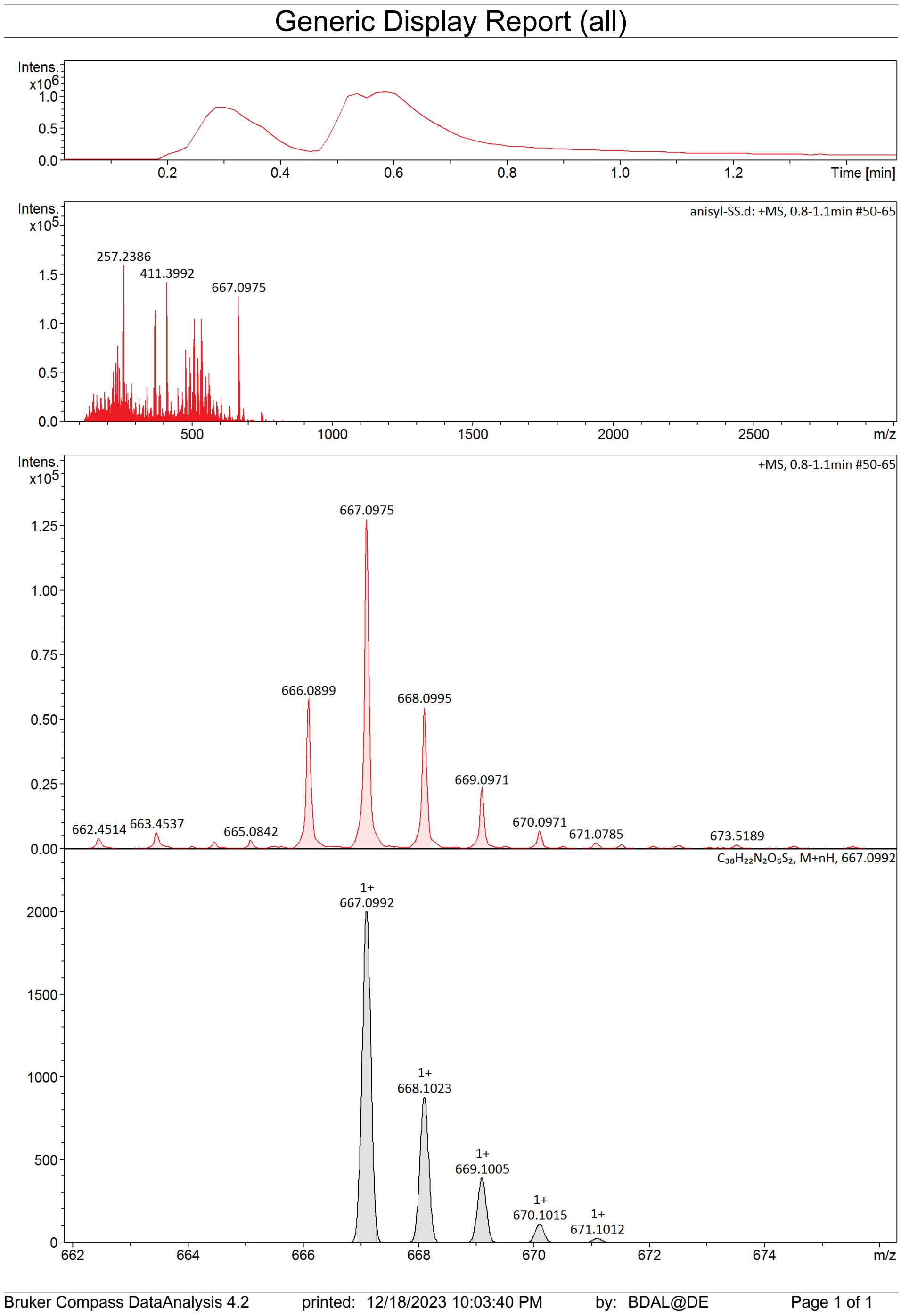

Figure S28. APCI-TOF mass spectrum of **13**.

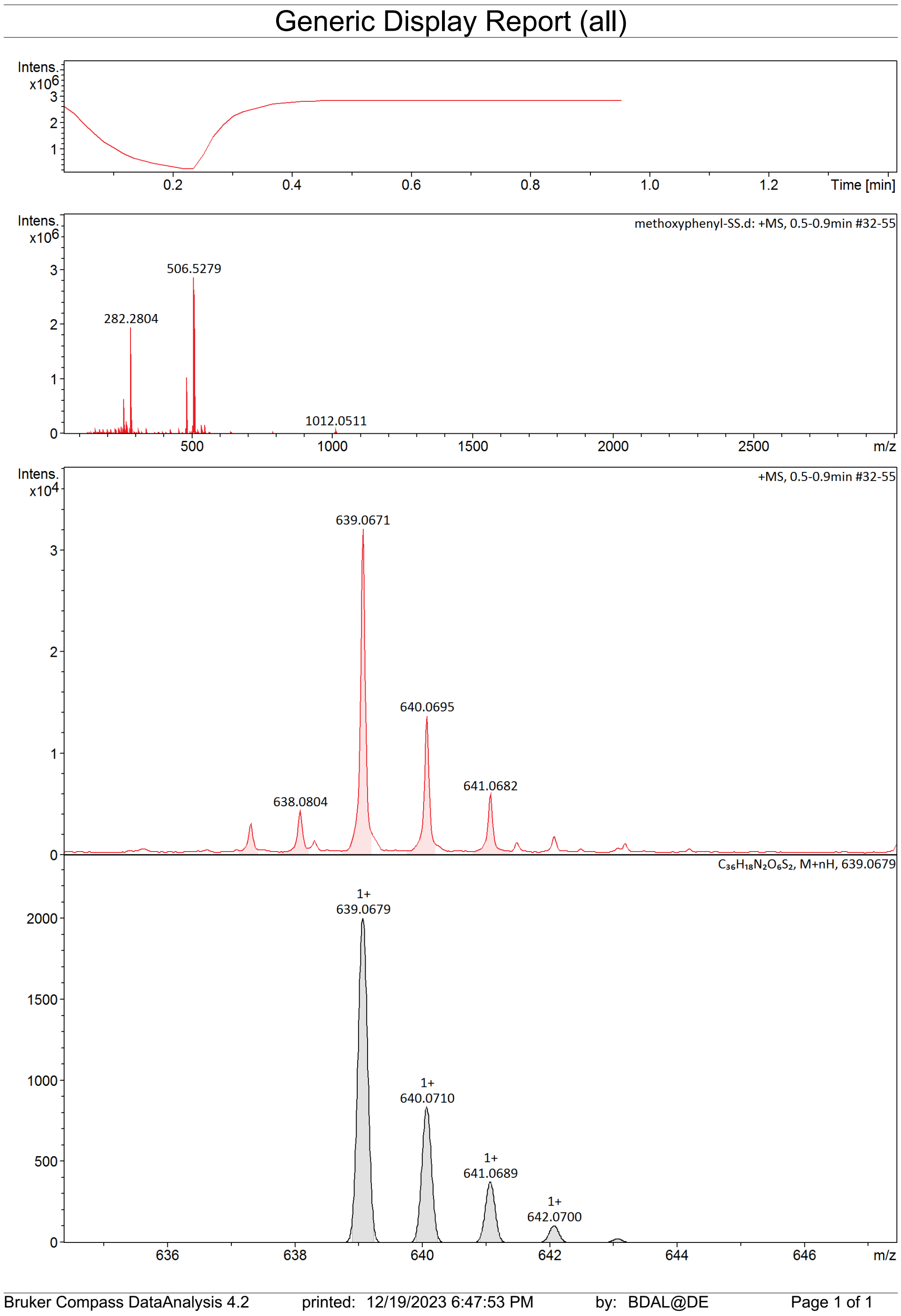

Figure S29. APCI-TOF mass spectrum of **14**.

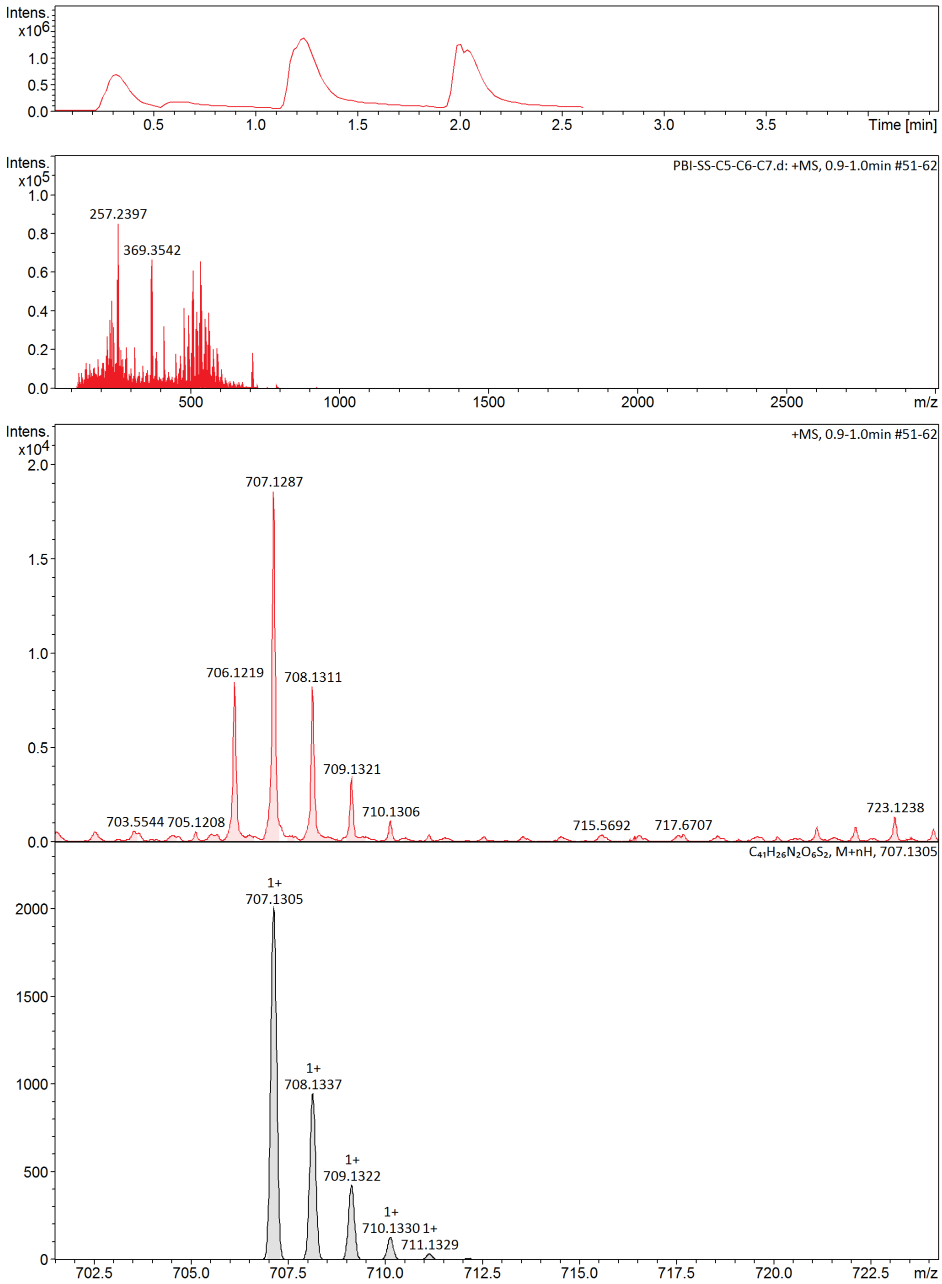

Figure S30. APCI-TOF mass spectrum of **15a**.

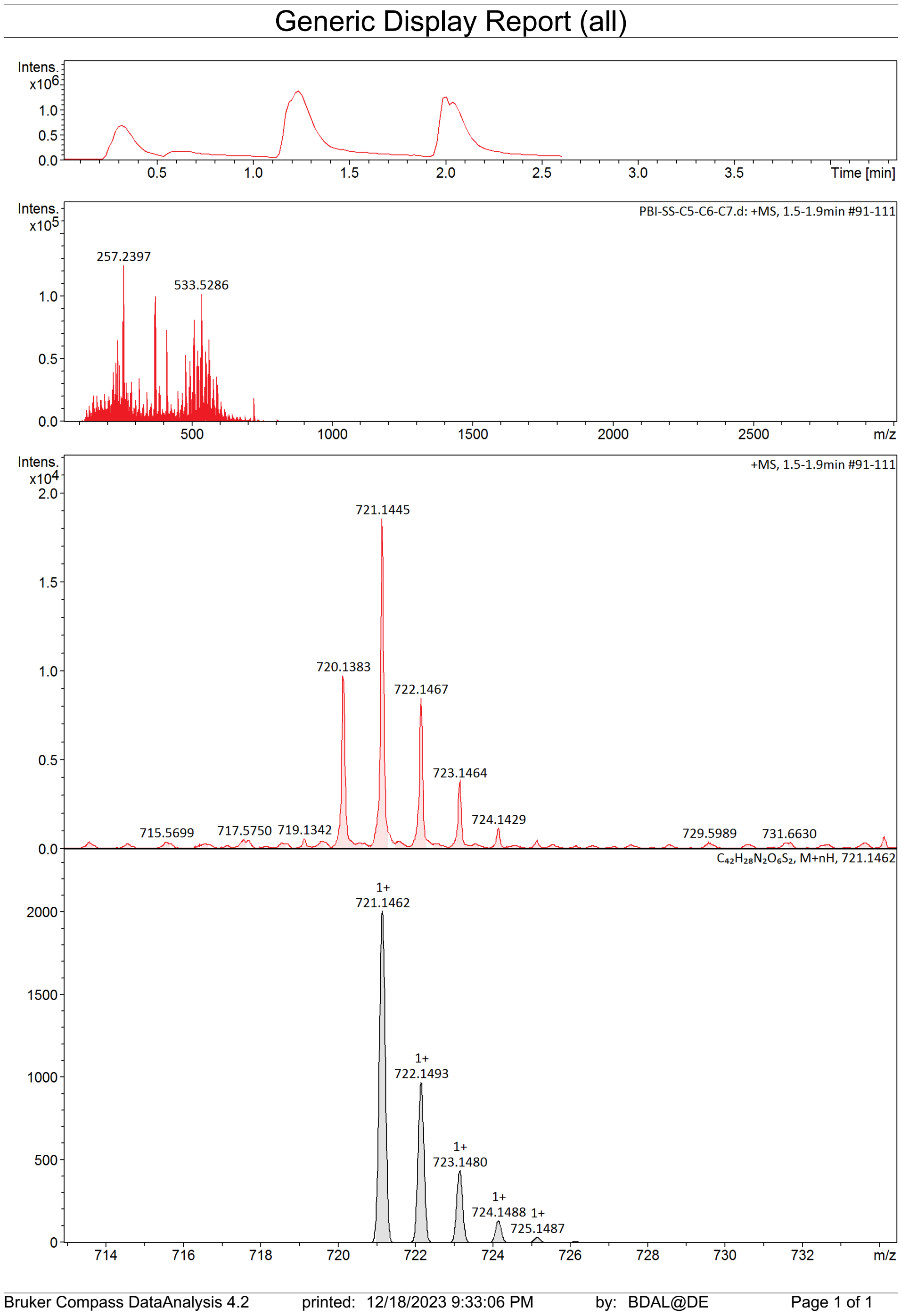

Figure S31. APCI-TOF mass spectrum of **15b**.

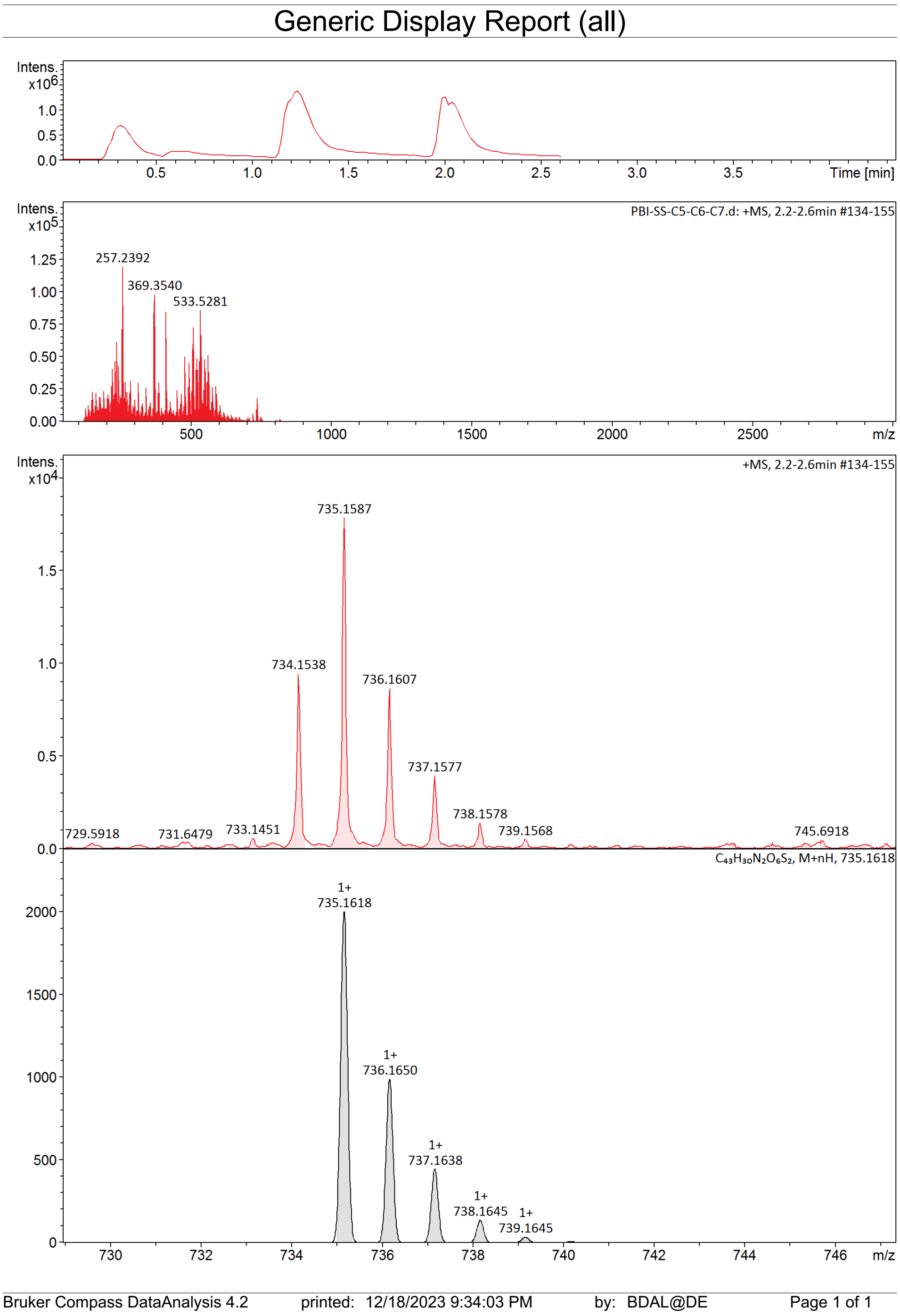

Figure S32. APCI-TOF mass spectrum of **15c**.

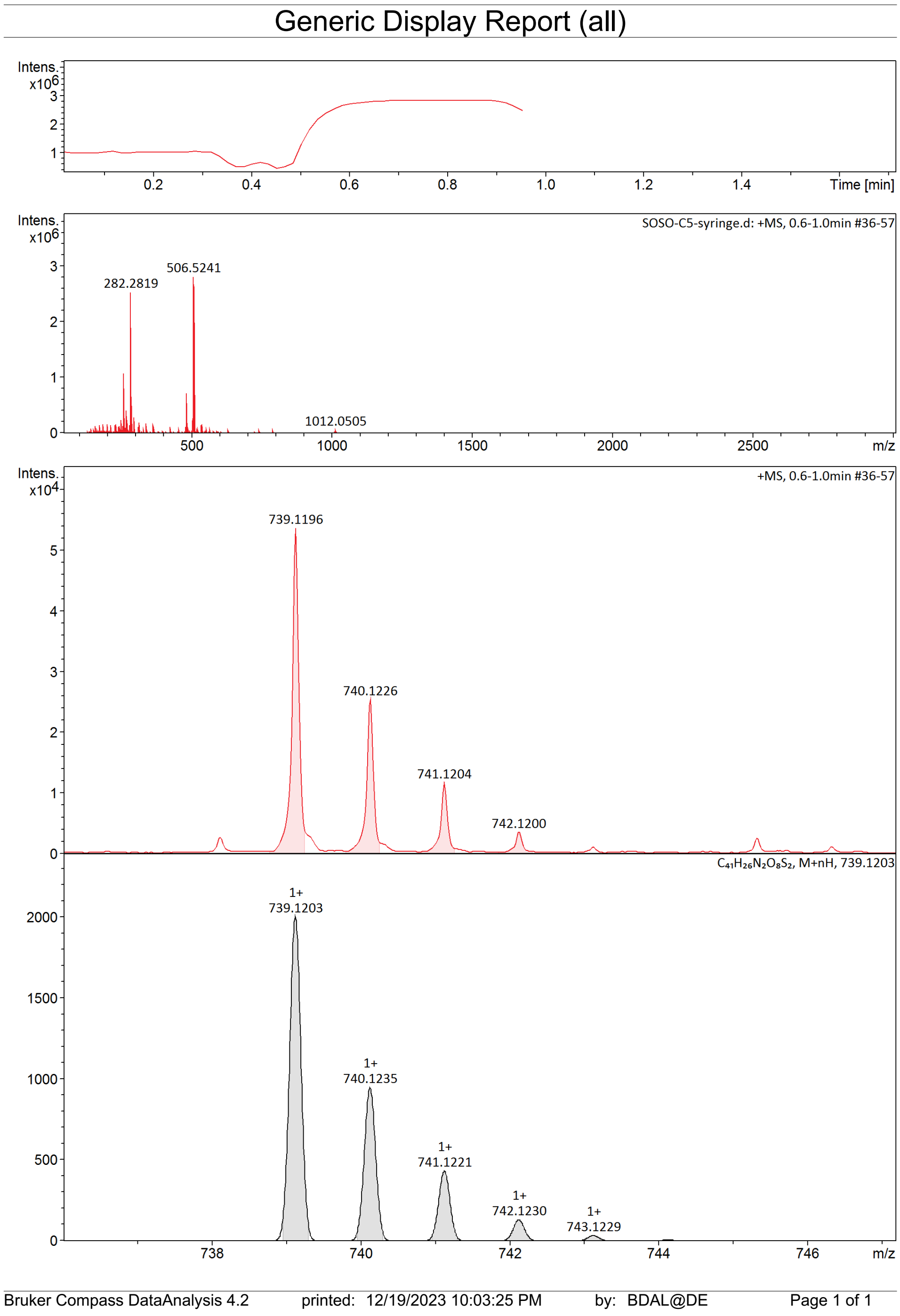

Figure S33. APCI-TOF mass spectrum of **4a**.

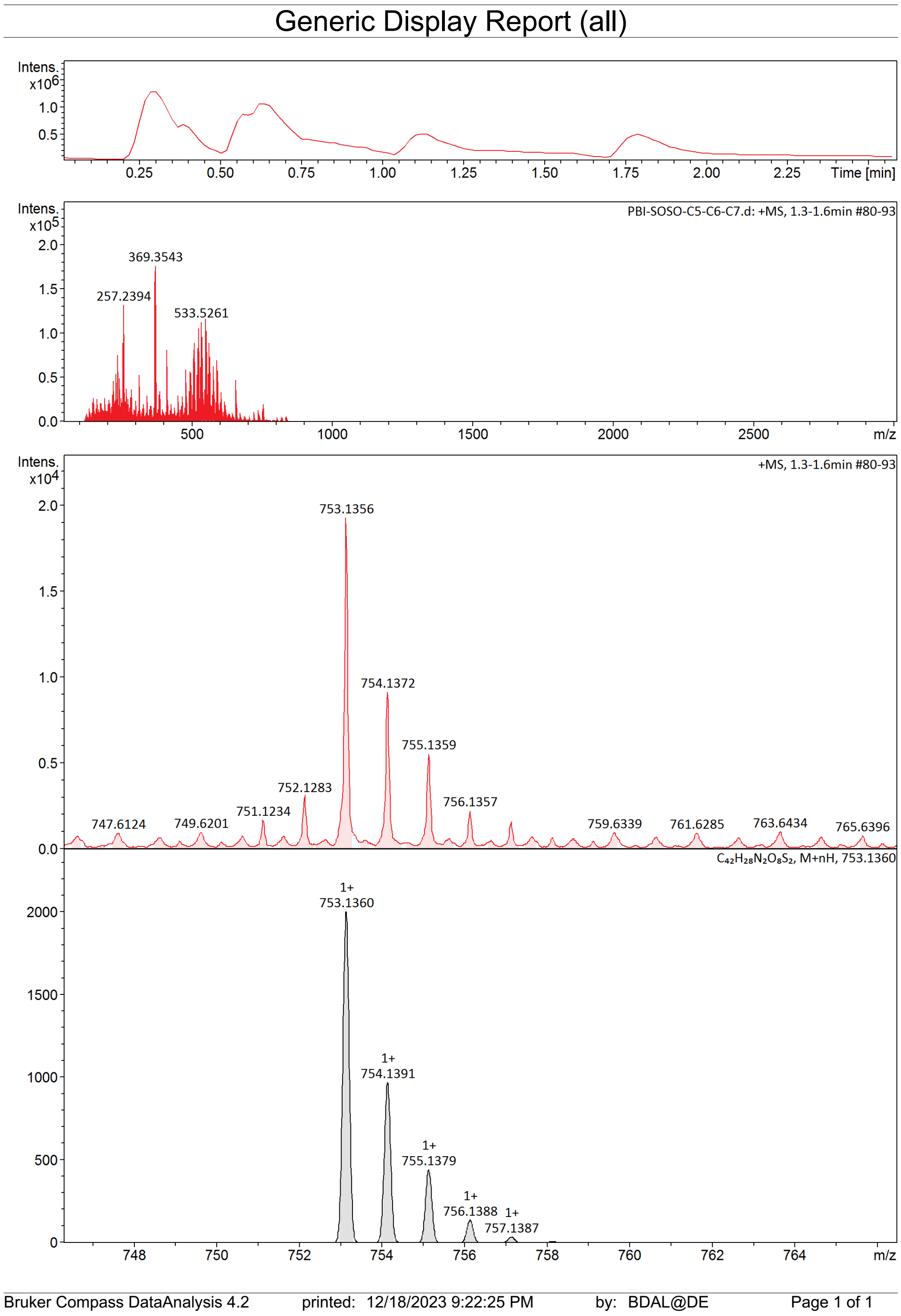

Figure S34. APCI-TOF mass spectrum of **4b**.

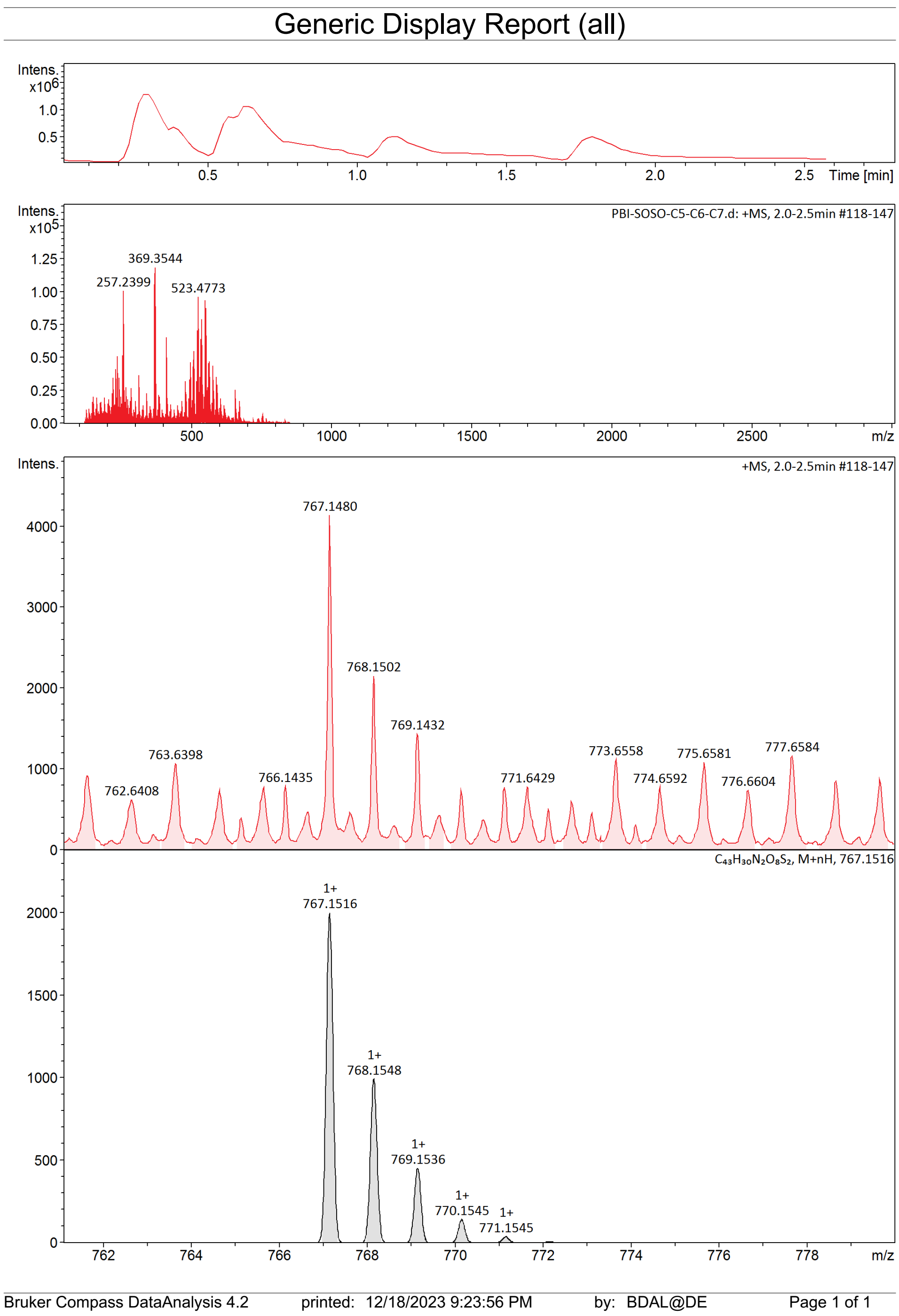

Figure S35. APCI-TOF mass spectrum of **4c**.

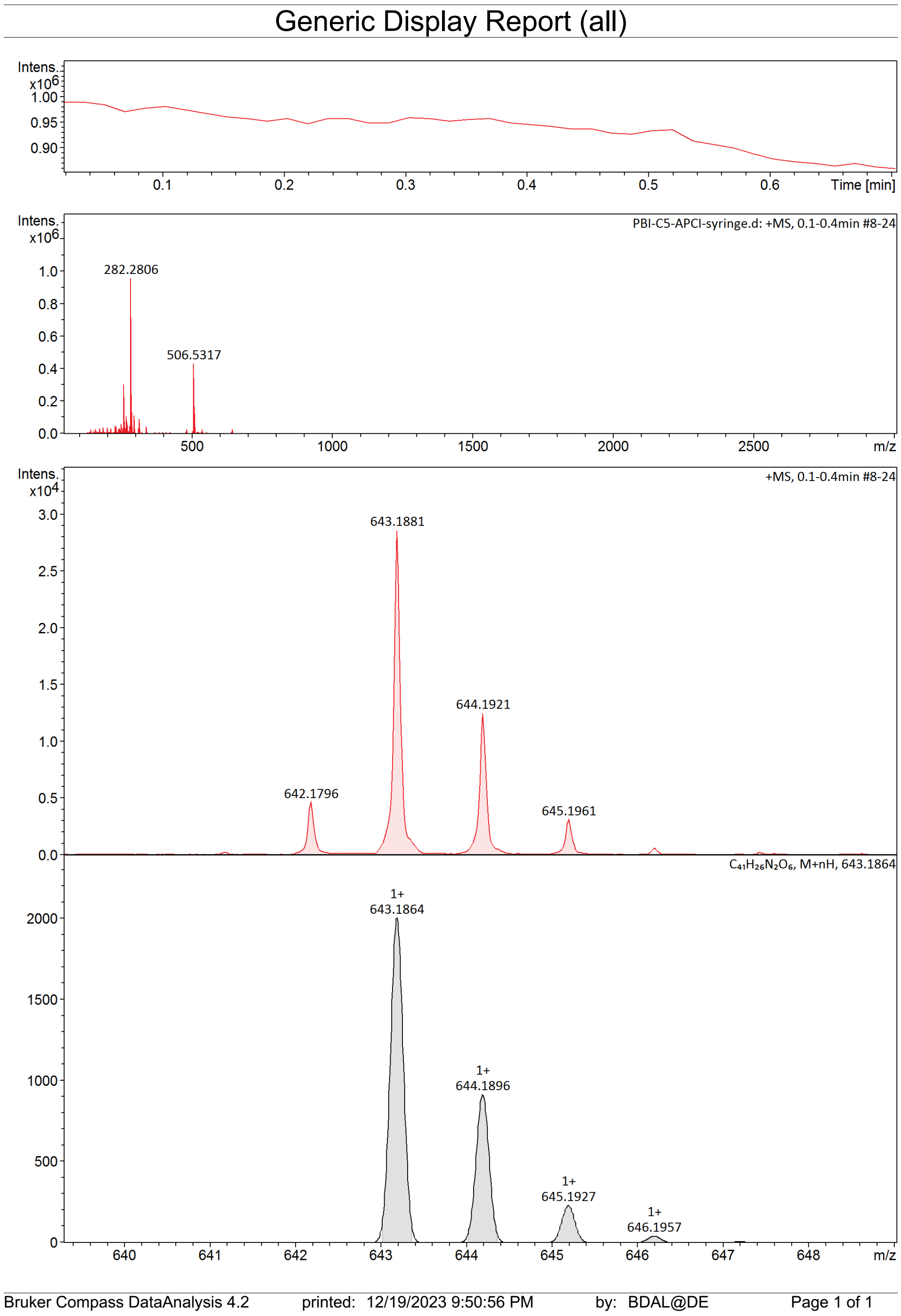

Figure S36. APCI-TOF mass spectrum of **5a**.

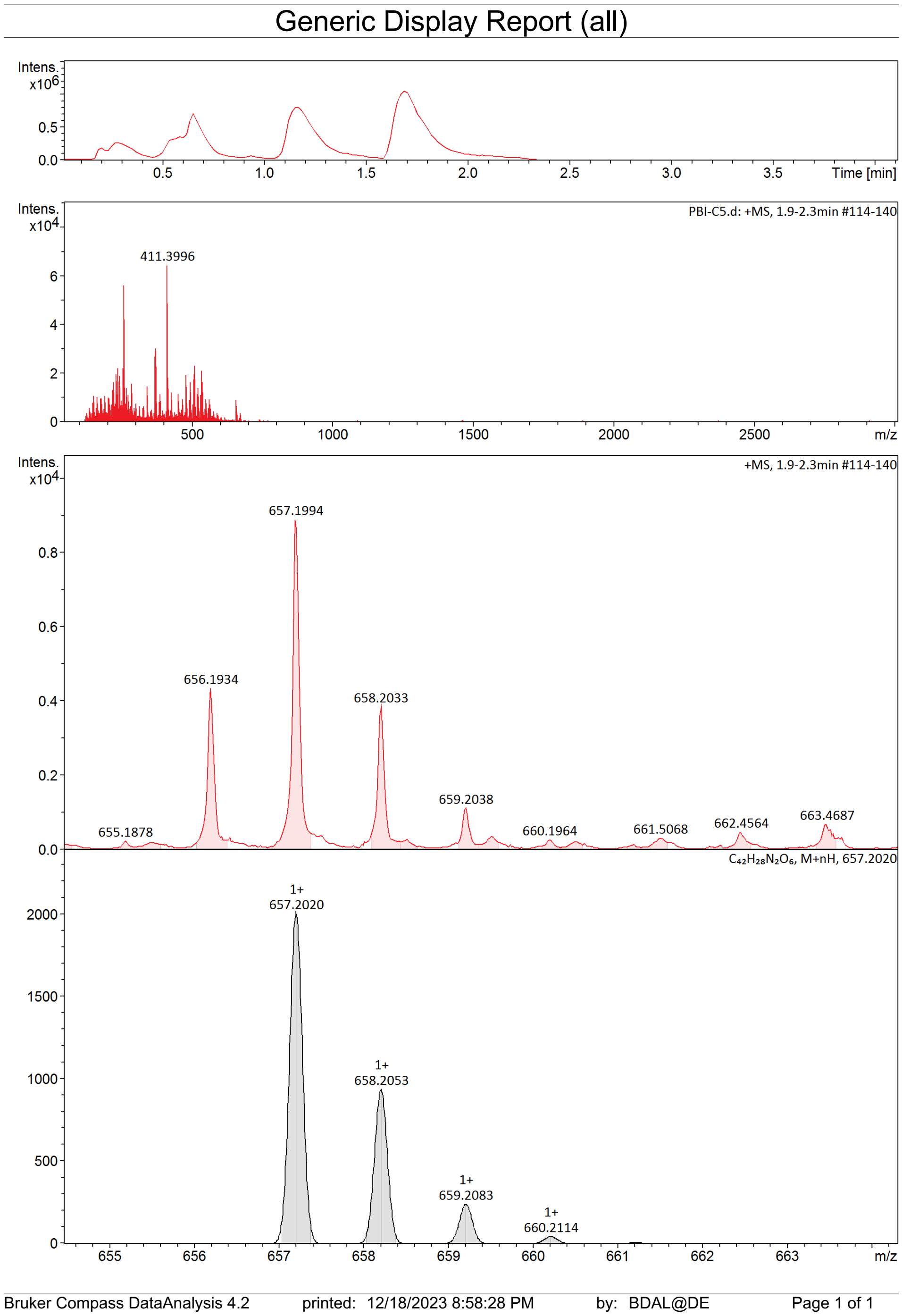

Figure S37. APCI-TOF mass spectrum of **5b**.

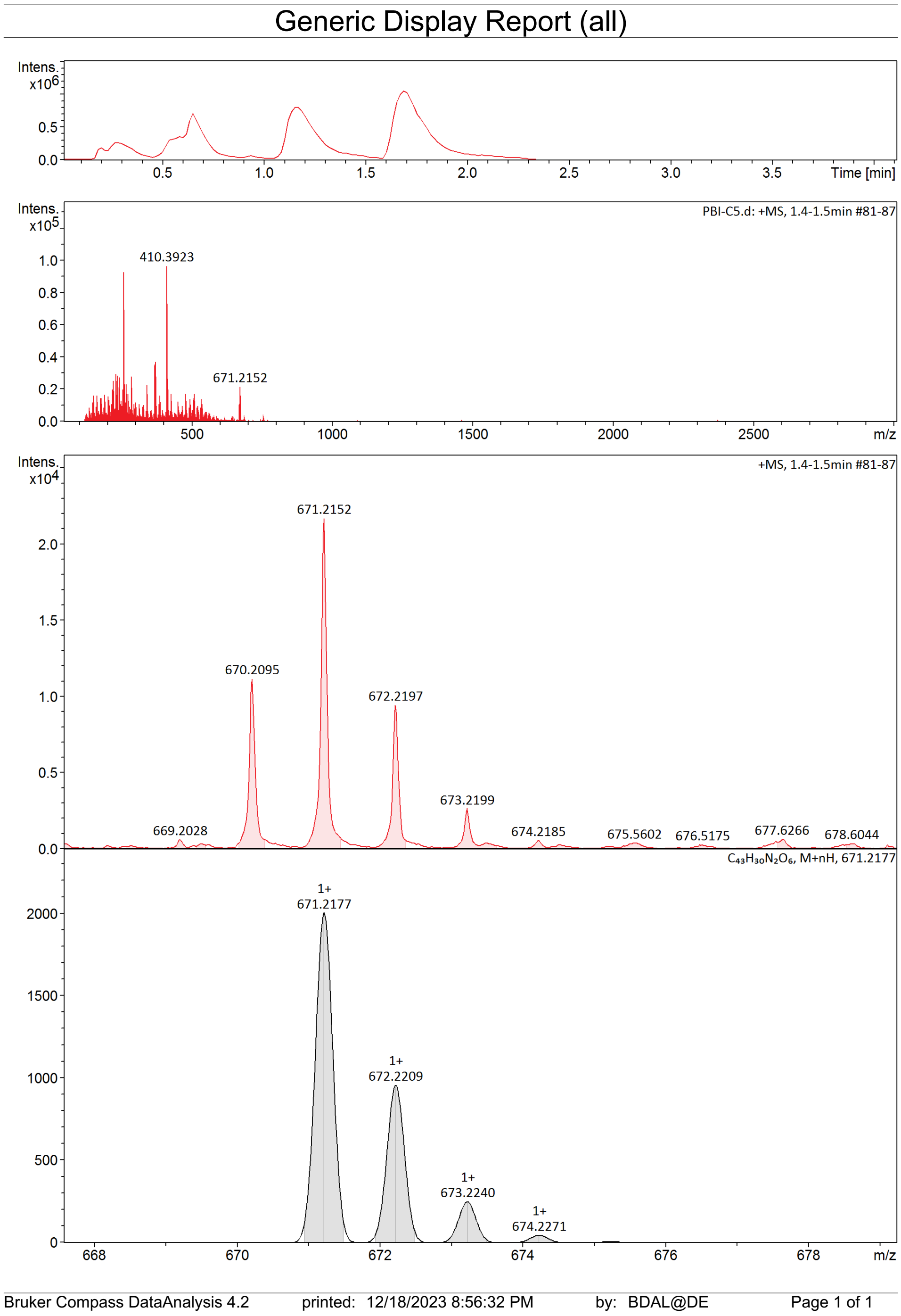

Figure S38. APCI-TOF mass spectrum of **5c**.

5. Crystal data

Table S1. Crystallographic data of **5a**, **5b**, and **5c**.

Compound	**5a**	**5b**	**5c**
Formula	$C_{41}H_{26}N_2O_6$	$C_{42}H_{28}N_2O_6$, $CHCl_3$	$2(C_{43}H_{30}N_2O_6)$
Formula weight	642.64	776.03	1341.38
Crystal system	monoclinic	monoclinic	monoclinic
Space group	*I*12/a1 (No. 15)	$P2_1/a$ (No. 14)	$P12_1/n1$ (No. 14)
Crystal color	red	red	red
Crystal description	plate	plate	block
a [Å]	15.7272(6)	11.8210(5)	13.6337(3)
b [Å]	11.3327(3)	24.9798(9)	11.8657(3)
c [Å]	20.9939(8)	12.1425(5)	19.1562(5)
α [°]	—	—	—
β [°]	109.582(4)	106.628(4)	98.107(2)
γ [°]	—	—	—
V [$Å^3$]	3525.4(2)	3435.6(2)	3067.99(13)
Z	4	4	2
d_{calcd} [g cm^{-3}]	1.328	1.500	1.452
R_1 ($I > 2\sigma(I)$)	0.0499	0.0619	0.0562
wR_2 (all data)	0.1238	0.1800	0.1435
Goodness-of-fit	1.013	1.146	1.029
Temperature [K]	93(2)	93(2)	93(2)
CCDC No.	2348376	2348377	2348378

Table S2. Crystallographic data of **10** and **11**.

Compound	**10**	**11**
Formula	$C_{40}H_{26}N_2O_4S_2$, 2.73($CH_3C_6H_5$), 0.27(CH_2Cl_2)	$C_{40}H_{26}N_2O_6S_2$
Formula weight	593.20	933.48
Crystal system	triclinic	triclinic
Space group	*P*–1 (No. 2)	*P*–1 (No. 2)
Crystal color	yellow	colorless
Crystal description	plate	block
a [Å]	12.9868(4)	10.1116(10)
b [Å]	13.0586(5)	13.6115(9)
c [Å]	14.4262(4)	15.4513(13)
α [°]	80.800(3)	83.734(6)
β [°]	81.409(3)	73.598(8)
γ [°]	74.562(3)	68.554(7)
V [Å^3]	2313.21(14)	1898.8(3)
Z	2	2
d_{calcd} [g cm^{-3}]	1.369	1.633
R_1 ($I > 2\sigma(I)$)	0.0835	0.0781
wR_2 (all data)	0.2766	0.2468
Goodness-of-fit	1.044	1.037
Temperature [K]	93(2)	93(2)
CCDC No.	2348379	2348380

6. Electrochemical properties

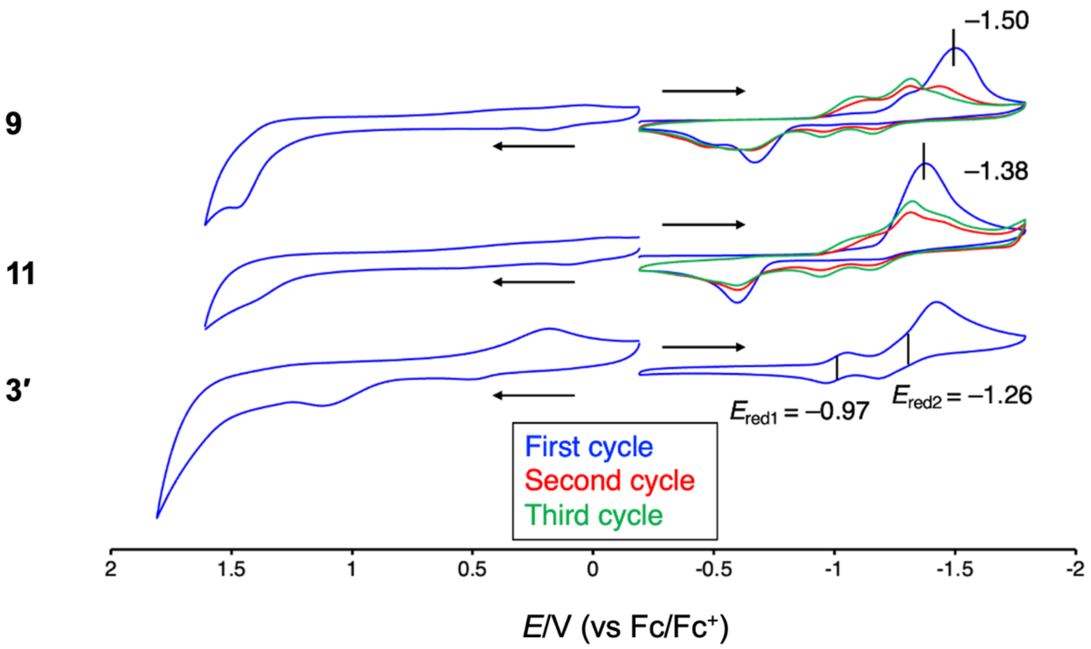

Figure S39. Cyclic voltammograms of **3′**, **9**, and **11**.

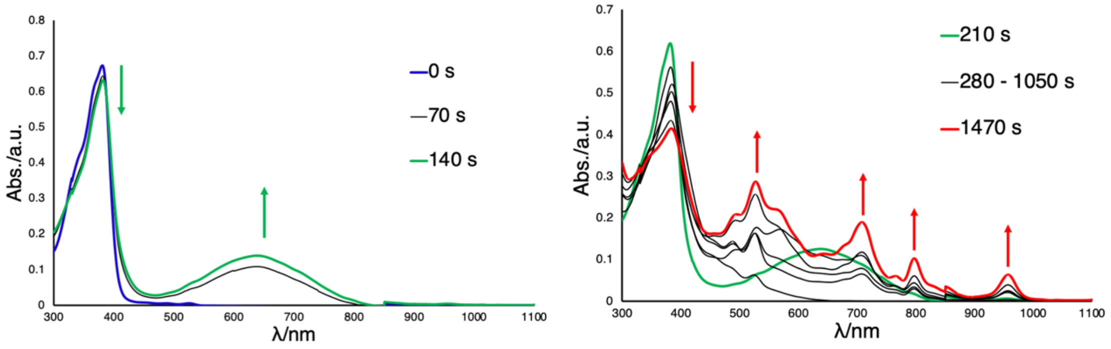

Figure S40. Spectroelectrochemical absorption spectra of **11** in CH_2Cl_2 using $[Bu_4N][PF_6]$ as the supporting electrolyte at −1.2 V (vs Fc/Fc^+).

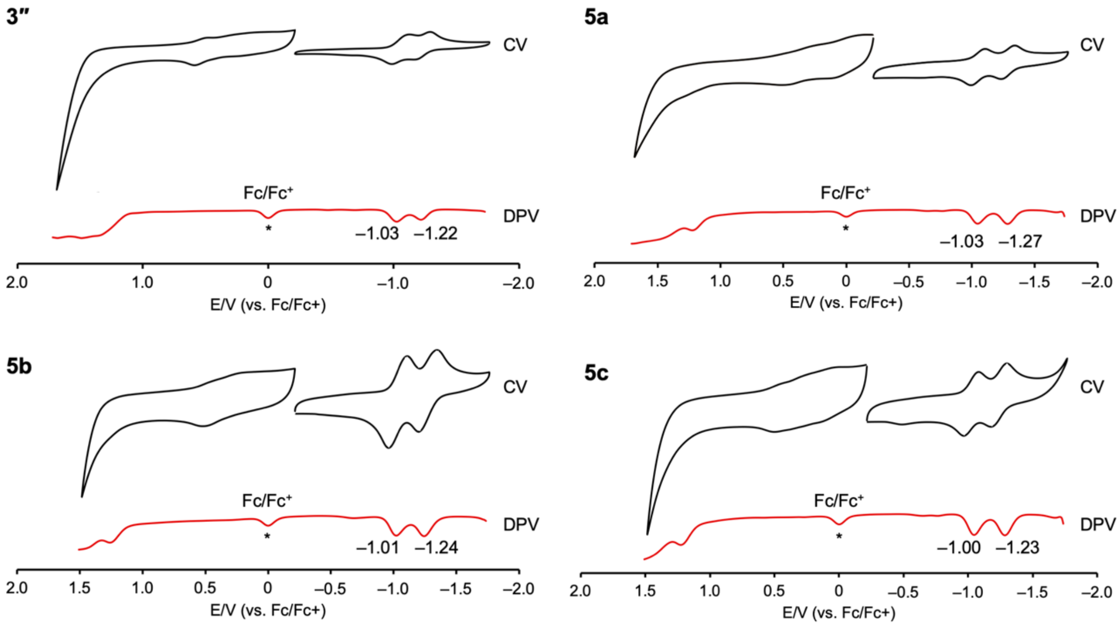

Figure S41. Cyclic voltammograms of **3′**, **5a**, **5b**, and **5c**.

7. Thermal analysis

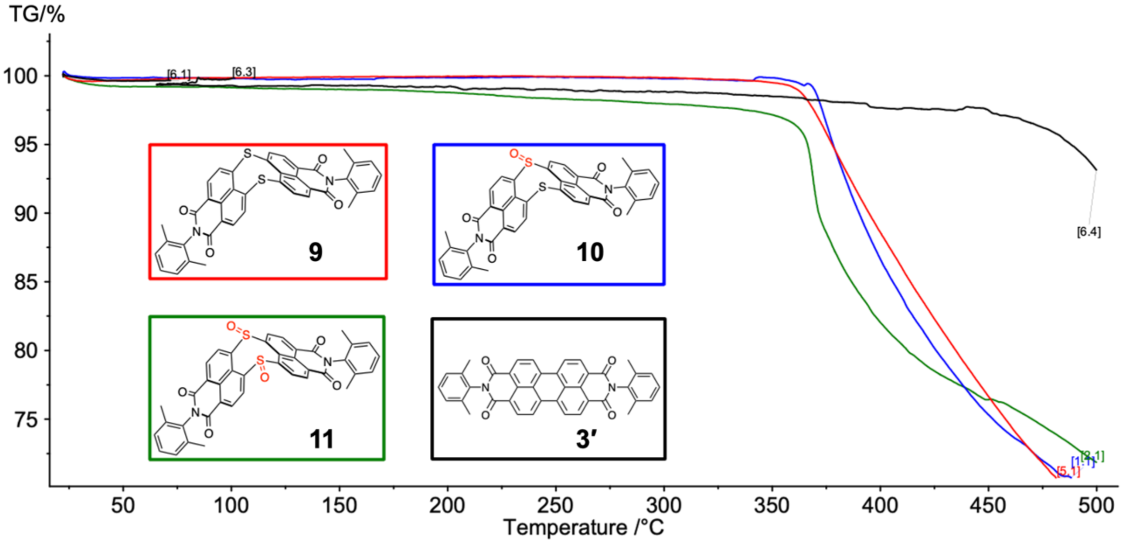

Figure S42. Thermogravimetric analysis (TGA) profiles of **3′**, **9**, **10**, and **11**.

8. DFT Calculations

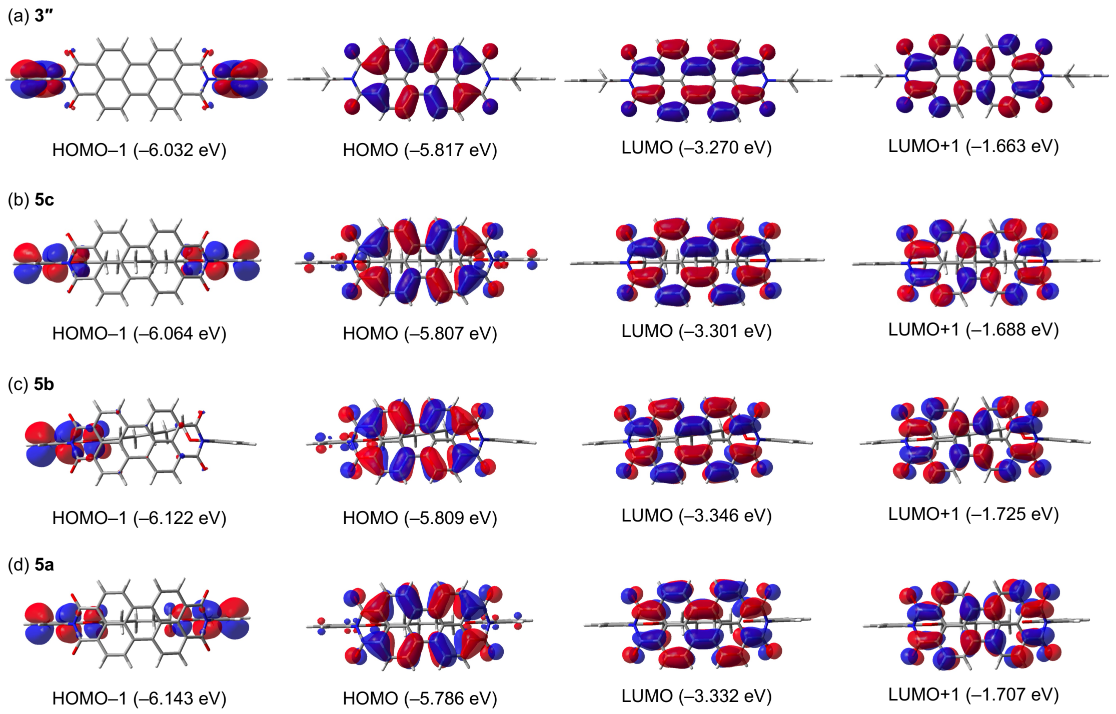

Figure S43. Calculated molecular orbitals of **3″**, **5a**, **5b**, and **5c** (isovalue = 0.02, B3LYP/6-31G(d)).

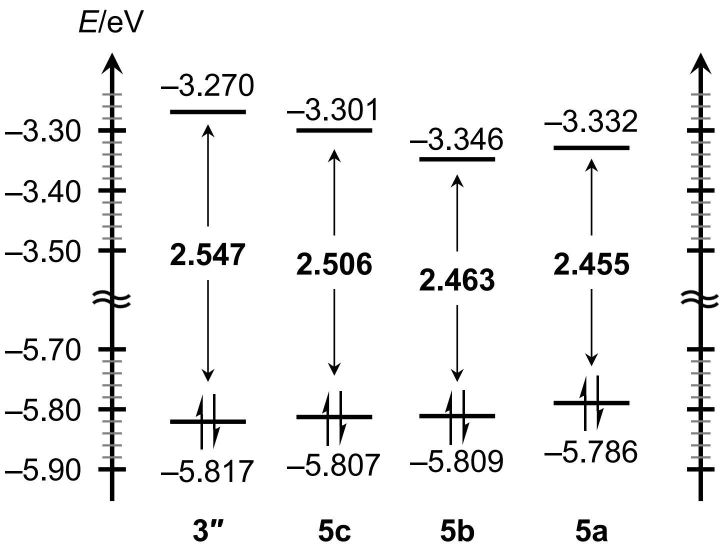

Figure S44. Energy diagrams of **3″**, **5a**, **5b**, and **5c** (B3LYP/6-31G(d)).

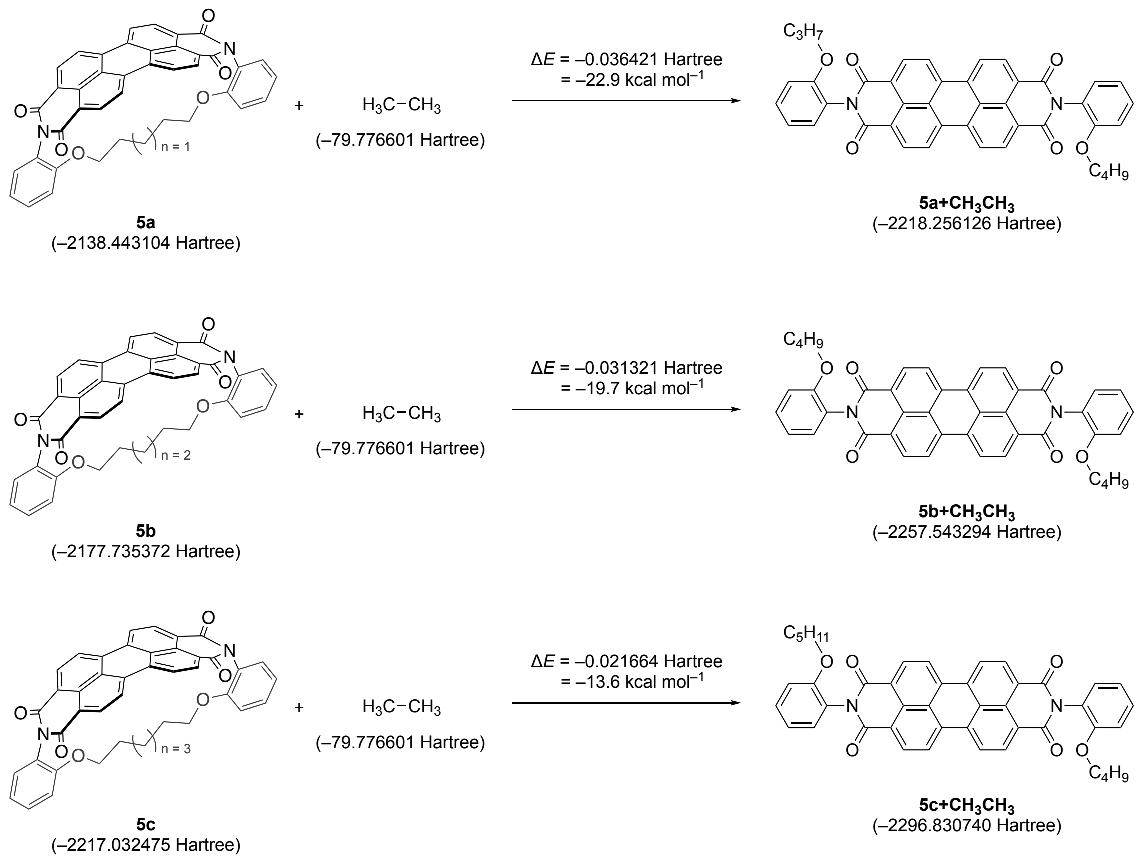

Figure S45. Hypothetical homodesmotic reactions (B3LYP/6-31G(d)).

Table S3. Simulated ^{1}H NMR chemical shifts calculated at the B3LYP/6-31G(d) level. The chemical shifts were averaged for four equivalent protons.

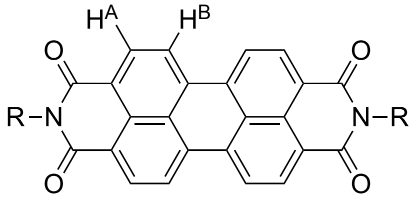

	H^A (ppm)	H^B (ppm)
**5a**	8.49	8.28
**5b**	8.52	8.30
**5c**	8.54	8.35
**3**	8.60	8.40

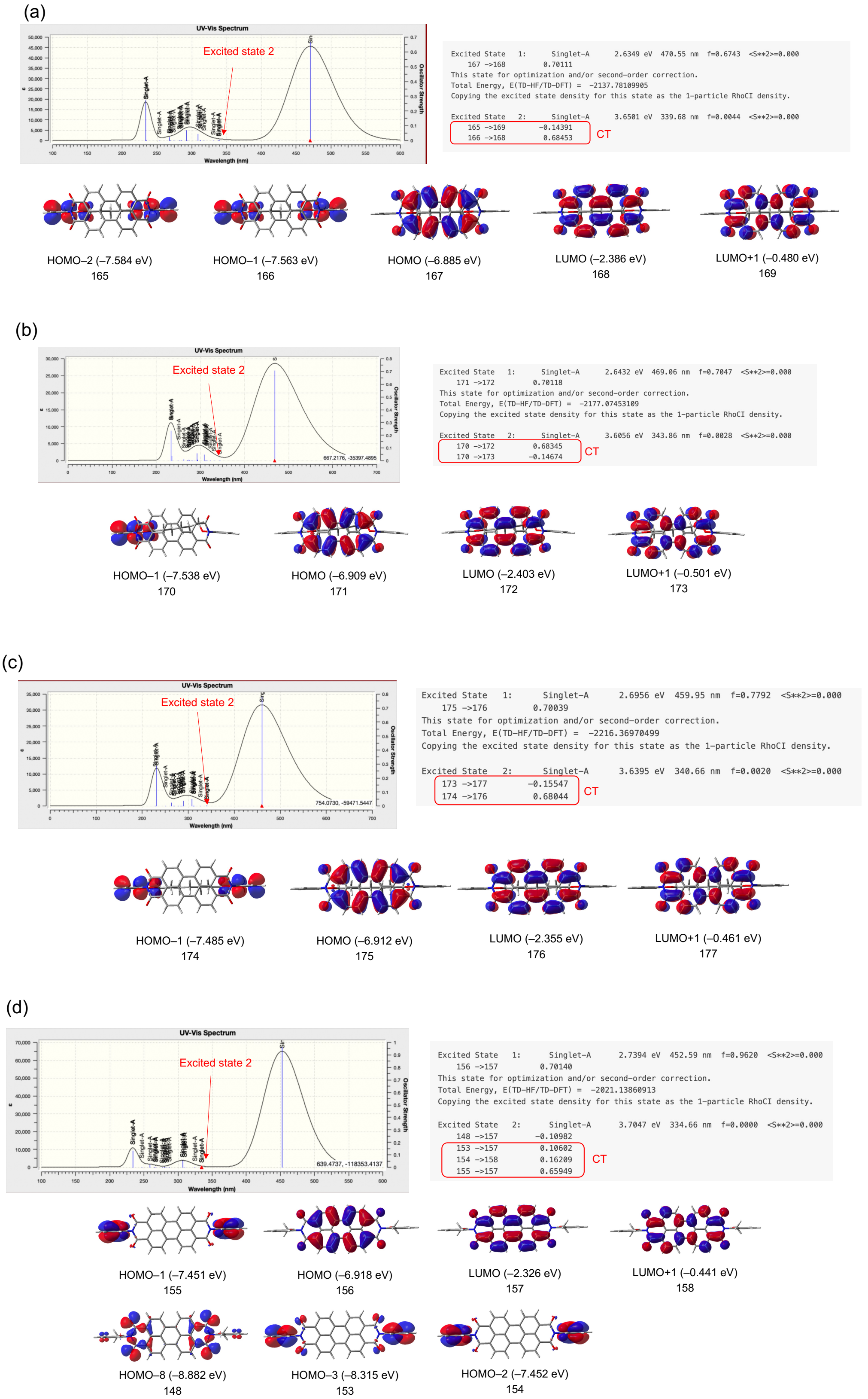

Figure S46. Results of TD-DFT calculations of (a) **5a**, (b) **5b**, (c) **5c**, and (d) **3″**. (B3LYP/6-31G(d)//CAM-B3LYP/6-31G(d)).

Table S4. Cartesian coordinate and geometry of **3″**.

O	6.966945	-0.002646	-2.005822
O	-6.966827	-0.001912	-2.005845
O	5.697872	-2.288734	0.325989
O	5.697776	2.289732	0.322241
N	-5.707245	0.000323	0.380448
C	3.57503	-1.223641	0.345645
O	-5.697805	-2.288948	0.325821
O	-5.697887	2.289522	0.32279
N	5.707226	0.000544	0.380435
C	2.862731	0.000457	0.345739
C	-3.575006	-1.223774	0.34565
C	-2.862753	0.000352	0.345804
C	0.735411	-1.249595	0.344822
C	7.78682	-0.001175	-0.919813
C	-7.147641	0.000267	0.333219
C	-7.786775	-0.000923	-0.919882
C	-3.57505	1.224449	0.343907
C	1.481747	-2.429635	0.344432
H	0.982539	-3.39126	0.344273
C	-9.923338	0.00019	0.215654
H	-11.008618	0.000155	0.160255
C	3.574983	1.224581	0.34374
C	-0.735386	-1.249622	0.344823
C	-1.432681	0.000377	0.344795
C	7.147621	0.000521	0.333247
C	0.735364	1.250427	0.342928
C	2.880576	2.421526	0.340446
H	3.443349	3.348965	0.337632
C	9.184333	-0.001204	-0.970334
H	9.700742	-0.002471	-1.923152
C	-9.184281	-0.00096	-0.97048
H	-9.700652	-0.001878	-1.923318
C	-2.880687	2.421421	0.340657
H	-3.443496	3.348838	0.337951
C	1.481656	2.430493	0.340712
H	0.982414	3.392099	0.339096
C	5.058795	1.250232	0.342369
C	-5.058861	1.250045	0.342642
C	-0.735433	1.2504	0.342969
C	1.432659	0.00043	0.344763
C	9.287508	0.00216	1.454572
H	9.865929	0.003453	2.373118
C	-9.287587	0.001385	1.454424
H	-9.866054	0.002293	2.372942
C	5.05884	-1.249236	0.344388
C	-5.058817	-1.249423	0.344342
C	7.891856	0.002175	1.5047
H	7.368462	0.003475	2.456006
C	-2.880599	-2.420723	0.344153
H	-3.443372	-3.348164	0.342751
C	9.923328	0.00046	0.215831
H	11.008611	0.000423	0.160494
C	-7.891932	0.001417	1.504629

H	-7.368596	0.002331	2.455967
C	-1.481768	2.430439	0.340843
H	-0.982559	3.392062	0.33924
C	2.880666	-2.420617	0.344191
H	3.443474	-3.348037	0.34284
C	-1.481679	-2.42969	0.344409
H	-0.982436	-3.391297	0.344243
C	7.560131	-0.004809	-3.295689
H	8.174175	-0.900734	-3.453446
H	6.730254	-0.006052	-4.004392
H	8.17408	0.890643	-3.456495
C	-7.559927	-0.003432	-3.295751
H	-8.173965	0.892041	-3.456106
H	-6.730006	-0.004191	-4.004404
H	-8.173866	-0.899335	-3.454046

Negative frequency = zero

Sum of electronic and thermal free energies = –2021.807876 Hartree

Table S5. Cartesian coordinate and geometry of **5a**.

O	-3.60828	1.697052	-0.000915
O	3.608299	1.697071	-0.000842
O	-5.291216	-0.545418	2.284631
O	-5.29172	-0.545629	-2.284193
N	5.19382	-0.389193	-0.000097
C	-3.460201	-1.626067	1.224832
O	5.291689	-0.546081	2.28428
O	5.291235	-0.545012	-2.284551
C	0.000015	2.846974	-0.000249
H	0.000245	3.504342	0.880833
H	-0.000206	3.504334	-0.881337
N	-5.19383	-0.389193	0.000215
C	-2.789115	-1.859369	0.00003
C	3.460339	-1.626236	1.224546
C	2.789106	-1.859372	-0.000211
C	-0.734322	-2.380918	1.250594
C	-4.935664	2.015595	-0.000203
C	5.818537	0.910688	0.000112
C	1.262959	1.966427	-0.000598
H	1.247884	1.309193	-0.879135
H	1.248032	1.30865	0.877537
C	4.935702	2.015616	-0.000263
C	-2.598305	2.711803	-0.000163
H	-2.70556	3.347026	0.890526
H	-2.704836	3.347618	-0.89053
C	3.460184	-1.625817	-1.224973
C	-1.48267	-2.313415	2.428285
H	-1.002875	-2.462522	3.388752
C	6.845163	3.490285	0.000849
H	7.244338	4.500989	0.001089
C	-3.460351	-1.625986	-1.224683
C	0.734423	-2.380973	1.250514
C	1.416623	-2.246552	-0.000202
C	-5.818537	0.91069	0.000212
C	-1.262966	1.966462	0.000054
H	-1.248004	1.308752	-0.878128
H	-1.247939	1.309181	0.878549
C	-0.73444	-2.380756	-1.25081
C	-2.828451	-1.927468	-2.419347
H	-3.366034	-1.769263	-3.348402
C	-5.457495	3.309666	-0.000048
H	-4.797082	4.170064	-0.000418
C	5.45755	3.309664	0.000066
H	4.797164	4.170075	-0.000281
C	2.828168	-1.927153	-2.419607
H	3.365633	-1.768764	-3.348704
C	-1.482924	-2.313204	-2.428411
H	-1.003236	-2.462243	-3.38894
C	-4.728927	-0.859353	-1.248794
C	4.728683	-0.859028	-1.249103
C	0.734315	-2.380695	-1.250886
C	-1.416633	-2.246544	-0.000065
C	-7.711917	2.400137	0.000859

H	-8.78662	2.552998	0.001301
C	7.711957	2.400087	0.001312
H	8.786665	2.552921	0.00197
C	-4.728724	-0.859283	1.249098
C	4.728937	-0.859592	1.24879
C	2.598284	2.711775	-0.000427
H	2.705115	3.347528	-0.890801
H	2.70516	3.347071	0.890259
C	-7.190069	1.102585	0.000715
H	-7.840923	0.234554	0.001032
C	2.828424	-1.927897	2.419156
H	3.366011	-1.769866	3.348243
C	-6.845093	3.490315	0.000489
H	-7.244244	4.501028	0.000617
C	7.190069	1.102537	0.000955
H	7.840906	0.234492	0.001338
C	1.482661	-2.31295	-2.428566
H	1.002867	-2.461863	-3.389062
C	-2.828181	-1.92763	2.419406
H	-3.365642	-1.769443	3.34854
C	1.48289	-2.313594	2.428139
H	1.003172	-2.462744	3.388637

Negative frequency = zero

Sum of electronic and thermal free energies = –2138.443104 Hartree

Table S6. Cartesian coordinate and geometry of **5b**.

O	-3.81405	1.63562	-0.157626
O	4.150358	1.946422	-0.259296
O	-5.548973	-0.353018	2.247532
O	-5.489376	-0.852852	-2.291855
N	5.114665	-0.586462	-0.018498
C	-3.682506	-1.503579	1.333846
O	5.08447	-0.189118	2.23563
O	5.246456	-1.180151	-2.228557
C	-0.277563	2.973344	-0.004593
H	-0.452377	3.745466	0.759614
H	-0.243408	3.502203	-0.9682
N	-5.416627	-0.437992	-0.038846
C	-2.99531	-1.862527	0.149236
C	3.254983	-1.413382	1.350553
C	2.597496	-1.848073	0.174581
C	-0.942921	-2.18005	1.465432
C	-5.139334	1.953552	-0.251565
C	6.048127	0.493089	-0.207941
C	1.092202	2.316665	0.261957
H	1.303376	1.562622	-0.506793
H	1.054068	1.779984	1.221089
C	5.51731	1.793031	-0.297392
C	-2.816045	2.66238	-0.184767
H	-3.004645	3.383591	0.623111
H	-2.865151	3.201797	-1.141461
C	3.29411	-1.861635	-1.058015
C	-1.702598	-1.996102	2.622914
H	-1.228707	-2.024792	3.597386
C	7.761788	2.647352	-0.591324
H	8.427626	3.491752	-0.747505
C	-3.657468	-1.783871	-1.099851
C	0.524169	-2.138035	1.47068
C	1.217506	-2.204885	0.22102
C	-6.031213	0.857584	-0.187788
C	-1.456229	1.984409	-0.002825
H	-1.327447	1.242763	-0.801126
H	-1.475646	1.42807	0.943058
C	-0.923945	-2.507685	-1.014012
C	-3.009407	-2.210271	-2.246428
H	-3.53825	-2.163815	-3.192622
C	-5.653014	3.242826	-0.401979
H	-4.988927	4.098397	-0.455108
C	6.38784	2.868842	-0.49153
H	5.98512	3.872602	-0.587898
C	2.663605	-2.336864	-2.195674
H	3.221263	-2.369289	-3.12574
C	-1.66076	-2.582201	-2.198254
H	-1.170335	-2.836167	-3.13085
C	-4.934685	-1.041036	-1.222408
C	4.62785	-1.22869	-1.178595
C	0.544362	-2.526812	-1.000245
C	-1.617084	-2.221495	0.204442
C	-7.911875	2.34386	-0.425154

H	-8.984127	2.498811	-0.493378
C	8.280362	1.35443	-0.508456
H	9.34932	1.184965	-0.594684
C	-4.964947	-0.764953	1.259614
C	4.555477	-0.712367	1.265776
C	2.233356	3.347922	0.30377
H	2.338074	3.829744	-0.677445
H	1.963825	4.143852	1.013595
C	-7.399719	1.051397	-0.274588
H	-8.055216	0.18837	-0.224033
C	2.600787	-1.480898	2.568879
H	3.12859	-1.163591	3.462085
C	-7.037589	3.426161	-0.487283
H	-7.428333	4.433361	-0.604761
C	7.4166	0.275596	-0.320673
H	7.793805	-0.73974	-0.256364
C	1.304753	-2.669864	-2.162956
H	0.830662	-2.965786	-3.091701
C	-3.057421	-1.650762	2.56034
H	-3.608933	-1.401128	3.460699
C	1.255354	-1.85781	2.627601
H	0.762244	-1.834728	3.592361
C	3.605152	2.83354	0.738287
H	4.281084	3.68421	0.882626
H	3.545782	2.289614	1.691052

Negative frequency = zero

Sum of electronic and thermal free energies = –2177.735372 Hartree

Table S7. Cartesian coordinate and geometry of **5c**.

O	5.533952	-0.871727	-2.288384
O	-5.533814	-0.867552	2.288808
O	-5.534158	-0.872258	-2.288239
O	-4.643618	1.661017	-0.002126
O	5.534016	-0.867958	2.288691
O	4.643577	1.660923	-0.000353
N	5.51733	-0.777917	0.000086
N	-5.51723	-0.777845	0.000191
C	-2.836824	-1.722271	0.000975
C	-4.933361	-1.084896	-1.248115
C	-0.735214	-2.038152	-1.249084
C	-1.427121	-1.955265	0.001152
C	-6.000898	1.589223	-0.00202
C	6.509833	0.271197	-0.000845
C	-0.735172	-2.036123	1.251497
C	-2.863762	-1.771186	-2.418714
H	-3.417025	-1.676076	-3.347082
C	4.933334	-1.082612	1.248913
C	-3.535374	-1.582824	-1.222887
C	-7.875048	0.042781	-0.000583
H	-8.239526	-0.979304	0.000397
C	2.836852	-1.722252	0.000892
C	-6.880463	2.670741	-0.003096
H	-6.501949	3.687687	-0.004137
C	0.735245	-2.036148	1.251469
C	4.933276	-1.084666	-1.248259
C	2.863686	-1.771011	-2.418808
H	3.416901	-1.675806	-3.347195
C	3.53534	-1.582692	-1.222997
C	1.483719	-2.000099	2.43006
H	0.992125	-2.092707	3.391451
C	-3.535319	-1.580659	1.224626
C	2.8638	-1.767207	2.420646
H	3.417047	-1.670524	3.348862
C	0.735199	-2.038121	-1.249111
C	3.535404	-1.58075	1.224518
C	-6.509805	0.271193	-0.000775
C	-2.863671	-1.767046	2.420739
H	-3.416874	-1.670284	3.348974
C	-1.483669	-2.004002	-2.427741
H	-0.992057	-2.098086	-3.388978
C	-8.759513	1.12716	-0.00162
H	-9.830911	0.952485	-0.001472
C	-8.258739	2.427264	-0.002865
H	-8.943043	3.27145	-0.003712
C	-1.483597	-1.99999	2.430114
H	-0.991971	-2.092547	3.391494
C	1.483607	-2.003901	-2.427795
H	0.991964	-2.097979	-3.389018
C	-4.933215	-1.082451	1.249032
C	6.000859	1.5892	-0.001263
C	7.875088	0.042844	-0.001556
H	8.239587	-0.979232	-0.001255

C	1.427149	-1.955254	0.001103
C	-3.916113	2.893148	-0.002053
H	-4.169311	3.484818	0.888781
H	-4.165913	3.482789	-0.895207
C	6.880382	2.670755	-0.002465
H	6.501838	3.687689	-0.002857
C	8.258667	2.427337	-0.003168
H	8.942927	3.271559	-0.004097
C	8.759507	1.127258	-0.002709
H	9.830914	0.952638	-0.003282
C	-2.460791	2.429611	0.001264
H	-2.338322	1.77653	-0.872303
H	-2.340404	1.781612	0.878908
C	-1.330823	3.470616	-0.000678
H	-1.39765	4.118269	-0.885923
H	-1.400417	4.125037	0.879353
C	3.916048	2.893047	-0.001091
H	4.166135	3.482263	-0.894441
H	4.168955	3.48513	0.889553
C	2.460735	2.429512	0.002014
H	2.338431	1.776221	-0.871418
H	2.340137	1.781737	0.879791
C	-0.000026	2.699196	0.004272
H	0.000057	2.02678	-0.865955
H	-0.000135	2.037042	0.88232
C	1.330799	3.470562	-0.000404
H	1.397899	4.118081	-0.885728
H	1.400186	4.125105	0.879551

Negative frequency = zero

Sum of electronic and thermal free energies = –2217.032475 Hartree

Table S8. Cartesian coordinate and geometry of **5a+CH_3CH_3**.

O	-7.461078	-1.006329	0.000235
O	7.142053	0.735386	-0.000094
O	-5.740397	1.009149	-2.289423
O	-5.740214	1.010575	2.289107
N	5.419825	-1.341834	0.000121
C	-3.658487	0.595929	-1.224093
O	5.422029	-1.281999	-2.289171
O	5.422102	-1.281099	2.289389
N	-5.737918	1.070418	-0.000177
C	-2.960862	0.451682	-0.000096
C	3.340232	-0.868211	-1.223903
C	2.642664	-0.723338	0.000044
C	-0.879018	0.014089	-1.250074
C	-8.039856	0.223902	0.000005
C	6.839577	-1.590852	0.000149
C	7.979659	1.892285	-0.000152
H	8.625842	1.882933	0.889492
H	8.625906	1.8828	-0.889748
C	7.721424	-0.494496	0.00004
C	-8.298473	-2.162767	0.000385
H	-8.944786	-2.154658	-0.889343
H	-8.944799	-2.154424	0.890104
C	3.340278	-0.867701	1.224025
C	-1.610135	0.164304	-2.430073
H	-1.121971	0.05988	-3.391708
C	9.579438	-2.045605	0.000201
H	10.653037	-2.214088	0.000222
C	-3.658391	0.596684	1.223867
C	0.560744	-0.28647	-1.250035
C	1.243051	-0.42979	0.00001
C	-7.157576	1.319865	-0.000201
C	-0.878934	0.01479	1.249986
C	-2.979315	0.451946	2.420854
H	-3.53075	0.56493	3.348234
C	-9.418242	0.462836	-0.000026
H	-10.11928	-0.363469	0.000129
C	9.099906	-0.733046	0.00007
H	9.800702	0.093453	-0.000017
C	2.661272	-0.72242	2.420977
H	3.212768	-0.834954	3.348374
C	-1.609951	0.165767	2.429951
H	-1.121708	0.061949	3.391612
C	-5.111135	0.899516	1.249273
C	4.793064	-1.170473	1.249487
C	0.560806	-0.285879	1.25002
C	-1.561254	0.158089	-0.000061
C	-9.020741	2.857491	-0.000447
H	-9.398091	3.875303	-0.000617
C	8.703221	-3.127866	0.000305
H	9.080922	-4.145549	0.000408
C	-5.111236	0.898738	-1.24957
C	4.793022	-1.170972	-1.249294
C	-7.644719	2.61946	-0.000422

H	-6.936766	3.44275	-0.000572
C	2.661181	-0.723425	-2.420889
H	3.212648	-0.836318	-3.348259
C	-9.897349	1.775547	-0.000252
H	-10.970887	1.944389	-0.000271
C	7.327118	-2.890284	0.000277
H	6.619428	-3.713803	0.000357
C	1.291893	-0.436272	2.430012
H	0.803702	-0.33201	3.391653
C	-2.979506	0.450453	-2.421044
H	-3.531019	0.562843	-3.348449
C	1.291795	-0.437319	-2.429991
H	0.803569	-0.333447	-3.391656
C	7.07684	3.117879	-0.000275
H	6.423514	3.07233	-0.881171
H	6.423413	3.072425	0.880551
C	7.869377	4.431451	-0.0003
H	8.528839	4.461419	0.878538
H	8.529027	4.461276	-0.879003
C	-7.39581	-3.388644	0.000551
H	-6.743651	-3.342117	-0.879994
H	-6.743426	-3.34168	0.880907
C	-8.196451	-4.694683	0.000981
H	-7.527273	-5.561208	0.000899
H	-8.838496	-4.771557	0.886833
H	-8.83903	-4.771826	-0.884465
C	6.965526	5.66849	-0.000498
H	6.3179	5.685593	0.884346
H	7.555423	6.591751	-0.000499
H	6.318108	5.685459	-0.885497

Negative frequency = zero

Sum of electronic and thermal free energies = –2218.256126 Hartree

Table S9. Cartesian coordinate and geometry of **5b+CH_3CH_3**.

O	7.323942	0.654976	0.000139
O	-7.323815	-0.655177	0.000015
O	5.544969	-1.309376	-2.289395
O	5.544853	-1.310072	2.289145
N	-5.540967	1.370182	-0.000004
C	3.476231	-0.834671	-1.22407
O	-5.544999	1.310145	-2.289282
O	-5.545038	1.309776	2.289264
N	5.540803	-1.370158	-0.000135
C	2.783185	-0.669704	-0.000098
C	-3.476297	0.835204	-1.223994
C	-2.783291	0.669953	-0.000037
C	0.715111	-0.171159	-1.250101
C	7.86656	-0.591552	-0.000001
C	-6.952732	1.661071	0.000009
C	-8.195239	-1.786801	0.000045
H	-8.840882	-1.75826	0.889674
H	-8.840908	-1.758289	-0.889565
C	-7.866609	0.591278	0.00002
C	8.195536	1.786465	0.000235
H	8.841202	1.7579	-0.889375
H	8.841174	1.757776	0.889863
C	-3.476322	0.834986	1.223935
C	1.441445	-0.343123	-2.430082
H	0.956526	-0.224582	-3.391725
C	-9.677981	2.196579	0.000033
H	-10.746128	2.396735	0.000045
C	3.476172	-0.835029	1.223859
C	-0.715163	0.17175	-1.250086
C	-1.392935	0.335259	-0.000052
C	6.952533	-1.661218	-0.000143
C	0.71506	-0.171483	1.249951
C	2.801677	-0.67022	2.420834
H	3.349555	-0.799329	3.348216
C	9.237364	-0.870883	-0.000006
H	9.962341	-0.065489	0.0001
C	-9.237455	0.87042	0.000037
H	-9.962319	0.064927	0.000056
C	-2.801859	0.669938	2.420894
H	-3.349766	0.79884	3.348287
C	1.441334	-0.34381	2.429917
H	0.956367	-0.225554	3.391571
C	4.919371	-1.180609	1.249271
C	-4.919534	1.180509	1.249378
C	-0.715199	0.171488	1.249966
C	1.392835	-0.334981	-0.000083
C	8.769736	-3.252866	-0.000286
H	9.117061	-4.281313	-0.000395
C	-8.770152	3.252467	0.000015
H	-9.11762	4.280866	0.000009
C	4.919435	-1.180231	-1.249513
C	-4.91951	1.180719	-1.249406
C	7.401281	-2.974546	-0.000283

H	6.669466	-3.776703	-0.00039
C	-2.801808	0.670368	-2.420968
H	-3.3497	0.79942	-3.34835
C	9.677708	-2.197105	-0.000148
H	10.745829	-2.397402	-0.000149
C	-7.401662	2.974338	0.000003
H	-6.669959	3.776597	-0.000009
C	-1.44151	0.343551	2.429947
H	-0.956569	0.225099	3.391591
C	2.801794	-0.669513	-2.421029
H	3.34972	-0.798337	-3.348422
C	-1.441454	0.344004	-2.430051
H	-0.956495	0.225718	-3.391706
C	-7.329076	-3.038564	0.000047
H	-6.674705	-3.012476	-0.880865
H	-6.674573	-3.012377	0.880857
C	-8.160187	-4.328081	0.000186
H	-8.820205	-4.338393	0.879058
H	-8.82048	-4.338419	-0.878479
C	7.32956	3.038357	0.000301
H	6.67525	3.012455	-0.880662
H	6.674988	3.012174	0.88106
C	8.16086	4.327753	0.000642
H	8.820794	4.337886	0.879578
H	8.82124	4.338087	-0.877959
C	-7.293397	-5.591355	0.000066
H	-6.646514	-5.627553	0.884877
H	-7.910391	-6.49673	0.000191
H	-6.646816	-5.62759	-0.884964
C	7.294246	5.591149	0.000557
H	6.647767	5.627564	-0.88454
H	6.647272	5.627344	0.885301
H	7.911364	6.49644	0.000845

Negative frequency = zero

Sum of electronic and thermal free energies = –2257.543294 Hartree

Table S10. Cartesian coordinate and geometry of **5c+CH_3CH_3**.

O	7.530352	0.518102	-0.001002
O	-7.160304	-0.225245	-0.001152
O	5.676285	-1.373451	-2.287579
O	5.675351	-1.377835	2.29094
N	-5.298905	1.728396	0.002455
C	3.627146	-0.820332	-1.222179
O	-5.304556	1.671477	-2.286898
O	-5.306074	1.664964	2.291633
N	5.669348	-1.435874	0.001614
C	2.940708	-0.629946	0.001832
C	-3.2561	1.115519	-1.221698
C	-2.570324	0.92215	0.002214
C	0.893509	-0.051334	-1.248051
C	8.023791	-0.748692	0.000032
C	-6.698146	2.074576	0.00248
C	-8.075774	-1.321719	-0.003322
H	-8.720064	-1.269121	0.886138
H	-8.719388	-1.266161	-0.89309
C	-7.653462	1.041601	0.000579
C	8.445555	1.614609	-0.003713
H	9.088829	1.559161	-0.893781
H	9.090339	1.562313	0.885459
C	-3.256886	1.112127	1.226215
C	1.612907	-0.250126	-2.428071
H	1.133034	-0.112346	-3.389683
C	-9.400139	2.717087	0.002491
H	-10.459569	2.959195	0.002483
C	3.626622	-0.822797	1.225752
C	-0.522508	0.346384	-1.247953
C	-1.193847	0.534305	0.002118
C	7.06864	-1.781803	0.001503
C	0.892909	-0.054079	1.252
C	2.958705	-0.633304	2.422763
H	3.501052	-0.78412	3.350122
C	9.382642	-1.081388	-0.000167
H	10.13855	-0.304955	-0.001365
C	-9.012257	1.374597	0.000602
H	-9.768308	0.598306	-0.00084
C	-2.589629	0.919743	2.423128
H	-3.132463	1.068396	3.350549
C	1.61185	-0.255073	2.431925
H	1.131591	-0.119135	3.393606
C	5.055474	-1.223588	1.251086
C	-4.685717	1.513049	1.251729
C	-0.523228	0.34321	1.252095
C	1.564203	-0.242187	0.001927
C	8.822236	-3.443261	0.002811
H	9.129108	-4.484495	0.003926
C	-8.45141	3.736377	0.004366
H	-8.758085	4.777669	0.005827
C	5.055986	-1.221131	-1.247697
C	-4.684892	1.516589	-1.247018
C	7.465711	-3.11168	0.002923

H	6.703106	-3.884623	0.004096
C	-2.588079	0.926442	-2.41871
H	-3.130292	1.077762	-3.346064
C	9.770783	-2.423806	0.001222
H	10.830255	-2.665723	0.001072
C	-7.094951	3.404531	0.004347
H	-6.332193	4.177325	0.005781
C	-1.242794	0.541419	2.432108
H	-0.763055	0.403181	3.393721
C	2.959725	-0.628492	-2.419093
H	3.502436	-0.777562	-3.346521
C	-1.241282	0.547986	-2.427873
H	-0.760919	0.412438	-3.389558
C	-7.259538	-2.60663	-0.005134
H	-6.604278	-2.603846	-0.885667
H	-6.605113	-2.606874	0.876027
C	-8.141272	-3.861838	-0.007709
H	-8.802936	-3.849126	0.871107
H	-8.801872	-3.846253	-0.887276
C	7.629268	2.899447	-0.005277
H	6.973465	2.897416	-0.885506
H	6.975151	2.900695	0.876211
C	8.510466	4.155265	-0.008429
H	9.17145	4.141171	0.869666
H	9.169587	4.138055	-0.887866
C	-7.332065	-5.165462	-0.009347
H	-6.67203	-5.181516	0.869253
H	-6.670925	-5.178629	-0.887164
C	7.694078	5.451672	-0.00986
H	7.048321	5.511753	-0.894193
H	7.050229	5.514907	0.875644
H	8.346242	6.332051	-0.01213
C	-8.211737	-6.419492	-0.011961
H	-7.605316	-7.332203	-0.01308
H	-8.859921	-6.451456	0.872451
H	-8.858809	-6.448547	-0.897288

Negative frequency = zero

Sum of electronic and thermal free energies = –2296.830740 Hartree

Table S11. Cartesian coordinate and geometry of ethane.

C	0	0	0.765306
H	-0.510273	0.884352	1.16449
H	1.021008	-0.000267	1.16449
H	-0.510735	-0.884085	1.16449
C	0	0	-0.765306
H	0.510735	-0.884085	-1.16449
H	-1.021008	-0.000267	-1.16449
H	0.510273	0.884352	-1.16449

Negative frequency = zero

Sum of electronic and thermal free energies = –79.776601 Hartree

9. Photophysical properties

Method to determine quantum yields of the triplet generations: To obtain quantum yields of the intersystem crossings (ISC), we conducted transient absorption (TA) spectroscopic analysis of **5b** and **5c** (Figure SX). The nanosecond transient absorption data were obtained using a nanosecond time-resolved optical absorption spectrometer system (UNISOKU, TSP-1000-KK) at room temperature. For the light excitation of 532 nm light, a Nd: YAG laser (Continuum Minilite II, fwhm = 5 ns, pulse energy = 0.3 mJ/pulse, pulse repetition frequency = 10 Hz) was employed. The excitation wavelength was the second harmonics (532 nm) of the 1064 nm pump laser. The sample solutions were deoxygenated by the freeze-pump-thaw cycle in the CH_2Cl_2 solutions.

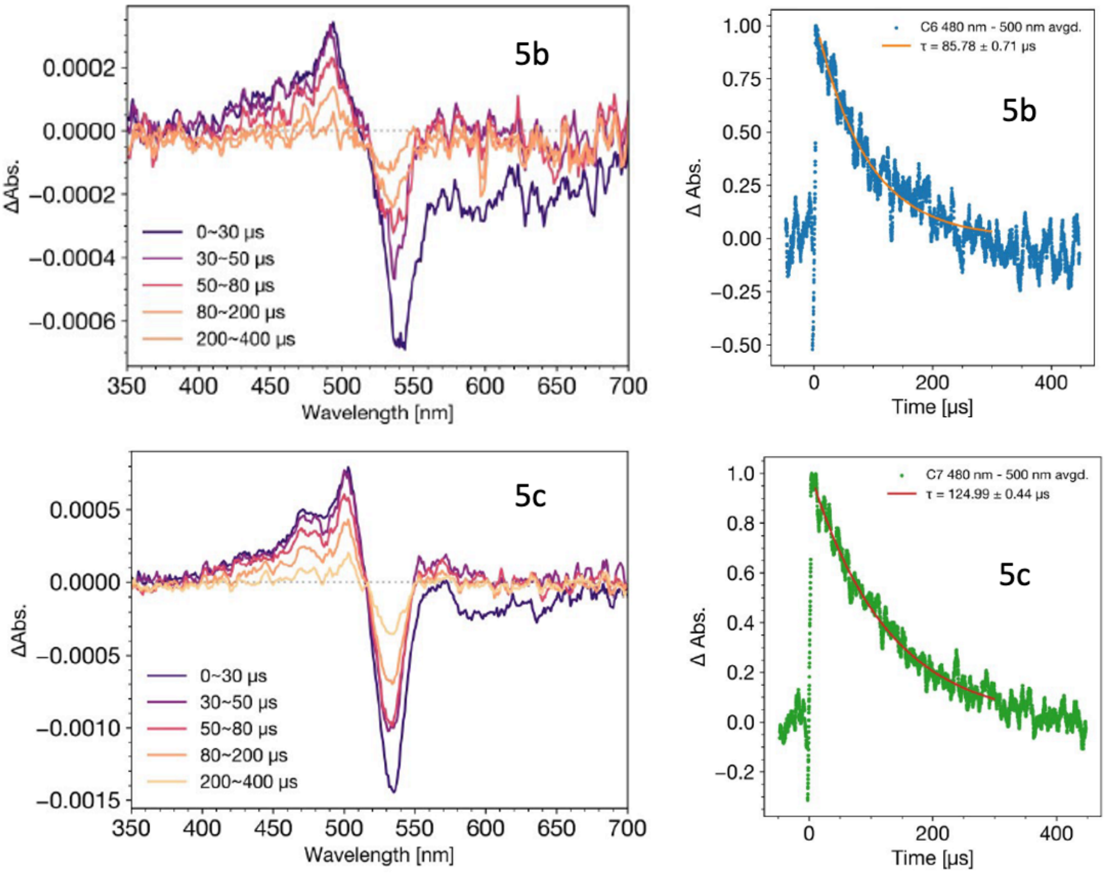

Figure S477. TA spectra of **5b** and **5c** by the 532 nm laser irradiations. Time profiles of the absorbance changes in the 480–500 nm regions are shown in the right panels.

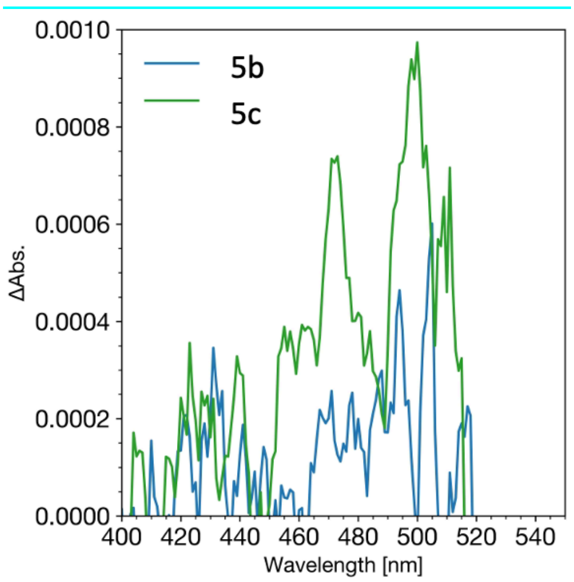

Figure S488. TA spectra of **5b** and **5c** at 1 μs after the 532 nm laser irradiations.

The TA spectra In Figure S47 are almost identical with the reported TA spectra[5] of the excited triplet states of **3‴**. Thus, these bands originate from the triplet exatons generated by the ISC.

The optical densities of the 532 nm absorptions are determined from the light pathlengths of l = 0.1 [cm] in the UV-VIS measurements and the actual path (0.45 cm) employed with the TA measurements. The absorbances were A_{532} = 0.1128 (**5b**) and 0.1259 (**5c**) in the UV-VIS. Thus, for the TA experimental setups, the following absorbances were obtained:

$$A_{532nm,5b} = 0.56655$$

$$A_{532nm,5c} = 0.5076$$

The S_1 state concentration [S_1] is determined using the numbers (mol) of the laser photons ($N_{Ph} = 8.0346... \times 10^{14}$) per pulse and the rate of the absorption ($1 - 10^{-A_{532nm}}$) by the followings:

$$N_{S1,5b} = \frac{N_{Ph} \times \left(1 - 10^{-A_{532}nm,5b}\right)}{N_A} = \frac{8.0346... \times 10^{14} \times (1 - 10^{-0.56655})}{6.022 \times 10^{23}[mol^{-1}]}$$

$$= 9.7256024... \times 10^{-10}[mol]$$

$$N_{S1,5c} = \frac{N_{Ph} \times (1 - 10^{-A_{532,5c}})}{N_A} = \frac{8.0346... \times 10^{14} \times (1 - 10^{-0.5076})}{6.022 \times 10^{23}[mol^{-1}]}$$

$$= 9.1991906... \times 10^{-10}[mol]$$

$[S_1]$ is thus obtained as,

$$[S_1]_{5b} = N_{S1,5b}/V = 3.05754 \ldots \times 10^{-5}[M]$$
$$[S_1]_{5c} = N_{S1,5c}/V = 2.89204\ldots \times 10^{-5}[M]$$

where irradiation volume (V) is estimated from the irradiation path ($0.45[cm]$) and the radius of the laser spot, as, $V = \pi r^2 l = \pi \times 0.15[cm] \times 0.15[cm] \times 0.45[cm] = 3.18086\ldots \times 10^{-5}[L^{-1}]$.

From Figure S49, the absorbance peak tops around 500 nm are $Abs.(\mathrm{T1})_{5b} = 0.0005$ and $Abs.(\mathrm{T1})_{5c} = 0.001$, respectively. Using the reported difference of the molar excitation coefficients $(\varepsilon_T - \varepsilon_{S0}) \approx 40000\,[M^{-1}cm^{-1}]$ between the triplet state and the ground state absorption[5], the triplet concentrations are estimated as follows:

$$[T]_{5b} = \frac{Abs.(TT)_{5b}}{(\varepsilon_T - \varepsilon_{S0}) \times l} = \frac{0.0005}{40000 \times 0.45\ [cm]} = 2.8 \times 10^{-8}[M]$$

$$[T]_{5c} = \frac{Abs.(TT)_{5c}}{(\varepsilon_T - \varepsilon_{S0}) \times l} = \frac{0.001}{40000 \times 0.45\ [cm]} = 5.6 \times 10^{-8}[M]$$

Thus, the triplet yields in Table 2 are estimated as follows:

$$\Phi_{T,5b} = \frac{[T]_{5b}}{[S_1]_{5b}} = \frac{2.8 \times 10^{-8}[M]}{3.05754 \ldots \times 10^{-5}[M]} \approx 0.0009$$

$$\Phi_{T,5c} = \frac{[T]_{5c}}{[S_1]_{5c}} = \frac{5.6 \times 10^{-8}[M]}{2.89204\ldots \times 10^{-5}[M]} =\approx 0.0019$$

10. References

1. Gaussian 16, Revision B.01, M. J. Frisch, G. W. Trucks, H. B. Schlegel, G. E. Scuseria, M. A. Robb, J. R. Cheeseman, G. Scalmani, V. Barone, G. A. Petersson, H. Nakatsuji, X. Li, M. Caricato, A. V. Marenich, J. Bloino, B. G. Janesko, R. Gomperts, B. Mennucci, H. P. Hratchian, J. V. Ortiz, A. F. Izmaylov, J. L. Sonnenberg, D. Williams-Young, F. Ding, F. Lipparini, F. Egidi, J. Goings, B. Peng, A. Petrone, T. Henderson, D. Ranasinghe, V. G. Zakrzewski, J. Gao, N. Rega, G. Zheng, W. Liang, M. Hada, M. Ehara, K. Toyota, R. Fukuda, J. Hasegawa, M. Ishida, T. Nakajima, Y. Honda, O. Kitao, H. Nakai, T. Vreven, K. Throssell, J. A. Montgomery, Jr., J. E. Peralta, F. Ogliaro, M. J. Bearpark, J. J. Heyd, E. N. Brothers, K. N. Kudin, V. N. Staroverov, T. A. Keith, R. Kobayashi, J. Normand, K. Raghavachari, A. P. Rendell, J. C. Burant, S. S. Iyengar, J. Tomasi, M. Cossi, J. M. Millam, M. Klene, C. Adamo, R. Cammi, J. W. Ochterski, R. L. Martin, K. Morokuma, O. Farkas, J. B. Foresman, and D. J. Fox, Gaussian, Inc., Wallingford CT, **2016**.
2. (a) Becke, A. D. *Phys. Rev. A* **1988**, *38*, 3098. (b) Lee, C.; Yang, W.; Parr, R. G. *Phys. Rev. B* **1988**, *37*, 785.
3. Tanaka, Y.; Tajima, K.; Fukui, N.; Shinokubo, H. *Asian J. Org. Chem.* **2021**, *10*, 541
4. Uersfeld, D.; Stappert, S.; Li, C.; Müllen, K. *Adv. Synth. Catal.* **2017**, *359*, 4184.
5. Ford, W. E.; Kamat, P. V. *J. Phys. Chem.* **1987**, *91*, 6373.
